# Climate‐Driven Range Shifts Limit the Role of Non‐Native Trees for Adaptation Processes of European Forests

**DOI:** 10.1002/ece3.73999

**Published:** 2026-07-08

**Authors:** Reneema Hazarika, Debojyoti Chakraborty, Joana Vicente, Harald Vacik

**Affiliations:** ^1^ Institute of Silviculture, University of Natural Resources and Life Sciences (BOKU) Vienna Austria; ^2^ Austrian Research Centre for Forests (BFW) Vienna Austria; ^3^ CIBIO, Centro de Investigação em Biodiversidade e Recursos Genéticos, InBIO Laboratório Associado Campus de Vairão, Universidade do Porto Vairão Portugal; ^4^ BIOPOLIS Program in Genomics, Biodiversity and Land Planning Vairão Portugal

**Keywords:** adaptation, alternative tree species, climate change, ecosystem services, Europe, non‐native trees (NNTs), species distribution models

## Abstract

Climate change threatens biodiversity and ecosystem services by reducing the suitability of many native tree species in Europe. As the ranges of native species contract, non‐native tree species (NNTs) are increasingly considered as potential alternatives in forest management. We analyzed published ensemble species distribution models (SDMs) for 15 non‐native and 13 native tree species at 1‐km resolution to estimate range shifts of the NNTs and compared range shift patterns of NNTs and native species to identify alternative species under climate change. Most coniferous NNTs are expected to lose suitability in southern and southeastern Europe while expanding northward under climate change. In contrast, broadleaved NNTs such as *Juglans nigra* , *Fraxinus pennsylvanica* , and *Robinia pseudoacacia* are likely to retain southern suitability and expand into central and northern regions. Although NNTs are projected to experience a 50%–70% increase in climatic suitability, their role remains limited, as they can replace native species as the most suitable option in only 17%–21% of Europe's forested areas by 2061–2080. Native species are expected to remain dominant across most of Europe, while a limited subset of NNTs may provide complementary options in specific regions. Integrating NNTs into climate adaptation strategies, therefore, requires careful, site‐specific evaluation of ecological risks and benefits.

## Graphical abstract

Ensemble species distribution model based study indicates a limited role of non native tree species as alternatives to vulnerable native tree species in Europe.

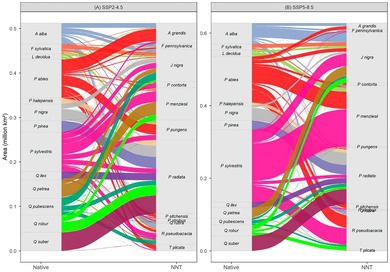

## 1 Introduction

Climate change is already causing widespread shifts in the composition and range of plant communities worldwide (Scheffers et al. 2016). In Europe, the effects of climate change on forests include changes in forest productivity (Reyer et al. 2014), changes in the distribution of tree species (Dyderski et al. 2018; Thurm et al. 2018), the economic value of forests (Hanewinkel et al. 2013), and intensified disturbances (Seidl et al. 2011, 2014) and droughts (Allen et al. 2010). Climate change‐induced disturbance regimes, combined with legacies of past management, such as monoculture and increasing growing stock, are likely to severely impede the ability of European forests to provide multiple ecosystem services, including quality of life and health (Desvars‐Larrive et al. 2024; Neumann et al. 2017; Schelhaas et al. 2003; Seidl et al. 2011, 2014; Thom and Seidl 2016).

Climate change is also expected to cause widespread shifts in the potential ranges of major native tree species across Europe (Chakraborty, Móricz, et al. 2021; Dyderski et al. 2018, 2025; Koch et al. 2022; Noce et al. 2023; Thurm et al. 2018), sparking discussion on the need for alternative tree species (Bolte et al. 2023; Konic et al. 2024; Lévesque et al. 2023; Lindner et al. 2010; Schelhaas et al. 2015). To add to this complexity, Europe also has a limited pool of alternative native tree species compared to North America and Asia due to migration barriers presented by the geographic orientation of the mountain ranges, limiting post‐glacial redistribution of tree genera (Albrecht et al. 2024; Latham and Ricklefs 1993; Svenning and Skov 2004, 2007). Therefore, Non‐native tree species (NNTs) have been discussed as an option to support adaptive forest management and compensate for the potential loss in the provision of ecosystem services resulting from the ongoing reduction in the range of native European tree species (Castro‐Díez et al. 2019; Frischbier et al. 2019; Hazarika et al. 2024; Konic et al. 2024; Spiecker et al. 2019; Thurm et al. 2018).

NNTs have been an integral part of European forestry for a long time (Brundu et al. 2020; Frischbier et al. 2019; Lapin et al. 2023). Brus et al. (2019) documented about 145 NNTs in Europe, mostly originating from North America, Asia, and Australia, dominated by *Robinia pseudoacacia* , *Picea sitchensis* , and *Pseudotsuga menziesii* . A recent estimate by Lapin et al. (2020) identified 530 NNTs in forests and urban areas of the European Alpine space. Although NNTs are widespread in European forests and urban habitats, their introduction and potential spread raise concerns about their negative impacts on native biodiversity (Castro‐Díez et al. 2019; Essl et al. 2020; Kracke et al. 2021; Novoa et al. 2024). These concerns have led to policies such as the European legislation on Invasive alien species calling for their control and management (Langmaier and Lapin 2020; Lapin et al. 2023; Pötzelsberger et al. 2020).

Despite strong arguments against their introduction, several NNTs, such as *P. menziesii, P. sitchensis, Pinus contorta*, and *Thuja plicata*, are cultivated in several parts of Europe (Carrillo‐Gavilán et al. 2012; Nygaard and Øyen 2017; Sykes 2001). Recent pan‐European stakeholder surveys found that several NNTs, including potentially invasive ones like *R. pseudoacacia* and *Q. rubra* , were perceived as valuable for delivering diverse ecosystem services, including disturbance protection, agroforestry, climate adaptation, land improvement, and urban greening (Dimitrova et al. 2022; Hazarika et al. 2024).

Large uncertainties were reported in the extent of available scientific knowledge and public perception to manage the risks and benefits of NNTs in Europe (Dimitrova et al. 2022; Hazarika et al. 2024), highlighting the need for site‐specific risk assessments (Bindewald et al. 2021). A basic requirement of such a site‐specific assessment is the reliable estimate of the potential distribution or climatic suitability of NNTs. Estimating the potential distribution of NNTs is also crucial for assessing their climatically suitable ranges and identifying NNTs that can potentially replace vulnerable native species.

Few recent studies have examined the potential distribution of NNTs across Europe (Albrecht et al. 2024; Puchałka et al. 2023). However, Albrecht et al. (2024) assessed only six NNTs, while Puchałka et al. (2023) relied exclusively on the MaxEnt model. Moreover, both studies produced projections at a spatial resolution of approximately 4 × 4 km, which limits their applicability for practical forest management decisions. Importantly, neither study compared projected range shifts between native and NNT species to evaluate their potential as climate‐resilient alternatives under future climate conditions.

In this study, we address these gaps by (i) estimating the climatically suitable ranges and projected range shifts of 15 NNTs across Europe and (ii) comparing climate suitability between native and NNT species to identify potential alternatives for climate‐resilient forest management. Specifically, we extend previous work (Albrecht et al. 2024; Puchałka et al. 2023) by applying a multimodel ensemble species distribution modeling framework at a finer spatial resolution (1 km). In addition, we include three NNTs, *Acacia dealbata, Picea pungens, and Pinus radiata*, that were not considered in earlier continental‐scale analyses.

## 2 Materials and Methods

### 2.1 Potential Distribution Maps of Non Native Trees (NNTs) and Native Tree Species

To estimate the climatically suitable range and range shifts, we used published maps of the potential distribution of 15 NNTs (Chakraborty et al. 2025; Chakraborty, Dobor, et al. 2021) and 13 widely occurring native species (Chakraborty et al. 2024; Chakraborty, Móricz, et al. 2021) (Table S1). These maps depict the climatically suitable potential distribution of the target species as probability of occurrence (0–1), predicted by ensemble Species Distribution Models (SDMs) at a spatial resolution of 30‐arc secs, which is roughly equivalent to 1 km depending on latitude.

### 2.2 Species Occurrence Data

The occurrence data for the native species were from (Mauri et al. 2017), while the occurrence data for the 15 NNTs in their native range and introduced range in Europe were obtained from various sources, such as National Forest Inventories (Mauri et al. 2017) and data collected by the EU COST Action NNEXT (Table S2). This occurrence data includes records from both the native and introduced range of the target NNTs, representing a wide range of environmental conditions (Figure S1). These datasets have a spatial precision of around 1 km. (see detailed description of the SDMs in Supporting Information). The occurrence data were treated to reduce sampling bias and to match the climate data's resolution (Tables S3 and S4).

### 2.3 Bioclimatic Variables

The bioclimatic variables to calibrate the SDMs for NNTs comprised annual, seasonal, and monthly variables from Worldclim2.0 (Fick and Hijmans 2017), while the bioclimatic variables for SDMs of native species were from the ECLIPS 2.0 dataset (Chakraborty, Dobor, et al. 2021), both with a spatial resolution of 30 arc‐sec (Tables S5 and S6). These bioclimatic variables represent historical climate (1961–1990) and two Shared Socioeconomic Pathways (SSP) scenarios used in IPCC's Sixth Assessment Report, SSP2‐4.5 and SSP5‐8.5, for the period 2041–2060, 2061–2080, and 2081–2100 (IPCC 2021; Riahi et al. 2017). To represent likely future climate scenarios, the bioclimatic variables projected by 13 Global Circulation Models (GCMs) available in the Worldclim2.0 dataset (Fick and Hijmans 2017) and 5 GCMs from the ECLIPS.2.0 (Chakraborty, Dobor, et al. 2021), the dataset was used.

### 2.4 Ensemble Species Distribution Models (SDMs)

The SDMs for both NNTs and native species were calibrated with observed occurrence locations (presence and absence) as dependent variables and bioclimatic variables as independent variables, implemented with the R package biomod2 (Thuiller et al. 2016). The biomod2 offers a computational platform for 10 modeling algorithms to calibrate SDMs for each target species.

Three sets of SDMs were calibrated for each NNT species, each with the observed occurrence and bioclimatic variables of the NNTs in their (1) native range, (2) non‐native or introduced range in Europe, and (3) combination of both native and non‐native distribution range. However, we based our analysis on the potential distribution of NNTs calibrated with occurrence data from both native and non‐native ranges.

We used the 10 species distribution modeling (SDM) algorithms implemented in biomod2 (Thuiller et al. 2016) along with an ensemble approach, to predict the potential distributions of both native species and non‐native tree species (NNTs). For each species, ensemble predictions were generated by calculating the median probability across models that met a performance threshold of True Skill Statistic (TSS > 0.7). The individual SDMs and the resulting ensemble models were first used to map potential distributions under historical climate conditions (1961–1990). The ensemble models were then projected to map the potential distribution in the future climate scenarios SSP2‐4.5 and SSP5‐8.5 for 2041–2060, 2061–2080 and 2081–2100 using the 13 GCMs from the WorldClim 2.0 dataset (Fick and Hijmans 2017) for NNTs and 5 GCMs from the ECLIPS 2.0 dataset (Chakraborty, Dobor, et al. 2021).

For comprehensive reporting, we restricted our analysis to historic (1961–1990) and one future time frame, 2061–2080 for SSP2‐4.5 and SSP5‐8.5 scenarios. In this study, the maps of the potential distributions of native and NNTs were restricted to only those grid cells of Europe where at least 50% of the area is covered by forest according to European Forest Cover data (Gunia et al. 2016). We termed these grid cells the “forested grid cells” of Europe.

Detailed description of the SDMs for both native species and NNTs in Supporting Information, along with model evaluation statistics (Tables S7–S10). In addition, detailed information on the SDMs according to the ODMAP framework: Overview, Data, Model, Assessment, and Prediction (Zurell et al. 2020) is also available in (Chakraborty et al. 2025) and (Chakraborty et al. 2024) for the NNT and native species, respectively.

### 2.5 Estimation of the Range Shift of NNT Species

To estimate the shift in climatically suitable ranges of the 15 NNTs, we converted the probabilities of occurrence predicted by the ensemble SDMs to binary: presence and absence (1 or 0), following thresholds of probability or cutoff values based on True Skill Statistics (TSS) reported by (Chakraborty et al. 2025) (Table S11). To convert continuous probability of occurrence values into binary presence–absence predictions, biomod2 identifies the cutoff value that maximizes TSS on the evaluation dataset (i.e., where the sum of sensitivity and specificity is highest). This “maximum TSS threshold” is then used to classify model outputs into predicted presences (probability ≥ cutoff) and absences (probability < cutoff). This approach balances omission and commission errors, offering an optimal, prevalence‐independent threshold for binarizing SDMs, and is now standard in ensemble modeling frameworks such as biomod2 (Allouche et al. 2006; Liu et al. 2005; Thuiller et al. 2016).

Thereafter, range overlap, range contraction, and range expansions in km^2^ were estimated for each of the 15 NNT species as:

Range overlap=if historic distribution=1;future distribution=1

Range contraction=if historic distribution=1;future distribution=0

Range expansion=if historic distribution=0;future distribution=1

### 2.6 Comparing the Climatically Suitable Areas of the Best‐Suited NNT and Native Species

This analysis focused only on NNTs such as *Abies grandis, P. menziesii, Juglans nigra, P. contorta, Fraxinus pennsylvanica, P. pungens, P. sitchensis, Pinus strobus, P. radiata, Q. rubra, R. pseudoacacia, and T. plicata*, which have been discussed as potential alternatives to vulnerable native tree species and are perceived to offer multiple ecosystem services by a wide range of stakeholders in Europe (Hazarika et al. 2024; Lapin et al. 2023; Pötzelsberger et al. 2020).

For each such forested grid cell of Europe, the native species and the NNTs having the highest probability of occurrence (Figures S3 and S5), which we refer to as “best‐suited species,” were identified and mapped. Thereafter, the total area covered by each best‐suited species was calculated. We also calculated the area where native species were best suited under historic climate but replaced by NNTs in future scenarios of SSP2‐4.5 and SSP5‐8.5 in 2061–2080.

Since the estimation of the climatic suitability of all target species within all the 25 million forested grid cells at 30 arc‐sec resolution of Europe is computationally intensive and difficult to comprehend, we summarized the climatic suitability of all the species within each bioclimatic zone of Europe (Metzger et al. 2005). For each target species, we focused on forested grid cells with climatic probability values at or above the species‐specific threshold, which were considered climatically suitable for that species (Table S11). For each species‐bioclimatic region and climate scenario combination, zonal statistics were computed using exact area weighting via the exactextractr R package (Baston 2025) which accounts for partial pixel overlap with polygon boundaries, yielding more accurate estimates than centroid‐based approaches. Thereafter, we computed statistics, including the mean, median, minimum, and maximum of climatic suitability values per bioclimatic region.

To summarize climatic suitability within each bioclimatic region, we calculated an area‐weighted composite suitability score. First, the proportion of forested grid cells exceeding the species‐specific threshold (Table S11) was multiplied by the total area of the bioclimatic region to estimate the absolute suitable area:

Area.above=proportion above threshold×area of the bioclimatic region

We then multiplied this suitable area above the species‐specific threshold by the regional mean climatic suitability to obtain a composite score.

Score=mean suitability×Area.above

Within each region, native and NNT species were ranked based on their composite scores. This composite score ranks the species that are both highly suitable and widely distributed, while down‐weighting species that are suitable only in small areas. Similar area‐weighted approaches have been used in previous European tree suitability assessments (Buras and Menzel 2019; Hanewinkel et al. 2013).

### 2.7 Uncertainty in the Range Shift of NNTs and Best‐Suited Species

To account for uncertainty in projected range shifts of NNTs, as well as the area covered by the best‐suited native and NNT species, we derived the standard deviation of the potential distribution of the 10 SDM algorithms and corresponding ensemble models under historical climate (Thuiller et al. 2016). Uncertainty in the future estimation of range shift and area covered by best‐suited species was based on standard deviation from 13 GCMs from the WorldClim 2.0 dataset and 5 GCMs from the ECLIPS 2.0 dataset for the period 2061–2080 under the SSP2‐4.5 and SSP5‐8.5 scenarios. Maps of standard deviation in the potential distribution of the NNTs are presented in Figures S9–S23.

All statistical analyses and data visualizations were performed using R (R Core Team 2024).

## 3 Results

### 3.1 Potential Geographical Distribution and Range Shifts of NNTs in Europe

**FIGURE 1:**
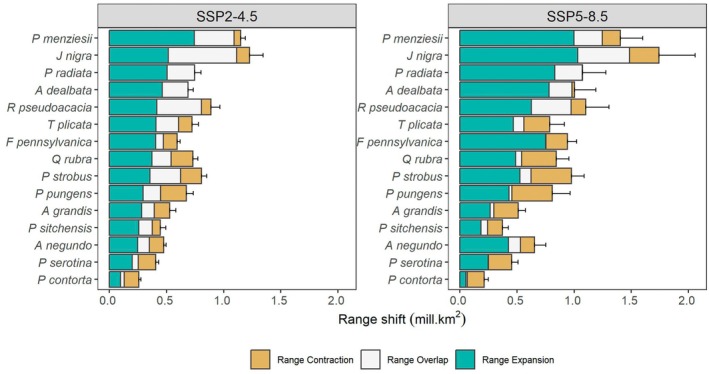
Range shift of the 15 NNTs in Europe. The range shift was estimated by an ensemble SDM model with occurrence data from both native and non‐native ranges of each target species. Range shift (contraction, overlap, and expansion) refers to changes in potential species range between the historic period (1961–1990) and the future time frame 2061–2080 under two climate change scenarios, SSP2‐4.5 and SSP5‐8.5. Error bars represent the sum of the standard deviation of range shift area (contraction, overlap, and expansion) computed with the 10 SDM algorithms of the biomod 2 framework under the historic period, and the 13 GCMs of the worldlcim2.0 dataset for which potential distribution was estimated.

**FIGURE 2:**
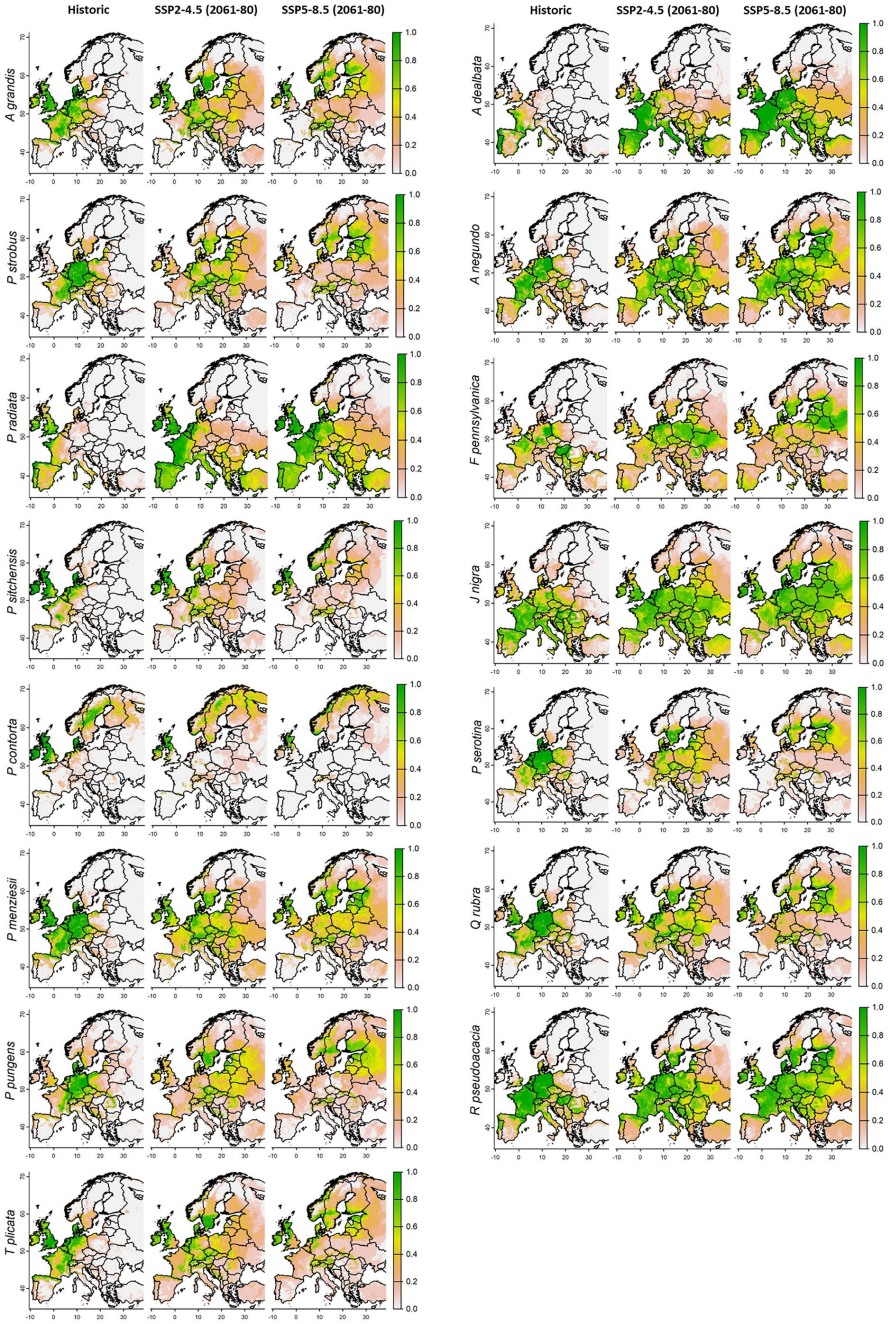
Potential distribution of the NNTs in Europe under historic (1961–1990) and future time frames (2061–2080) under two emission scenarios, SSP2‐4.5 and SSP5‐8.5. The potential distribution is represented by the probability (0–1) of the occurrence of the respective NNTs estimated by ensemble SDM calibrated with occurrence data from both native and non‐native ranges of the respective species. Future distributions represent the mean value of the 13 ensemble SDMs, each predicted with a GCM available within the Worldclim2.0 dataset.

Conifers such as *P. menziesii, P. radiata, T. plicata* and *P. strobus* and broadleaf NNTs such as *J. nigra, A. dealbata, R. pseudoacacia* and *F. pennsylvanica* are predicted to experience the highest range expansion in the future, ranging from approximately 0.5 to 1 million km^2^ (Figure 1). Under climate change all coniferous NNTs are predicted to experience contraction or loss of their historical range in the south and southeast Europe while showing a range expansion in the north (Figure 2). Broadleaved species such as *J. nigra* , *A. dealbata, Acer negundo, R. pseudoacacia*, and *F. pennsylvanica* are expected to retain most of their historic range in the south while expanding into new ranges in central and northern Europe in the future (Figure 2). *Abies grandis, P. contorta, P. sitchensis, and T. plicata* are expected to confine themselves to the Atlantic regions of the British Isles, coastal Scandinavia, and the Atlantic part of Germany and Denmark, and the Netherlands in the future (Figure 2).

### 3.2 Climatic Suitability of Native and NNTs at the Continental Scale in Europe

Historically, native species dominate the European landscape, with a predicted climatically suitable area of approximately 2.43 ± 0.01 million km^2^, significantly higher (Wilcoxon signed‐rank test, *p* < 0.005) than the 0.489 ± 0.008 million km^2^ suitable for NNTs (Table 1). Under future climate scenarios (SSP2‐4.5 and SSP5‐8.5), a divergent trend emerges that native species are projected to lose 10%–14% of their best‐suited range, whereas NNTs are predicted to expand by 50%–70% (Table 1). Widespread native conifers such as *Abies alba, P. abies*, and *Larix decidua* are predicted to lose a large part of their climatically suitable range in future scenarios (Table 1, Figures S4 and S5). While native broad‐leaved species such as *Fagus sylvatica, Q. robur, Quercus pubescens*, and some conifers such as *Pinus sylvestris* and *Pinus halepensis* are predicted to gain climatically suitable range in the future (Table 1, Figures S4 and S5). NNTs such as *P. menziesii, J. nigra, P. radiata, P. sitchensis*, and *R. pseudoacacia* are predicted to gain a larger climatically suitable range in the future compared to their historic range. However, NNTs such as *A. grandis, P. contorta, F. pennsylvanica*, *P. pungens* , *P. strobus* , and *T. plicata* are likely to experience a decline in the climatically suitable range in the future (Table 1).

**TABLE 1:** The potential climatically suitable areas and their change in future scenarios of the best‐suited NNT and native species in Europe.

Species	Area in million km^2^			Change in area between historic and future scenarios (%)	
	Historic (1961–1990)	SSP2‐4.5 (2061–2080)	SSP5‐8.5 (2061–2080)	SSP2‐4.5 (2061–2080)	SSP5‐8.5 (2061–2080)
*A. grandis*	0.029 ± 0.003	0.050 ± 0.007	0.020 ± 0.004	73.2% ± 6.2%	−31.4% ± 7.0%
*P. menziesii*	0.043 ± 0.002	0.090 ± 0.004	0.177 ± 0.013	108.9% ± 2.8%	310.1% ± 14.5%
*J. nigra*	0.067 ± 0.002	0.127 ± 0.004	0.198 ± 0.009	89.4% ± 1.4%	193.9% ± 6.0%
*P. contorta*	0.106 ± 0.012	0.040 ± 0.006	0.012 ± 0.003	−62.1% ± 1.7%	−88.8% ± 1.3%
*F. pennsylvanica*	0.069 ± 0.002	0.015 ± 0.001	0.008 ± 0.001	−78.6% ± 0.5%	−88.4% ± 0.6%
*P. pungens*	0.030 ± 0.002	0.051 ± 0.004	0.004 ± 0.000	69.2% ± 2.8%	−88.0% ± 0.7%
*P. radiata*	0.039 ± 0.002	0.247 ± 0.015	0.243 ± 0.023	530.6% ± 9.4%	520.1% ± 27.8%
*P. sitchensis*	0.008 ± 0.000	0.018 ± 0.001	0.019 ± 0.002	128.7% ± 5.5%	144.1% ± 15.0%
*P. strobus*	0.021 ± 0.002	0.009 ± 0.001	0.002 ± 0.000	−56.2% ± 1.0%	−89.7% ± 0.7%
*Q. rubra*	0.012 ± 0.000	0.004 ± 0.000	0.003 ± 0.000	−64.5% ± 0.5%	−72.8% ± 1.0%
*R. pseudoacacia*	0.060 ± 0.003	0.069 ± 0.005	0.145 ± 0.015	15.5% ± 2.2%	142.7% ± 11.9%
*T. plicata*	0.005 ± 0.000	0.016 ± 0.001	0.002 ± 0.000	235.1% ± 4.4%	−47.0% ± 1.6%
*A. alba*	0.137 ± 0.009	0.003 ± 0.000	0.001 ± 0.000	−97.6% ± 0.1%	−99.4% ± 0.0%
*F. sylvatica*	0.032 ± 0.001	0.055 ± 0.002	0.043 ± 0.003	70.7% ± 2.3%	33.3% ± 4.1%
*L. decidua*	0.028 ± 0.002	0.004 ± 0.000	0.002 ± 0.000	−85.5% ± 0.5%	−91.6% ± 0.7%
*P. abies*	0.793 ± 0.019	0.486 ± 0.020	0.282 ± 0.018	−38.7% ± 1.1%	−64.4% ± 1.4%
*P. sylvestris*	0.858 ± 0.018	0.942 ± 0.019	0.915 ± 0.028	9.9% ± 0.7%	6.7% ± 1.5%
*Q. petrea*	0.048 ± 0.002	0.000 ± 0.000	0.007 ± 0.001	−99.7% ± 0.0%	−85.7% ± 0.7%
*Q robur*	0.041 ± 0.002	0.295 ± 0.016	0.352 ± 0.028	611.9% ± 10.2%	751.3% ± 31.5%
*P. halepensis*	0.039 ± 0.002	0.036 ± 0.003	0.045 ± 0.005	−7.7% ± 2.0%	15.8% ± 7.4%
*P. nigra*	0.155 ± 0.004	0.125 ± 0.006	0.116 ± 0.008	−19.6% ± 1.5%	−25.7% ± 3.0%
*P. pinea*	0.030 ± 0.001	0.002 ± 0.000	0.003 ± 0.000	−92.3% ± 0.1%	−90.6% ± 0.4%
*Q. ilex*	0.051 ± 0.001	0.042 ± 0.001	0.039 ± 0.002	−17.6% ± 1.2%	−23.5% ± 1.7%
*Q. pubescens*	0.167 ± 0.009	0.183 ± 0.014	0.265 ± 0.031	10.0% ± 2.5%	59.2% ± 10.0%
*Q. suber*	0.059 ± 0.003	0.024 ± 0.002	0.032 ± 0.004	−59.3% ± 1.1%	−45.3% ± 3.6%
Native	2.438 ± 0.019	2.198 ± 0.032	2.102 ± 0.048	−10.0% ± 0.6%	−14.0% ± 0.9%
NNT	0.489 ± 0.008	0.736 ± 0.062	0.832 ± 0.058	50.0% ± 3.2%	69.8% ± 4.3%

*Note:* Best‐suited species refer to species having the highest climatic suitability determined by the probability of occurrence. Values after ± represent the standard deviation of suitable area estimates from 10 SDM algorithms of the biomod 2 framework under the historic period, and the 13 GCMs of the worldclim2.0 dataset for NNTs and 5 GCMs from ECLIPS.20 climate dataset for native species.

**FIGURE 3:**
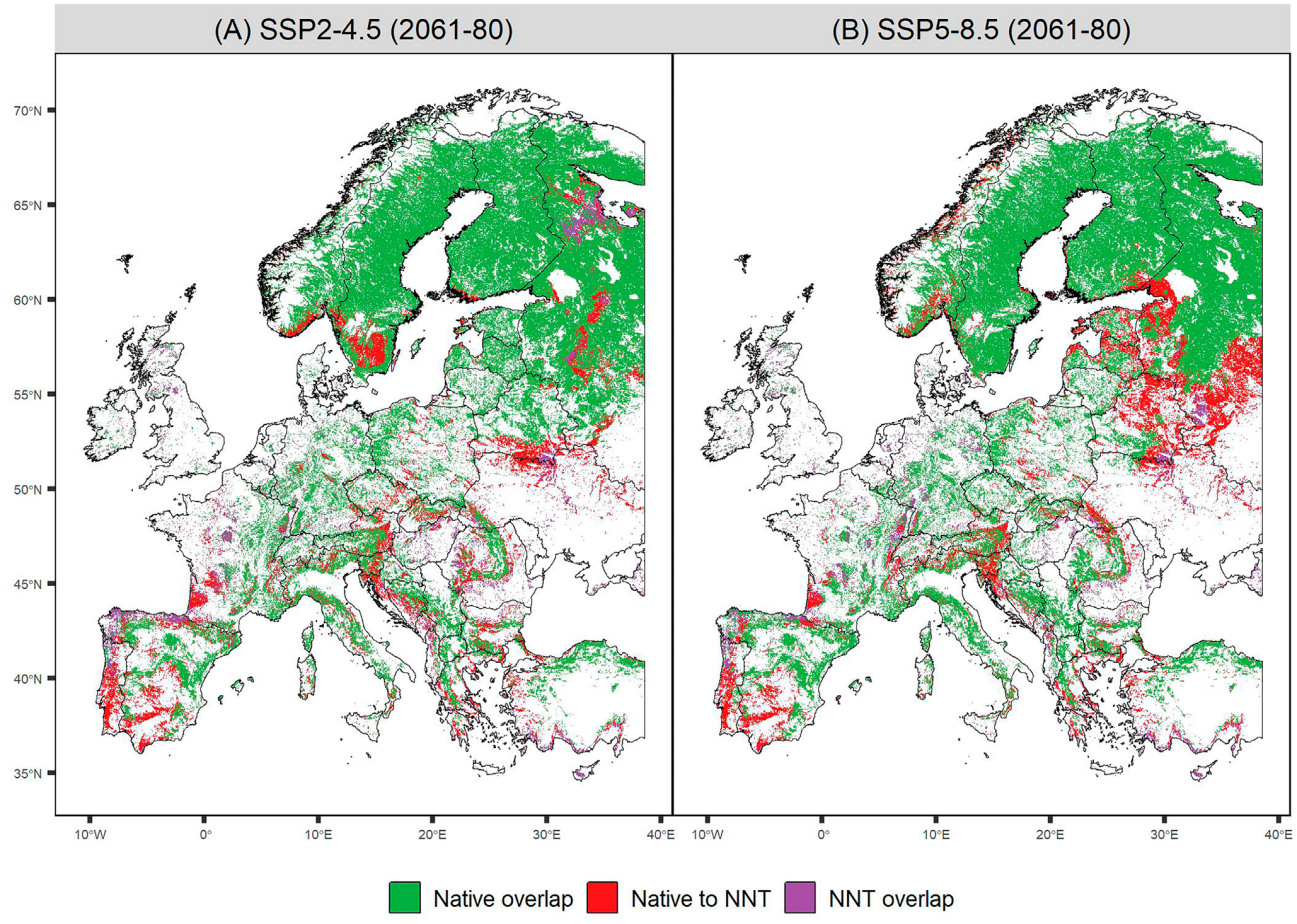
Transition of best‐suited species from historic to climate change scenarios SSP2‐4.5 (A) and (B), SSP5‐8.5. Native overlap and NNT overlap refer to native or NNTs being best‐suited in both historic and climate change scenarios. Native to NNT and NNT to Native refers to a transition of best‐suited species from historic (1961–1990) to climate change scenarios of SSP2‐4.5 and SSP5‐8.5. Future distributions represent the mean value of the 13 ensemble SDMs, each predicted with a GCM from the Worldclim2.0 dataset and 5 GCMs for the native species from the ECLIPS.2.0 dataset.

**FIGURE 4:**
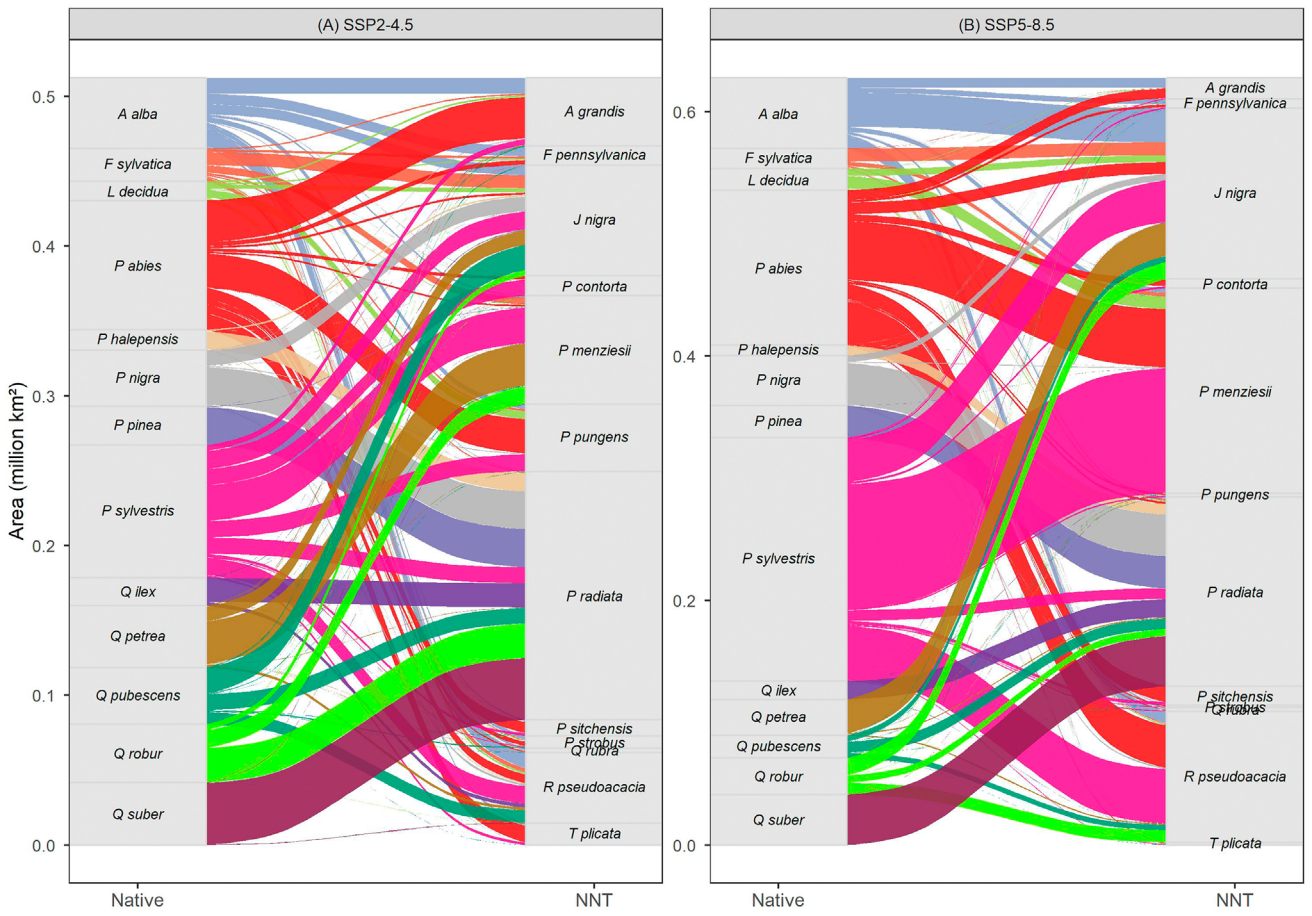
Climatically suitable area of the Native species in historic climate (left columns) predicted to be replaced by NNTs (right columns) in two climate scenarios, SSP2‐4.5 and SSP5‐8.5, for the period 2061–2080. Future distributions represent the mean value of the 13 ensemble SDMs, each predicted with a GCM from the Worldclim2.0 dataset and 5 GCMs for the native species from the ECLIPS.2.0 dataset.

Despite the expansion in area where NNTs are climatically best‐suited in the future (Table 1), the native species remain the “best‐suited” in 62%–66% of forested grid cells of Europe (Table 2). Only in 17%–21% of forested grid cells, NNTs are projected to replace native species as the climatically best‐suited option by 2061–2080 (Table 2, Figure S5). In these grid cells, primarily located in the nemoral and southern Mediterranean regions of Europe (Figure 3), NNTs, such as *A. grandis, J. nigra, P. menziesii, P. radiata, and R. pseudoacacia*, are predicted to replace the climatically suitable range lost by widespread native species, like *A. alba, P. abies, and P. sylvestris*, in the future (Figure 4).

**TABLE 2:** Change in best‐suited species from the historic period to climate change scenarios of SSP2‐4.5 and (B) SSP5‐8.5, represented by the suitable area in million km^2^.

Transition of the best‐suited species	Climatically suitable area in million km^2^		% of total forest area	
	Historic to SSP2‐4.5	Historic to SSP5‐8.5	Historic to SSP2‐4.5	Historic to SSP5‐8.5
Native overlap	1.89 ± 0.74	1.78 ± 0.13	66.03% ± 2.75%	61.09% ± 4.47%
Native to NNT	0.56 ± 0.63	0.61 ± 0.13	17.06% ± 2.03%	21.02% ± 5.08%
NNT overlap	0.20 ± 0.19	0.16 ± 0.34	7.02% ± 1.03%	6.04% ± 1.30%

*Note:* Native and NNT overlap refers to native species and NNTs being best suited in both historical and climate‐change scenarios. Native to NNT refers to a transition of the best‐suited species from native species in the historic period to NNT in climate change scenarios. Values after ± represent the standard deviation of suitable area estimates from 10 SDM algorithms of the biomod 2 framework under the historic period, and the 13 GCMs of the worldclim2.0 dataset for NNTs and 5 GCMs from ECLIPS.20 climate dataset for native species.

Substantial uncertainty exists regarding the area covered by the best‐suited species depending on the climate scenario; for instance, NNTs such as *A. grandis, P. pungens*, and *T. plicata* show projected range gains under SSP2‐4.5 but experience declines under the more severe SSP5‐8.5 scenario (Table 1). Among native species, this contrasting trend between scenarios is observed only for *P. halepensis* (Table 1). Furthermore, the projected transition of forested grid cells from native to NNT dominance under SSP5‐8.5 carries an uncertainty of ±5.08%, reflecting the variability in potential future forest composition shifts (Table 2).

### 3.3 Regional Trends in Climatic Suitability of Native and NNT Species

**FIGURE 5:**
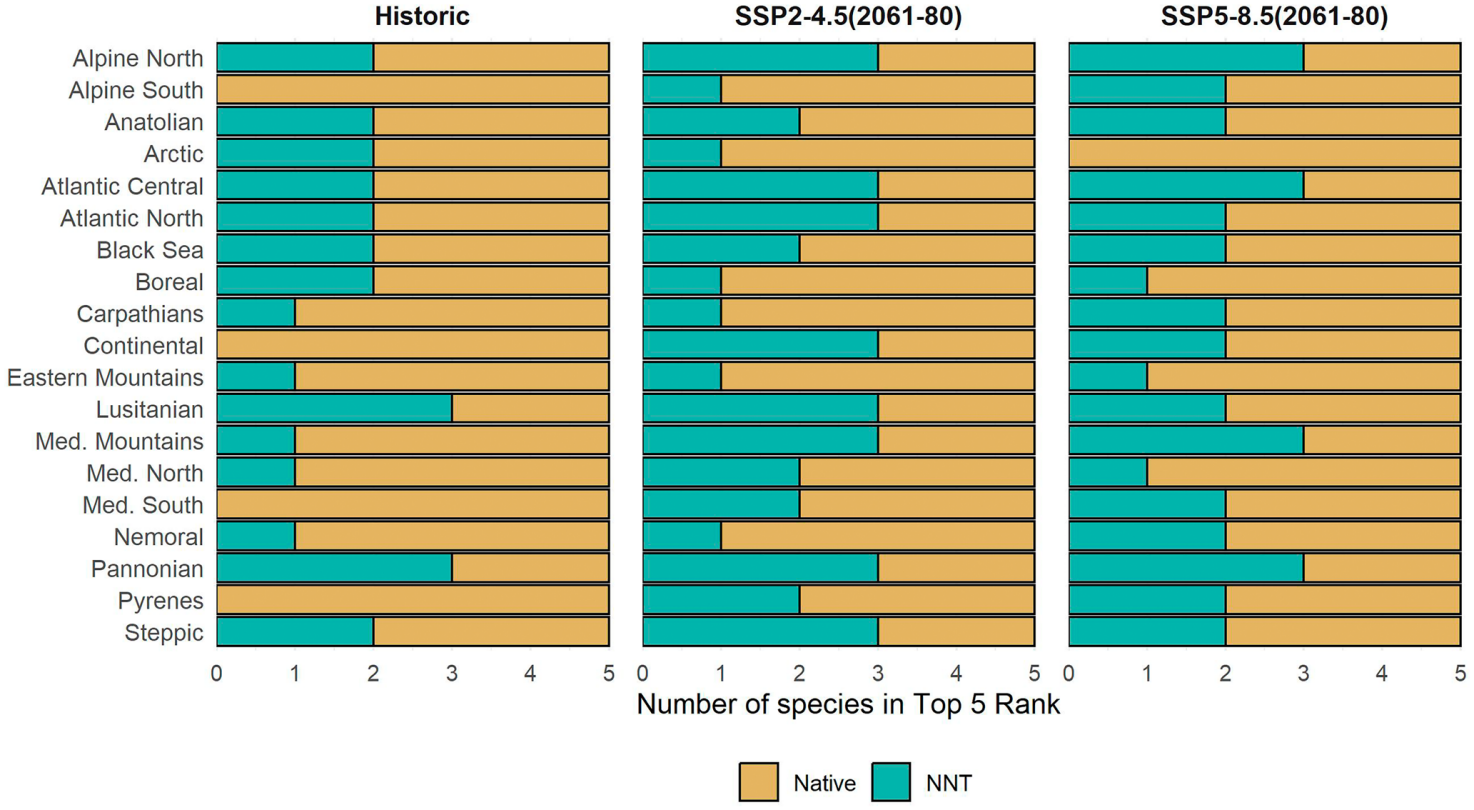
Number of native and NNT species within the top 5 ranks in each bioclimatic region of Europe under historic and two climate scenarios, SSP2‐4.5 and SSP5‐8.5 in 2061–2080. Future distributions represent the mean value of the 13 ensemble SDMs, each predicted with a GCM from the Worldclim2.0 dataset and 5 GCMs for the native species from the ECLIPS.2.0 dataset.

In the historical climate, the native species consistently dominate among the top 5 species in most bioclimatic regions of Europe (Figure 5, Figure S7 and Table S12). Under SSP2, the native and NNTs undergo a moderate reshuffling within the top five species, but native species generally retain their higher ranks. NNTs increased slightly in representation, particularly in Central European regions, yet rarely displaced natives from the top rank. Under SSP5, shifts became more pronounced, most notably in Atlantic central, Mediterranean mountains, and Pannonian regions, where NNTs occupied 2–3 out of the top five species. Despite this shift, native species most often retained their dominant ranks across regions. Alpine regions displayed the greatest stability, with minimal changes in ranking across scenarios. In contrast, Mediterranean regions exhibited declining median suitability for some native species, though NNTs did not consistently achieve top rank positions.

Overall, the regional trends in species ranking suggest gradual compositional change rather than abrupt replacement. Native species remain dominant across scenarios, but stronger climate forcing (SSP5) increases the competitive positioning of NNTs, particularly in Central and parts of Northern Europe. Regional differences are evident, with Alpine systems showing resilience, Central Europe showing the strongest increase in NNT prominence, and Mediterranean regions displaying increased variability under climate change. In the regional level, as well as a few NNTs such as *P. menziesii, P. radiata*, *R. pseudoacacia* , *Q. rubra, and J. nigra* emerge as possible alternatives in the future across Europe (Figure 5 and Figure S7).

## 4 Discussion

Understanding how climate change may reshape the distribution of both native and non‐native tree species is essential for anticipating future forest composition and identifying potential alternatives for climate‐resilient forestry. We studied the climatically suitable potential distribution of NNTs and major native tree species of Europe to suggest alternative species under climate change. Our estimates of the potentially suitable climatic range are based on ensemble SDMs utilizing two large datasets combining inventories from both the native and non‐native ranges of the target NNTs. By focusing only on the forested grid cells in Europe, we expect our results to support the identification of suitable alternative species under climate change for the current site conditions.

### 4.1 Range Shift of the NNTs in Europe

Under both climate scenarios, SSP2‐4.5 and SSP5‐8.5, the NNTs follow a similar trend in their ranks of range contraction, overlap, and expansion (Figure 1). In general, the broadleaved NNTs have higher range expansion compared to range contraction (Figure 1), as also reported by previous studies (Albrecht et al. 2024; Puchałka et al. 2023; Thurm et al. 2018).

Among the conifers, *P. menziesii, P. radiata, T. plicata, and P. strobus* ranked among the species with the highest range expansion in the future (Figure 1). This is not surprising, as these conifer species are extensively cultivated NNTs in Europe for timber production, owing to their rapid growth and high drought tolerance (Carrillo‐Gavilán and Vilà 2010; Hermann and Lavender 1999; Puchałka et al. 2023; Salinas‐Bonillo et al. 2024; Spiecker et al. 2019; Wilczyński et al. 2025). Consequently, they are frequently employed in reforestation and commercial forestry, particularly as substitutes for Norway spruce within climate adaptation strategies (Hermann and Lavender 1999; Klimo et al. 2000; Nicolescu et al. 2023; Schüler and Chakraborty 2021; Spiecker et al. 2019). While not widely invasive, concerns exist about its natural regeneration in parts of Germany, Austria, and the UK. Studies in southwestern Germany indicate limited spread beyond plantations, but monitoring is ongoing (Bauhus and Bindewald 2017; Bindewald and Michiels 2018; Lange et al. 2022). Although fairly resilient to drought and pests, the climatic distribution of *P. menziesii* (Figure 2) and recent studies suggest that frequent drought will lead to *P. menziesii* losing substantial range in Central Europe, where it has been widely planted (Chakraborty et al. 2015; Thurm et al. 2018; Schüler and Chakraborty 2021; Jastrzębowski et al. 2021; Puchałka et al. 2023). Under climate change, all coniferous NNTs are predicted to experience contraction or loss of their historical range, particularly in the south and southeast Europe, while a range expansion is predicted in the northeast (Figure 2), which was also reported by Puchałka et al. (2023). Although concerns about several coniferous NNTs persist among forest and conservation stakeholders in Europe (Hazarika et al. 2024), scientific evidence indicates that the majority of these species pose a relatively low risk of invasion (Carrillo‐Gavilán and Vilà 2010). This is also because the cultivation of coniferous NNTs in Europe is associated with significant damage by native and introduced pests and pathogens (Nicolescu et al. 2023; Pietras et al. 2018; Pötzelsberger et al. 2021).

Among the broadleaved NNTs, *J. nigra* , *A. dealbata, A. negundo, R. pseudoacacia, Q. rubra*, and *F. pennsylvanica* are predicted to retain most of their historic range in the south while expanding into new ranges in central and northern Europe in the future (Figures 1 and 2), as also confirmed by Thurm et al. (2018) and Puchałka et al. (2023). Despite concerns for invasion (Langmaier and Lapin 2020; Pötzelsberger et al. 2020), *J. nigra, R. pseudoacacia*, and *Q. rubra* are considered important NNTs, providing a wide range of ecosystem services (Hazarika et al. 2024; Konic et al. 2024).

### 4.2 Can NNTs Replace Vulnerable Native Species Under Climate Change

The rise in climate‐driven forest disturbances in Europe (Grünig et al. 2026) combined with maladapted post‐disturbance regeneration (Potterf et al. 2026) and a narrow pool of suitable native species (Wessely et al. 2024), highlights the urgent need for alternative species to ensure forest resilience. Our projections indicate a pronounced vulnerability of native conifers to climate change, with substantially greater losses of climatically suitable areas compared to native broadleaved species at a continental scale (Table 1), corroborating earlier findings (Chakraborty et al. 2024; Dyderski et al. 2018, 2025; Hanewinkel et al. 2013). Except for *P. sylvestris* and *P. halepensis* , economically important and widely distributed conifers, including *A. alba* , *P. abies* , *L. decidua* , *Pinus nigra* , and *Pinus pinea* , are projected to undergo marked range contractions (Table 1). In contrast, broadleaved species such as *F. sylvatica* and native oaks ( *Q. robur* , *Q. pubescens*) are projected to expand their climatically suitable areas. Such native broadleaved species could offset approximately 62%–66% of the projected range losses of native conifers, particularly in central, Atlantic, and northern Europe (Table 2, Figure 5). At the European continent level, a mere 17%–21% of the lost range of native species can be replaced by NNTs, especially in south‐southeastern, continental, and nemoral regions (Figure 5, Table 2). At the regional level, we also observed an overall dominance of native species across most bioclimatic regions of Europe. Under climate change, despite a gradual gain in prominence of NNTs, especially in the Atlantic central, Mediterranean mountains, and Pannonian regions, the native species still tend to top the list of climatically best‐suited (Figure 5).

Despite the relatively higher climatic suitability of the native broadleaved species under climate change, their ability to colonize the lost ranges of native conifers will depend on dispersal constraints and management interventions (Meier et al. 2012; Svenning and Skov 2004). Climatic suitability alone does not guarantee natural colonization, and in some regions, assisted migration may be required (Svenning and Skov 2004). Even where native broadleaves replace declining conifers, such compositional shifts would entail considerable ecological and economic consequences, particularly for timber supply and associated ecosystem services implications (Hanewinkel et al. 2013; Mina et al. 2017; Gustafson et al. 2023).

Only a small subset of NNTs, *A. grandis* , *J. nigra* , *P. menziesii* , and *P. radiata* qualify as potential alternatives under climate change, further emphasizing the tree species bottleneck highlighted by Wessely et al. (2024). Among alternative non‐native trees (NNTs), *P. menziesii* stands out as a prominent example, having been introduced to Europe over a century ago (Hermann and Lavender 1999). This species is projected to experience substantially greater range expansion than the native *P. abies* (Figures S6 and S7), highlighting its potential as a climate‐resilient alternative, particularly when site‐adapted provenances are employed (Isaac‐Renton et al. 2014; Krakowski and Stoehr 2009; Nicolescu et al. 2023; Schüler and Chakraborty 2021; Spiecker et al. 2019). Moreover, regulatory frameworks and concerns about invasiveness further constrain the deployability of NNTs as viable alternative species under climate change. Furthermore, the contribution of NNTs to sustaining ecosystem services is not widely known (Cantarello et al. 2017; Castro‐Díez et al. 2019; Konic et al. 2024; Mina et al. 2017).

### 4.3 Limitations of the Study and the Way Forward

Modeling species distribution is associated with uncertainties arising from basic assumptions behind the SDMs, presence‐absence data, model algorithms, and climate change scenarios. SDMs assume that occurrence data represent the full range of environments where a species can exist. It is also known that the locations and species compositions of forests in Europe reflect the results of human influence and forest management, indicating that the forests have undergone a strong transformation throughout history (Boivin et al. 2016; Serra‐Diaz et al. 2017). The SDMs used in this study (Chakraborty, Móricz, et al. 2021; Chakraborty et al. 2024) tried to address this issue by using the presence data of native species from a large‐scale harmonized NFI dataset in Europe (Mauri et al. 2017) and by combining presence data from both the introduced range and native range of the target NNTs (Chakraborty et al. 2025). In addition, the pseudo‐absences used in the SDMs are sampled from geographically and climatically distant locations from the presences to remove sampling bias.

Alien species often undergo ecological niche shifts and exhibit non‐equilibrium occupancy patterns when introduced to new environments, such that neither native nor introduced ranges alone fully capture the realized climatic niche (Broennimann and Guisan 2008; Ørsted and Ørsted 2019). Relying solely on native‐range data may therefore miss these shifts and lead to underestimation of potential distributions in the introduced range, whereas calibrating SDMs only with introduced‐range data risks anchoring models to a truncated niche shaped by introduction history, dispersal lags, and intensive management booth (Booth and Jovanovic 2025; Canelles et al. 2021). In line with this, Liu et al. (2022) showed that models developed with combined native and introduced occurrences often exhibit higher predictive performance and improved transferability when niche changes occur, while Davis et al. (2024) found that SDMs using both native and non‐native data more effectively support risk assessment and management planning for alien species.

Thus, higher TSS values from models calibrated with combined native and introduced data (Figure S2) should not be interpreted as evidence of greater ecological realism or guaranteed transferability, as they can partly reflect sampling artifacts and the mixing of heterogeneous, non‐equilibrium records. Therefore, we use TSS as a relative model performance metric to compare alternative calibrations (native‐only, introduced‐only, combined) within a uniform ensemble framework, not as proof of “true” niche recovery (Thuiller et al. 2016). In our case, all NNTs showed higher TSS when calibrated with combined native and introduced occurrences (Figure S2), which we interpret as an indication that pooled data provide the best fit among the three tested options, rather than as a guarantee of perfect ecological accuracy. This contrasts with recent continent‐scale analyses of NNTs in Europe, such as Puchałka et al. (2023), which did not exploit combined native & introduced datasets and may therefore under‐represent parts of the realized niche occurring outside the current European range.

Due to genetic bottlenecks, drift, and silvicultural practices, the full genotypic and phenotypic variability of the NNTs expressed in their native range is unlikely to have been transferred to Europe (Chakraborty et al. 2015; Dyderski et al. 2018). Consequently, even using combined native and introduced occurrences and achieving higher TSS, our models should be interpreted as estimates of potential climatic suitability at the species level, conditional on appropriate genetic material and management, rather than as predictions that all climatically suitable areas will be occupied by the genotypes currently present in Europe (Isaac‐Renton et al. 2014).

The thresholds to convert continuous probability values to binary can strongly influence predicted distributions, adding to their uncertainty (Hellegers et al. 2025). In this study, we based thresholds on maximizing TSS, which is commonly used (Albrecht et al. 2024; Koch et al. 2022; Mauri et al. 2022) because it balances sensitivity and specificity, minimizing omission and commission errors. TSS‐based thresholds can potentially inflate predicted ranges, especially for rare species or low‐prevalence datasets; its use for defining thresholds for widespread species is reasonable since the risk of overprediction due to sparse occurrence data is lower (Hellegers et al. 2025). Moreover, using TSS aligns with the default approach in biomod2, ensuring reproducibility and comparability with other ensemble SDM studies. Performance‐based thresholds such as TSS are generally preferred over arbitrary cut‐offs like 0.5 because they explicitly consider both omission and commission errors (Liu et al. 2005). Nevertheless, predicted range sizes remain sensitive to the threshold choice, so binary maps should be interpreted cautiously.

For comprehensive reporting, we restricted the predictions for the underlying SDMs of native species and NNT only to forest areas defined by grids having at least 50% canopy cover based on (Gunia et al. 2016). Although the threshold of 50% canopy cover might miss potentially biodiversity‐rich areas with a lower canopy cover, a stricter threshold has been preferred to ensure a lower probability of classification errors, as forest areas are more strictly separated from agroforestry areas and urban parks (Friedl et al. 2002; Hansen et al. 2013).

The range shifts (contradiction, expansion, and overlap) of the NNTs might also be influenced by the spread and number of presence data of the NNTs. If this is true, we can expect a larger range expansion compared to NNTs having only a small number of occurrences in the dataset. Our results are not likely to be affected by this scenario since we did not find a significant correlation between the geographic spread of the occurrence data and range expansion, contraction, and overlap (Figure S8).

Another source of uncertainty in our results arises from the fact that the underlying SDMs are based on climatic averages, while extreme meteorological conditions (e.g., frost, drought) and biotic factors (e.g., pests, pathogens) are known to influence the potential distribution of tree species (Seidl et al. 2011, 2014). Therefore, our results should also be interpreted with caution, as the underlying species distribution models (SDMs) do not account for other abiotic factors such as soil conditions, microclimate, or seed dispersal and intraspecific competition among tree species.

## 5 Conclusion

Our study highlights that climate change will substantially alter the distributional ranges of both native and non‐native tree species (NNTs) across Europe. While most native conifers are projected to lose climatically suitable areas, especially in southern regions, many broadleaved natives and several NNTs are likely to expand their ranges, particularly towards central and northern Europe. Despite the loss of range in native species and a projected gain in the range of NNTs, their role as possible alternative species under climate change remains limited. We found that only a small number of native and NNT species seem to be available for forest management in the future, highlighting the need for further research to estimate the tree species suitability for different sites and soil conditions. However, careful evaluation of ecological risks and management implications remains essential when integrating NNTs into future forest strategies.

## Author Contributions

**Reneema Hazarika:** conceptualization (lead), data curation (supporting), formal analysis (lead), investigation (lead), methodology (lead), writing – original draft (lead), writing – review and editing (lead). **Debojyoti Chakraborty:** conceptualization (supporting), data curation (supporting), formal analysis (supporting), methodology (supporting), writing – original draft (supporting), writing – review and editing (supporting). **Joana Vicente:** data curation (lead), methodology (supporting), writing – original draft (supporting), writing – review and editing (supporting). **Harald Vacik:** conceptualization (supporting), formal analysis (supporting), investigation (supporting), methodology (supporting), resources (lead), supervision (lead), writing – original draft (supporting), writing – review and editing (supporting).

## Funding

This work was supported by Interreg.

## Conflicts of Interest

The authors declare no conflicts of interest.

## Supporting information


**Table S1:** Non‐native tree species (NNTs) and Native tree species were included in the study. NNTs with * represent those used to compare the climate suitability of native and NNTs to identify potential alternatives for forest management under climate change.
**Table S2:** Sources of occurrence data for NNTs collected by EU Cost Action NNEXT. The COST Action NNEXT compiled occurrence (coordinates in latitude and longitude) of the target NNTs from various sources described below.
**Table S3:** Number of presence and pseudoabsences used to calibrate the SDMs for NNTs with occurrence data from (i) native range of the species, (ii) introduced range in Europe, (iii) combined dataset with both native and introduced range occurrences.
**Table S4:** The number of presence and absence records used to calibrate the SDMs for the native species.
**Table S5:** The subset of bioclimatic climate variables from the Worldclim2.0 dataset used as predictor variables for developing the SDMs for NNTs.
**Table S6:** Bioclimatic variables from the ECLIPS.20 dataset retained after variable selection to be used to calibrate the SDMs for the respective native species.
**Table S7:** Statistics for evaluation of the modeling algorithms of biomd2 used to develop the ensemble SDM for the NNTs. In the analysis models, calibrated with all (i.e., occurrence data from native & introduced range) of the respective NNTs were used.
**Table S8:** Statistics for evaluation of the ensemble SDM for the target NNTs calibrated with native, non‐native, and all (native + introduced range of the restive NNTs) occurrence data.
**Table S9:** Statistics for evaluation for each of the models used to develop the ensemble SDM for the native species.
**Table S10:** Statistics for evaluation of the ensemble SDM for the 13 native tree species.
**Table S11:** Cut‐off values used by biomod2 to convert probabilities (0–1) to binary values of presence = 1 and absence = 0 by maximizing TSS. These cut‐off values of the ensemble models were only used to convert the predicted probability of occurrence of NNTs to estimate their range shifts in each 1 × 1 km forested grid cell of Europe.
**Figure S1:** Distribution of the presence locations of the 15 NNT species. Red points indicate the observed native distribution of the respective species, while blue points indicate the observed presence locations in their introduced range in Europe.
**Figure S2:** True skill statistics (TSS) values of the three sets of SDMs calibrated with occurrence data from the native range, introduced or non‐native range in Europe, and the combination of native and non‐native range (All). Boxplot center, hinges, and whiskers denote median, upper/lower quartiles, and upper/lower deciles, respectively.
**Figure S3:** Range of probability values for the best‐suited native and NNTs under historic climate and two climate change scenarios, SSP2‐4.5 and SSP5‐8.5, for the time frame 2061–2080. Boxplot centre, hinges and whiskers denote median, upper/lower quartiles and upper/lower deciles, respectively.
**Figure S4:** Range shift maps of (A) coniferous NNTs and (B) Broadleaved NNTs. Range shift (overlap, contraction, and expansion) refers to changes in potential species range between the historic period (1961–1990) and the future time frame 2061–2080 under two climate change scenarios, SSP2‐4.5 and SSP5‐8.5.
**Figure S5:** Potential climate suitability depicting forested grid cells with native and NNT species predicted to have the highest climatic suitability in the historic climate and two future time frames under SSP2‐4.5 and SSP5‐8.5 scenarios.
**Figure S6:** Comparison of range shift between widely occurring native conifer *Picea abies* and a potential alternative NNT, *Pseudotsuga menziesii* .
**Figure S7:** Top 5 native and NNT species within each bioclimatic region of Europe under historic (1961–1990) and SSP2 and SSP5 scenarios in 2061–2080.
**Figure S8:** Relation between potential range shifts (contraction, overlap, and expansion) and geographic spread of the occurrence data represented by the number of countries covered by the occurrence data.
**Figure S9:** Uncertainty expressed in standard deviation of probability ranging from 0 to 1 in the predicted potential distribution of *Acacea delabata* under historic and future scenarios. The analysis presented in this paper is based only on Historic and SSP2‐4.5 2061–2080, and SSP5‐8.5 2061–2080.
**Figure S10:** Uncertainty expressed in standard deviation of probability ranging from 0 to 1 in the predicted potential distribution of *Abies grandis* under historic and future scenarios. The analysis presented in this paper is based only on Historic and SSP2‐4.5 2061–2080, and SSP5‐8.5 2061–2080.
**Figure S11:** Uncertainty expressed in standard deviation of probability ranging from 0 to 1 in the predicted potential distribution of *Acer negundo* under historic and future scenarios. The analysis presented in this paper is based only on Historic and SSP2‐4.5 2061–2080, and SSP5‐8.5 2061–2080.
**Figure S12:** Uncertainty expressed in standard deviation of probability ranging from 0 to 1 in the predicted potential distribution of *Fraxinus pennsylvanica* under historic and future scenarios. The analysis presented in this paper is based only on Historic and SSP2‐4.5 2061–2080, and SSP5‐8.5 2061–2080.
**Figure S13:** Uncertainty expressed in standard deviation of probability ranging from 0 to 1 in the predicted potential distribution of *Juglans nigra* under historic and future scenarios. The analysis presented in this paper is based only on Historic and SSP2‐4.5 2061–2080, and SSP5‐8.5 2061–2080.
**Figure S14:** Uncertainty expressed in standard deviation of probability ranging from 0 to 1 in the predicted potential distribution of *Pinus contorta* under historic and future scenarios. The analysis presented in this paper is based only on Historic and SSP2‐4.5 2061–2080, and SSP5‐8.5 2061–2080.
**Figure S15:** Uncertainty expressed in standard deviation of probability ranging from 0 to 1 in the predicted potential distribution of *Prunus serotina* under historic and future scenarios. The analysis presented in this paper is based only on Historic and SSP2‐4.5 2061–2080, and SSP5‐8.5 2061–2080.
**Figure S16:** Uncertainty expressed in standard deviation of probability ranging from 0 to 1 in the predicted potential distribution of *Pseudotsuga menziesii* under historic and future scenarios. The analysis presented in this paper is based only on Historic and SSP2‐4.5 2061–2080, and SSP5‐8.5 2061–2080.
**Figure S17:** Uncertainty expressed in standard deviation of probability ranging from 0 to 1 in the predicted potential distribution of *Picea pungens* under historic and future scenarios. The analysis presented in this paper is based only on Historic and SSP2‐4.5 2061–2080, and SSP5‐8.5 2061–2080.
**Figure S18:** Uncertainty expressed in standard deviation of probability ranging from 0 to 1 in the predicted potential distribution of *Pinus radiata* under historic and future scenarios. The analysis presented in this paper is based only on Historic and SSP2‐4.5 2061–2080, and SSP5‐8.5 2061–2080.
**Figure S19:** Uncertainty expressed in standard deviation of probability ranging from 0 to 1 in the predicted potential distribution of *Picea sitchensis* under historic and future scenarios. The analysis presented in this paper is based only on Historic and SSP2‐4.5 2061–2080, and SSP5‐8.5 2061–2080.
**Figure S20:** Uncertainty expressed in standard deviation of probability ranging from 0 to 1 in the predicted potential distribution of *Pinus strobus* under historic and future scenarios. The analysis presented in this paper is based only on Historic and SSP2‐4.5 2061–2080, and SSP5‐8.5 2061–2080.
**Figure S21:** Uncertainty expressed in standard deviation of probability ranging from 0 to 1 in the predicted potential distribution of *Quercus rubra* under historic and future scenarios. The analysis presented in this paper is based only on Historic and SSP2‐4.5 2061–2080, and SSP5‐8.5 2061–2080.
**Figure S22:** Uncertainty expressed in standard deviation of probability ranging from 0 to 1 in the predicted potential distribution of *Robinia pseudoacacia* under historic and future scenarios. The analysis presented in this paper is based only on Historic and SSP2‐4.5 2061–2080, and SSP5‐8.5 2061–2080.
**Figure S23:** Uncertainty expressed in standard deviation of probability ranging from 0 to 1 in the predicted potential distribution of *Thuja plicata* under historic and future scenarios. The analysis presented in this paper is based only on Historic and SSP2‐4.5 2061–2080, and SSP5‐8.5 2061–2080.

**Table S12:** Summary of the climatic suitability of native and NNT species within each bioclimatic region of Europe. (Given as a separate csv file).

## Data Availability

The paper uses a publicly available dataset on the potential distribution of non‐native and native tree species and climate, as mentioned below. Dataset on the potential distribution of native species: Chakraborty et al. (2024) and Chakraborty, Móricz, et al. (2021). Dataset on the potential distribution of NNTs: Chakraborty et al. (2025). Dataset on the occurrence of NNTs: The majority of the occurrence data for NNT species are from the publicly available EU‐Forest dataset (Mauri et al. 2017). This dataset was extended by including National Forest Inventory data from several other countries, including the Balkan countries, listed in Table S2. This data was obtained by EU‐COST Action NNEXT and cannot be shared without a formal data sharing agreement with the individual countries. Climate Data: Chakraborty, Dobor, et al. (2021) and Fick and Hijmans (2017).
