# Supplementary material for: Climate‐Driven Range Shifts Limit the Role of Non‐Native Trees for Adaptation Processes of European Forests
Source: Ecol Evol. 2026 Jul 8;16(7):e73999. doi: 10.1002/ece3.73999 (PMC13345991; doi:10.1002/ece3.73999)
Supplement: Supplementary file 1 — Table S1: Non‐native tree species (NNTs) and Native tree species were included in the study. NNTs with * represent those used to compare the climate suitability of native and NNTs to identify potential alternatives for forest management under climate change. Table S2: Sources of occurrence data for NNTs collected by EU Cost Action NNEXT. The COST Action NNEXT compiled occurrence (coordinates in latitude and longitude) of the target NNTs from various sources described below. Table S3: Number of presence and pseudoabsences used to calibrate the SDMs for NNTs with occurrence data from (i) native range of the species, (ii) introduced range in Europe, (iii) combined dataset with both native and introduced range occurrences. Table S4: The number of presence and absence records used to calibrate the SDMs for the native species. Table S5: The subset of bioclimatic climate variables from the Worldclim2.0 dataset used as predictor variables for developing the SDMs for NNTs. Table S6: Bioclimatic variables from the ECLIPS.20 dataset retained after variable selection to be used to calibrate the SDMs for the respective native species. Table S7: Statistics for evaluation of the modeling algorithms of biomd2 used to develop the ensemble SDM for the NNTs. In the analysis models, calibrated with all (i.e., occurrence data from native & introduced range) of the respective NNTs were used. Table S8: Statistics for evaluation of the ensemble SDM for the target NNTs calibrated with native, non‐native, and all (native + introduced range of the restive NNTs) occurrence data. Table S9: Statistics for evaluation for each of the models used to develop the ensemble SDM for the native species. Table S10: Statistics for evaluation of the ensemble SDM for the 13 native tree species. Table S11: Cut‐off values used by biomod2 to convert probabilities (0–1) to binary values of presence = 1 and absence = 0 by maximizing TSS. These cut‐off values of the ensemble models were only used to convert the pr [file ECE3-16-e73999-s002.docx]

**Supporting Information**

**Climate-driven range shifts limit the role of non-native trees for adaptation processes of European forests**

Contents

[**Description of the SDMs for Native and NNT species** 3](#_Toc225844604)

[**Table S1.** Non-native tree species (NNTs) and Native tree species were included in the study. NNTs with * represent those used to compare the climate suitability of native and NNTs to identify potential alternatives for forest management under climate change 5](#_Toc225844605)

[**Table S2.** Sources of occurrence data for NNTs collected by EU Cost Action NNEXT. The COST Action NNEXT compiled occurrence (coordinates in latitude and longitude) of the target NNTs from various sources described below. 6](#_Toc225844606)

[**Table S3:** Number of presence and pseudoabsences used to calibrate the SDMs for NNTs with occurrence data from i) native range of the species, ii) introduced range in Europe, ii) combined dataset with both native and introduced range occurrences 10](#_Toc225844607)

[**Table S4:** The number of presence and absence records used to calibrate the SDMs for the native species 11](#_Toc225844608)

[**Table S5:** The subset of bioclimatic climate variables from the Worldclim2.0 dataset used as predictor variables for developing the SDMs for NNTs. 12](#_Toc225844609)

[**Table S6:** Bioclimatic variables from the ECLIPS.20 dataset retained after variable selection to be used to calibrate the SDMs for the respective native species. 14](#_Toc225844610)

[**Table S7:** Statistics for evaluation of the modelling algorithms of biomd2 used to develop the ensemble SDM for the NNTs. In the analysis models, calibrated with all (i.e., occurrence data from native & introduced range) of the respective NNTs were used 19](#_Toc225844611)

[**Table S8:** Statistics for evaluation of the ensemble SDM for the target NNTs calibrated with native, non-native, and all (native + introduced range of the restive NNTs) occurrence data. 29](#_Toc225844612)

[**Table S9:** Statistics for evaluation for each of the models used to develop the ensemble SDM for the native species 31](#_Toc225844613)

[**Table S10**: Statistics for evaluation of the ensemble SDM for the 13 native tree species. 34](#_Toc225844614)

[**Table S11.** Cut-off values used by biomod2 to convert probabilities (0-1) to binary values of presence= 1 and absence= 0 by maximizing TSS. These cut-off values of the ensemble models were only used to convert the predicted probability of occurrence of NNTs to estimate their range shifts in each 1x1 km forested grid cell of Europe. 34](#_Toc225844615)

[**Table S12**: Summary of the climatic suitability of native and NNT species within each bioclimatic region of Europe. (Given as a separate csv file). 35](#_Toc225844616)

[**Fig. S1** Distribution of the presence locations of the 15 NNT species. Red points indicate the observed native distribution of the respective species, while blue points indicate the observed presence locations in their introduced range in Europe. 36](#_Toc225844617)

[**Fig. S2** True skill statistics (TSS) values of the three sets of SDMs calibrated with occurrence data from the native range, introduced or non-native range in Europe, and the combination of native and non-native range (All). Boxplot center, hinges, and whiskers denote median, upper/lower quartiles, and upper/lower deciles, respectively. 37](#_Toc225844618)

[**Fig. S3** Range of probability values for the best-suited native and NNTs under historic climate and two climate change scenarios, SSP2-4.5 and SSP5-8.5, for the time frame 2061-80. Boxplot centre, hinges and whiskers denote median, upper/lower quartiles and upper/lower deciles, respectively. 38](#_Toc225844619)

[**Fig. S4** Range shift maps of (A) coniferous NNTs and (B) Broadleaved NNTs. Range shift (overlap, contraction, and expansion) refers to changes in potential species range between the historic period (1961-90) and the future time frame 2061-80 under two climate change scenarios, SSP2-4.5 and SSP5-8.5. 39](#_Toc225844620)

[**Fig. S5** Potential climate suitability depicting forested grid cells with native and NNT species predicted to have the highest climatic suitability in the historic climate and two future time frames under SSP2-4.5 and SSP5-8.5 scenarios. 40](#_Toc225844621)

[**Fig. S6.** Comparison of range shift between widely occurring native conifer *Picea abies* and a potential alternative NNT, *Pseudotsuga menziesii* 41](#_Toc225844622)

[**Fig. S7** Top 5 native and NNT species within each bioclimatic region of Europe under historic (1961-90) and SSP2 and SSP5 scenarios in 2061-80. 42](#_Toc225844623)

[**Fig. S8** Relation between potential range shifts (contraction, overlap, and expansion) and geographic spread of the occurrence data represented by the number of countries covered by the occurrence data. 43](#_Toc225844624)

[**Fig. S9** Uncertainty expressed in standard deviation of probability ranging from 0 to1 in the predicted potential distribution of *Acacea delabata* under historic and future scenarios. The analysis presented in this paper is based only on Historic and SSP2-4.5 2061-80, and SSP5-8.5 2061-80. 44](#_Toc225844625)

[**Fig. S10** Uncertainty expressed in standard deviation of probability ranging from 0 to1 in the predicted potential distribution of Abies grandis under historic and future scenarios. The analysis presented in this paper is based only on Historic and SSP2-4.5 2061-80, and SSP5-8.5 2061-80. 45](#_Toc225844626)

[**Fig. S11** Uncertainty expressed in standard deviation of probability ranging from 0 to1 in the predicted potential distribution of *Acer negundo* under historic and future scenarios. The analysis presented in this paper is based only on Historic and SSP2-4.5 2061-80, and SSP5-8.5 2061-80. 46](#_Toc225844627)

[**Fig. S12** Uncertainty expressed in standard deviation of probability ranging from 0 to1 in the predicted potential distribution of Fraxinus pennsylvanica under historic and future scenarios. The analysis presented in this paper is based only on Historic and SSP2-4.5 2061-80, and SSP5-8.5 2061-80. 47](#_Toc225844628)

[**Fig. S13** Uncertainty expressed in standard deviation of probability ranging from 0 to1 in the predicted potential distribution of *Juglans nigra* under historic and future scenarios. The analysis presented in this paper is based only on Historic and SSP2-4.5 2061-80, and SSP5-8.5 2061-80. 48](#_Toc225844629)

[**Fig. S14** Uncertainty expressed in standard deviation of probability ranging from 0 to1 in the predicted potential distribution of *Pinus contorta* under historic and future scenarios. The analysis presented in this paper is based only on Historic and SSP2-4.5 2061-80, and SSP5-8.5 2061-80. 49](#_Toc225844630)

[**Fig. S15** Uncertainty expressed in standard deviation of probability ranging from 0 to1 in the predicted potential distribution of *Prunus serotina* under historic and future scenarios. The analysis presented in this paper is based only on Historic and SSP2-4.5 2061-80, and SSP5-8.5 2061-80. 50](#_Toc225844631)

[**Fig. S16** Uncertainty expressed in standard deviation of probability ranging from 0 to1 in the predicted potential distribution of *Pseudotsuga menziesii* under historic and future scenarios. The analysis presented in this paper is based only on Historic and SSP2-4.5 2061-80, and SSP5-8.5 2061-80. 51](#_Toc225844632)

[**Fig. S17** Uncertainty expressed in standard deviation of probability ranging from 0 to1 in the predicted potential distribution of *Picea pungens* under historic and future scenarios. The analysis presented in this paper is based only on Historic and SSP2-4.5 2061-80, and SSP5-8.5 2061-80. 51](#_Toc225844633)

[**Fig. S18** Uncertainty expressed in standard deviation of probability ranging from 0 to1 in the predicted potential distribution of *Pinus radiata* under historic and future scenarios. The analysis presented in this paper is based only on Historic and SSP2-4.5 2061-80, and SSP5-8.5 2061-80. 52](#_Toc225844634)

[**Fig. S19** Uncertainty expressed in standard deviation of probability ranging from 0 to1 in the predicted potential distribution of *Picea sitchensis* under historic and future scenarios. The analysis presented in this paper is based only on Historic and SSP2-4.5 2061-80, and SSP5-8.5 2061-80. 53](#_Toc225844635)

[**Fig. S20** Uncertainty expressed in standard deviation of probability ranging from 0 to1 in the predicted potential distribution of *Pinus strobus* under historic and future scenarios. The analysis presented in this paper is based only on Historic and SSP2-4.5 2061-80, and SSP5-8.5 2061-80. 53](#_Toc225844636)

[**Fig. S21** Uncertainty expressed in standard deviation of probability ranging from 0 to1 in the predicted potential distribution of *Quercus rubra* under historic and future scenarios. The analysis presented in this paper is based only on Historic and SSP2-4.5 2061-80, and SSP5-8.5 2061-80. 54](#_Toc225844637)

[**Fig. S22** Uncertainty expressed in standard deviation of probability ranging from 0 to1 in the predicted potential distribution of Robinia pseudoacacia under historic and future scenarios. The analysis presented in this paper is based only on Historic and SSP2-4.5 2061-80, and SSP5-8.5 2061-80. 55](#_Toc225844638)

[**Fig. S23** Uncertainty expressed in standard deviation of probability ranging from 0 to1 in the predicted potential distribution of *Thuja plicata* under historic and future scenarios. The analysis presented in this paper is based only on Historic and SSP2-4.5 2061-80, and SSP5-8.5 2061-80. 56](#_Toc225844639)

[**References** 57](#_Toc225844640)

### **Description of the SDMs for Native and NNT species**

Potential distribution of the 13 widely occurring native species (Chakraborty et al. 2024a, Chakraborty etal 2021b) and 15 NNTs (Chakraborty etal 2024b) (Table S1) were predicted with ensemble species distribution models (SDMs) calibrated with observed occurrence locations (presence-absence) as dependent variables and bioclimatic variables as independent variables, using the R package biomod2 (Thuiller W et al., 2016). The biomod2 package allowes to model the potential distribution of species with 10 different modelling algorithms and also allows for ensembling the models. The predicted maps of potential distribution depict the climatically suitable potential distribution of the target species as probability of occurrence (0-1) at a spatial resolution of 30-arc sec, which is roughly equivalent to 1km depending on latitude.

The model workflow includes:

1. Occurrence data, data cleaning, and presence-pseudoabsence generation
2. Variable selection
3. Model calibration
4. Model ensembling
5. Model prediction
6. Model evaluation and uncertainty analysis

#### **Occurrence data**

The occurrence data for the 15 NNTs in their native and introduced ranges in Europe were obtained from various sources, including published harmonised records from National Forest Inventories (Mauri et al., 2017), the Global Biodiversity Facility, and data collected by the EU COST Action [NNEXT](https://www.cost.eu/actions/FP1403/) (Table S2). This dataset comprises records of observed occurrences of the target NNT in its native as well as introduced range in Europe (Fig. S1 in Supplementary Information). The occurrence data from Mauri et al., 2017 were at a 1km resolution wheras the precision of occurrence locations from other sources mentioned in the Table S2 were grouped into tiles, with “coordinate uncertainty in meters” values into ‘precision < 1km ’, ‘precision < 5km’, or ‘precision <10km’. Data with a precision of < 1km was only included in the analysis.

Occurrence data were used to develop the SDMs for the native species Chakraborty et al., 2024a; Chakraborty et al., 2021b) were from Mauri et al., (2017). This dataset harmonizes European tree occurrence from National Forest inventories (NFI), Forest Focus, and Biosoil datasets (Heiderer et al. 2011). The occurrence data for both NNTs and native species were harmonized and aggregated to a spatial resolution of 30 arc-sec.

### **Table S1.** Non-native tree species (NNTs) and Native tree species were included in the study. NNTs with * represent those used to compare the climate suitability of native and NNTs to identify potential alternatives for forest management under climate change

| Species | **Country/region of native occurrence** |
| --- | --- |
| *Abies grandis** | Northwestern North America (Pacific Northwest, USA and Canada) |
| *Acacia dealbata* | Southeastern Australia, Tasmania, |
| *Acer negundo* | Eastern and Central North America |
| *Fraxinus pennsylvanica** | Eastern and Central North America |
| *Juglans nigra** | Eastern United States |
| *Picea pungens** | Central and Southern Rocky Mountains, USA |
| *Picea sitchensis** | Coastal Alaska to Northern California |
| *Pinus contorta** | Western North America (Alaska to California and Colorado) |
| *Pinus radiata** | California and Mexico |
| *Pinus strobus** | Eastern North America |
| *Prunus serotina* | Eastern North America, parts of Mexico, and Central America |
| *Pseudotsuga menziesii** | Western North America (Pacific Northwest, Rocky Mountains) |
| *Quercus rubra** | Eastern and Central North America |
| *Robinia pseudoacacia** | Southeastern United States (Appalachians, Ozarks). |
| *Thuja plicata** | Northwestern North America (Alaska to Northern California) |
| *Abies alba* | Mountainous and sub-mountainous regions of central and southern Europe |
| *Fagus sylvatica* | Temperate and montane forests across central and southern Europe |
| *Larix decidua* | Alpine and Carpathian Mountain zones |
| *Picea abies* | Boreal and montane forests of northern, central, and eastern Europe |
| *Pinus sylvestris* | Boreal, sub-boreal, and temperate regions across most of Europe |
| *Quercus petrea* | Upland and hilly regions of temperate Europe |
| *Quercus robur* | Lowland and riparian zones throughout temperate Europe |
| *Pinus halepensis* | Mediterranean coastal and lowland zones |
| *Pinus nigra* | Mediterranean and sub-Mediterranean mountainous regions |
| *Pinus pinea* | Mediterranean lowlands and coastal plains |
| *Quercus ilex* | Mediterranean evergreen woodlands and coastal areas |
| *Quercus pubescens* | Dry, sub-Mediterranean, and continental hill and foothill regions |
| *Quercus suber* | The Western Mediterranean Basin spanning southwestern Europe and northwestern Africa. |

### **Table S2.** Sources of occurrence data for NNTs collected by EU Cost Action [NNEXT.](https://www.cost.eu/actions/FP1403/) The COST Action NNEXT compiled occurrence (coordinates in latitude and longitude) of the target NNTs from various sources described below.

**EU-Forest.** Mauri et al. (2017) published a harmonized dataset containing NFI data from 21 European Countries, the Forest Focus and Biosoil dataset on a 1 km × 1 km grid. Data for the considered species were extracted, and coordinates were converted to WGS84. Mauri, A., Strona, G. & San-Miguel-Ayanz, J. EU-Forest, a high-resolution tree occurrence dataset for Europe. *Sci Data* **4**, 160123 (2017). https://doi.org/10.1038/sdata.2016.123

**GBIF- The Global Biodiversity Information Facility (GBIF).** This dataset contains global distribution data consisting of thousands of heterogeneous datasets. Data points with information on known issues were removed, and points with “*coordinateUncertaintyInMeters*” >1km were removed.

**NFI Montenegro**. Data from the NFI of Montenegro was searched for the listed species and joined with the provided precise coordinates of the inventory plots.

**BOKU Dataset**. An additional forest inventory dataset for *Pseudotsuga menziesii* for Austria and Germany, collected at BOKU Vienna, was provided by Elisabeth Pötzelsberger (née Pötzelsberger). The data is for Austria and Germany with highly precise coordinates.

**Balkan Vegetation Database & Balkan Dry Grassland Database**. The Balkan Vegetation Database and the Balkan Dry Grassland Database contain vegetation data from the whole Balkan region. Data was provided by Kiril Vasilev from EU-COST ACTION NNEXT and contains precise coordinate data mainly for Bulgaria.

**FIADB**. The Forest Inventory and Analysis National Program is the national forest inventory of the United States. Detailed inventory methods can vary from state to state, but it provides a forest dataset with high spatial resolution (1x1km). Data was downloaded from the online data-mart (http://apps.fs.fed.us/fiadb-downloads/datamart.html) for the whole United States. Plots with the presence of the relevant species were identified and linked with the coordinates provided also by the data mart.

**VegBank.** The database was searched for georeferenced occurrence data of the non-native tree species at a 1km resolution

**VRI - British Columbia, Canada**. The Forest Vegetation Composite Polygons Layer was provided by the “Ministry of Forests, Lands and Natural Resource Operations” of British Columbia, Canada. The polygons contain information on the most common tree species in the denoted polygons. Coordinates of the center of polygons containing information on the studied species were extracted.

**ESIS –Alberta, Canada.** The “Ecological Site Information database’ by the Alberta Sustainable Resource Development consists of site plots with a precision of 1 km. The dataset was provided by the “Department for Environment and Parks of Alberta”. It was searched for the non-native tree species and linked with the coordinates for the plots.

**InfoFlora.** Swiss national Vegetation Database with many different data providers. Especially, data on Robinia pseudoacacia and Ailanthus altissima is very numerous, because of their status as invasive species.

**Personal Dataset of Joana Vicente.** A personal Dataset by Joana Vicente compiled for previous studies, consisting of occurrence data of non-native trees in Europe, was included.

**Canada NFI**. Data from the National Forest Inventory of Canada. Plots are assigned to our ~1km grid cells.

**iNaturalist**. Data from https://www.inaturalist.org/

#### **Bioclimatic variables**

The bioclimatic variables to calibrate the SDMs for NNTs included 19 biologically relevant climate variables, comprising annual, seasonal, and monthly variables from Worldclim2.0 (Fick and Hijmans 2017) at a spatial resolution of 30-arc sec. These variables represent annual trends, seasonality, and extreme or limiting environmental factors derived from monthly temperature and precipitation data. Temperature variables (BIO1–BIO11) are expressed in degrees Celsius (°C), except for BIO4, which represents temperature seasonality (standard deviation ×100). Precipitation variables (BIO12–BIO19) are expressed in millimeters (mm), except for BIO15, which represents precipitation seasonality (coefficient of variation, %). Isothermality (BIO3) is expressed as a percentage shown below

BIO1: Annual Mean Temperature (°C)

BIO2: Mean Diurnal Range (°C)

BIO3: Isothermality (%)

BIO4: Temperature Seasonality (°C ×100)

BIO5: Maximum Temperature of Warmest Month (°C)

BIO6: Minimum Temperature of Coldest Month (°C)

BIO7: Temperature Annual Range (°C)

BIO8: Mean Temperature of Wettest Quarter (°C)

BIO9: Mean Temperature of Driest Quarter (°C)

BIO10: Mean Temperature of Warmest Quarter (°C)

BIO11: Mean Temperature of Coldest Quarter (°C)

BIO12: Annual Precipitation (mm)

BIO13: Precipitation of Wettest Month (mm)

BIO14: Precipitation of Driest Month (mm)

BIO15: Precipitation Seasonality (%)

BIO16: Precipitation of Wettest Quarter (mm)

BIO17: Precipitation of Driest Quarter (mm)

BIO18: Precipitation of Warmest Quarter (mm)

BIO19: Precipitation of Coldest Quarter (mm)

To calibrate the SDMs for native species, bioclimatic variables from the ECLIPS 2.0 (Chakraborty et al. 2021a) dataset at a 30 arc sec resolution were used. These variables include:

AHM annual heat:moisture index (MAT+10)/(MAP/1000))

bFFP the Julian date on which FFP begins

DDabove18 degree-days below 18°C, heating degree-days

DDabove5 degree-days above 5°C, growing degree-days

DDbelow0 degree-days below 0°C, chilling degree-days

DDbelow18 degree-days below 18°C, heating degree-days

eFFP the Julian date on which FFP ends

EMT Extreme minimum temperature over 30 years

FFP frost-free period

MAP mean annual precipitation (mm)

MAT mean annual temperature (°C)

MCMT Mean coldest month temperature (°C)

MSP Mean summer (May to Sept.) precipitation (mm)

MWMT mean warmest month temperature (°C)

NFFD the number of frost-free days

PPT_at autumn precipitation (mm)

PPT_sm summer precipitation (mm)

PPT_sp spring precipitation (mm)

PPT_wt winter precipitation (mm)

PPT01 to 12 Precipitation month 01(Jan) to 12 (Dec)

SHM summer heat: moisture index ((MWMT)/(MSP/1000))

Tave_at autumn (Sep. - Nov.) mean temperature (°C)

Tave_sm summer (Jun. - Aug.) mean temperature (°C)

Tave_sp spring (Mar. - May) mean temperature (°C)

Tave_wt winter (Dec.(prev. yr) - Feb.) mean temperature (°C)

Tave01 to 12 Average temperature month 01(Jan) to 12 (Dec)

TD Temperature difference between MWMT and MCMT(°C)

Tmax_an Maximum yearly temperature

Tmax_at Maximum autumn temperature

Tmax_sm Maximum summer temperature

Tmax_sp Maximum spring temperature

Tmax_wt Maximum winter temperature

Tmax 01 to 12 Maximum temperature 01(Jan) to 12 (Dec)

Tmin_an Minimum annual temperature

Tmin_at Minimum autumn temperature

Tmin_sm Minimum summer temperature

Tmin_sp Minimum spring temperature

Tmin_wt Minimum winter temperature

Tmin01 to 12 Minimum temperature 01(Jan) to 12 (Dec)

Bioclimatic variables for future climate for NNT species were from 13 GCM projections available in the Worldclim2.0 dataset. These GCMS are ACCESS-CM2, BCC-CSM2-MR, CMCC-ESM2, EC-Earth3-Veg, FIO-ESM-2-0, GISS-E2-1-G, HadGEM3-GC31-LL, INM-CM5-0, IPSL-CM6A-LR, MIROC6, MPI-ESM1-2-HR, MRI-ESM2-0, UKESM1-0-LL. For native spcies the future bioclimatic variables were from 5 GCM projections available in the ECLIPS.2.0 dataset, such as: CNRM-CERFACS-CNRM-CM5, MPI-M-MPI-ESM-LR, ICHEC-EC-EARTH, MOHC-HadGEM2-ES, MPI-M-MPI-ESM-LR.

The bioclimatic variables used to develop the SDMs for both native species and NNTs were for historical climate (1961-1990) and two Shared Socioeconomic Pathways scenarios used in IPCC’s Sixth Assessment Report, SSP2-4.5 and SSP5-8.5, for the period 2041-2060, 2061-2080, and 2081-2100 (IPCC 2021; Riahi et al., 2017).

#### **Treatment of occurrence data**

The occurrence dataset for NNTs included a total of 10,92,690 occurrence records of the target species (i.e., presence locations) in their natural range as well as introduced range in Europe. For each NNT, 10,000 background points were randomly selected, which represented the pseudo-absence locations using a 30-arcsec raster corresponding to the spatial resolution of the bioclimatic variables of Worldclim2.0. following the approach described in (Barbet-Massin et al. 2012). This dataset, containing presence and pseudoabsence, was further thinned to reduce spatial autocorrelation and account for sampling biases. At first, all the duplicates of the same tree species falling in each 30 arc-sec (~1 km) cell were first removed. Secondly, only one record was retained randomly every 10x 10 km grid cell. With this approach, three sets of occurrence data were generated: i) presence and pseudoabsence locations from natural or native range; ii) presence and pseudoabsence locations from the introduced range of the target species in Europe; and iii) presence and pseudoabsence locations from the combined dataset, including both the natural and introduced range of the target species. These datasets were used to calibrate three sets of SDMs for each target species.

After thinning, 987,904 presence records and 126,381 pseudoabsences were retained for calibrating the SDMs for NNTs with biomod2 described below (Table S3).

### **Table S3:** Number of presence and pseudoabsences used to calibrate the SDMs for NNTs with occurrence data from i) native range of the species, ii) introduced range in Europe, ii) combined dataset with both native and introduced range occurrences

|  | **Presence locations** | | | **Pseudoabsence** |
| --- | --- | --- | --- | --- |
| **Species** | **Native range** | **Introduced range** | **Total presences** |  |
| *Abies grandis* | 74,852 | 1,691 | 76,543 | 8,500 |
| *Acacia dealbata* | 4,907 | 3,230 | 8,137 | 7,600 |
| *Acer negundo* | 67,491 | 9,420 | 76,911 | 9,100 |
| *Fraxinus pennsylvanica* | 155,693 | 535 | 156,228 | 9,500 |
| *Juglans nigra* | 55,627 | 939 | 56,566 | 6,900 |
| *Picea pungens* | 3,964 | 852 | 4,816 | 9,500 |
| *Picea sitchensis* | 66,916 | 15,738 | 82,654 | 9,016 |
| *Pinus contorta* | 312,066 | 4,743 | 316,809 | 5,800 |
| *Pinus radiata* | 5,858 | 5,731 | 11,589 | 5,900 |
| *Pinus strobus* | 1,254 | 3,076 | 4,330 | 9,513 |
| *Prunus serotina* | 546 | 59,200 | 59,746 | 7,952 |
| *Pseudotsuga menziesii* | 907 | 23,717 | 24,624 | 8,700 |
| *Quercus rubra* | 916 | 35,133 | 36,049 | 8,800 |
| *Robinia pseudoacacia* | 459 | 71,351 | 71,810 | 9,700 |
| *Thuja plicata* | 321 | 771 | 1,092 | 9,900 |
|  |  |  | 987,904 | 126,381 |

The sample size of the occurrence data for native species included 588,983 occurrences (presence locations) at a spatial resolution of 1x1km (Mauri et al. 2017). The geographic locations of the target species in the EU-Forest dataset (Mauri et al. 2017) were assumed to be true presences. Conversely, the remaining occurrences of all other species, including the remaining target species, were treated as true-absence locations.

To reduce spatial autocorrelation, the entire presence-absence data of the native species was thinned in the geographic and environmental space. To do so, the presence-absence locations were initially thinned, retaining only one occurrence (either presence or absence) within each cell 10 km apart. To ensure that absence locations are climatically distant from the observed presence locations, we utilized the presence data of each target species to generate a rectilinear Surface Range Envelope (SRE) considering all bioclimatic predictor variables from the ECLIPS.20 dataset (Chakraborty et al.2021). When running the SRE model, the top and bottom 2.5% of the most extreme values for each variable along the gradient were excluded (Thuiller et al. 2016). Subsequently, all absence data overlapping with the predicted surface range envelope were removed, while the remaining absence dataset was used for model calibration. After thinning, 513,422 occurrence records (presence and absence) of the native species were retained for calibrating the SDMs with biomod2 (Table S4).

### **Table S4:** The number of presence and absence records used to calibrate the SDMs for the native species

| **Species** | **Presence** | **Absence** | **Total** |
| --- | --- | --- | --- |
| *Abies alba* | 2,279 | 34,760 | 37,039 |
| *Fagus sylvatica* | 7,229 | 27,723 | 34,952 |
| *Larix decidua* | 2,657 | 34,244 | 36,901 |
| *Picea abies* | 13,062 | 19,131 | 32,193 |
| *Pinus sylvestris* | 14,703 | 17,638 | 32,341 |
| *Quercus petraea* | 4,653 | 31,518 | 36,171 |
| *Quercus robur* | 7,981 | 26,695 | 34,676 |
| *Pinus halepensis* | 13,120 | 45,188 | 58,308 |
| *Pinus nigra* | 10,652 | 33,268 | 43,920 |
| *Pinus pinea* | 4,373 | 38,696 | 43,069 |
| *Quercus ilex* | 22,661 | 26,018 | 48,679 |
| *Quercus pubescens* | 8,523 | 22,224 | 30,747 |
| *Quercus suber* | 5,975 | 38,452 | 44,427 |
| Total | 117,868 | 395,554 | 513,422 |

#### **Selection of bioclimatic variables for calibrating SDMs for native and NNT species**

For developing SDMs for NNT species, from the list of potential predictor bioclimatic variables from the Worldclim 2.0 dataset, the ones that explain most of the variation in the observed presence and absences of each species were selected with a recursive feature elimination approach (RFE) implemented within the Random Forest algorithm (Breiman 2001). Within the RFE approach, the variables were eliminated iteratively, starting from the full set of potential predictors (see environmental predictors above), and retaining only those variables that reduce the mean square error over random permutations of the same variable. The variables that were linearly correlated with other variables and had variance inflation factors VIF > 5 as suggested by Booth et al. (1994) were identified, and the ones with the lower value according to the Akaike Information Criteria (AIC) (Akaike 1974) were retained for further model development (Table S5).

### **Table S5:** The subset of bioclimatic climate variables from the Worldclim2.0 dataset used as predictor variables for developing the SDMs for NNTs.

| **Species** | **Variable** | **Rank** |
| --- | --- | --- |
| *A dealbata* | bio4 | 1 |
| *A dealbata* | bio12 | 2 |
| *A dealbata* | bio1 | 3 |
| *A dealbata* | bio10 | 4 |
| *A dealbata* | bio3 | 5 |
| *A grandis* | bio4 | 1 |
| *A grandis* | bio10 | 2 |
| *A grandis* | bio7 | 3 |
| *A grandis* | bio19 | 4 |
| *F pennsylvanica* | bio4 | 1 |
| *F pennsylvanica* | bio16 | 2 |
| *F pennsylvanica* | bio8 | 3 |
| *F pennsylvanica* | bio1 | 4 |
| *F pennsylvanica* | bio7 | 5 |
| *J nigra* | bio12 | 1 |
| *J nigra* | bio19 | 2 |
| *J nigra* | bio10 | 3 |
| *P contorta* | bio10 | 1 |
| *P contorta* | bio19 | 2 |
| *P contorta* | bio11 | 3 |
| *P contorta* | bio12 | 4 |
| *P menziesii* | bio4 | 1 |
| *P menziesii* | bio10 | 2 |
| *P menziesii* | bio3 | 3 |
| *P menziesii* | bio5 | 4 |
| *P pungens* | bio5 | 1 |
| *P pungens* | bio18 | 2 |
| *P pungens* | bio2 | 3 |
| *P pungens* | bio4 | 4 |
| *P pungens* | bio10 | 5 |
| *P radiata* | bio4 | 1 |
| *P radiata* | bio11 | 2 |
| *P radiata* | bio12 | 3 |
| *P radiata* | bio1 | 4 |
| *P serotina* | bio3 | 1 |
| *P serotina* | bio2 | 2 |
| *P serotina* | bio5 | 3 |
| *P serotina* | bio10 | 4 |
| *P sitchensis* | bio7 | 1 |
| *P sitchensis* | bio1 | 2 |
| *P sitchensis* | bio6 | 3 |
| *P sitchensis* | bio13 | 4 |
| *P strobus* | bio5 | 1 |
| *P strobus* | bio2 | 2 |
| *P strobus* | bio7 | 3 |
| *P strobus* | bio3 | 4 |
| *P strobus* | bio17 | 5 |
| *P strobus* | bio1 | 6 |
| *Q rubra* | bio4 | 1 |
| *Q rubra* | bio1 | 2 |
| *Q rubra* | bio10 | 3 |
| *Q rubra* | bio7 | 4 |
| *R pseudoacacia* | bio4 | 1 |
| *R pseudoacacia* | bio10 | 2 |
| *R pseudoacacia* | bio17 | 3 |
| *R pseudoacacia* | bio5 | 4 |
| *T plicata* | bio4 | 1 |
| *T plicata* | bio1 | 2 |
| *T plicata* | bio7 | 3 |
| *T plicata* | bio11 | 4 |
| *T plicata* | bio6 | 5 |

For the native species, from the list of potential biologically relevant predictor variables from the ECLIPS.20 dataset, those that explain most of the variation in the observed presence and absence of each species while accounting for multicollinearity were selected (Table S6). For this purpose, the covsel R -package (Häggström et al. 2015) was used for covariate selection, employing a two-step process: collinearity filtering (Step A) and model-specific embedding (Step B).

In Step A, less informative covariates among collinear pairs were iteratively removed based on univariate GLM p-values and the default correlation coefficient threshold of |r| < 0.70. In Step B, the covariates selected in Step A were used to fit models with embedded selection procedures, including GLM with elastic-net regularization, GAM with null-space penalization, and guided regularized RF. The retained covariates after regularization were ranked based on the absolute value of the regularized regression coefficients for GLM, chi-square statistic for GAM, and Mean Decrease Gini index for RF. An overall ranking was determined by the sum of the ranks for each covariate, resulting in the identification of the most important predictors. All other parameters related to the number of covariates and the maximum number of covariates to be selected were maintained at their default settings.

### **Table S6:** Bioclimatic variables from the ECLIPS.20 dataset retained after variable selection to be used to calibrate the SDMs for the respective native species.

| **Species** | **Variable** | **Rank** |
| --- | --- | --- |
| *Abies alba* | *SHM* | 1 |
| *Abies alba* | *Tmax_sm* | 2 |
| *Abies alba* | *TD* | 3 |
| *Abies alba* | *DDbelow0* | 4 |
| *Abies alba* | *PPT_sm* | 5 |
| *Abies alba* | *PPT_sp* | 6 |
| *Fagus sylvatica* | *bFFP* | 1 |
| *Fagus sylvatica* | *MAP* | 2 |
| *Fagus sylvatica* | *SHM* | 3 |
| *Fagus sylvatica* | *TD* | 4 |
| *Fagus sylvatica* | *PPT_sm* | 5 |
| *Larix decidua* | *DDbelow0* | 1 |
| *Larix decidua* | *SHM* | 2 |
| *Larix decidua* | *TD* | 3 |
| *Larix decidua* | *PPT_sp* | 4 |
| *Larix decidua* | *Tmax_sm* | 5 |
| *Larix decidua* | *PPT_sm* | 6 |
| *Picea abies* | *bFFP* | 1 |
| *Picea abies* | *AHM* | 2 |
| *Picea abies* | *PPT_sp* | 3 |
| *Picea abies* | *TD* | 4 |
| *Pinus sylvestris* | *bFFP* | 1 |
| *Pinus sylvestris* | *AHM* | 2 |
| *Pinus sylvestris* | *PPT_sm* | 3 |
| *Pinus sylvestris* | *PPT_sp* | 4 |
| *Quercus petraea* | *PPT_sm* | 1 |
| *Quercus petraea* | *DDbelow0* | 2 |
| *Quercus petraea* | *DDabove18* | 3 |
| *Quercus petraea* | *TD* | 4 |
| *Quercus petraea* | *PPT_sp* | 5 |
| *Quercus robur* | *bFFP* | 1 |
| *Quercus robur* | *PPT_sm* | 2 |
| *Quercus robur* | *TD* | 3 |
| *Quercus robur* | *AHM* | 4 |
| *Quercus robur* | *MAP* | 5 |
| *Pinus halepensis* | *SHM* | 1 |
| *Pinus halepensis* | *Tmax_sm* | 2 |
| *Pinus halepensis* | *PPT_sm* | 3 |
| *Pinus halepensis* | *DDbelow0* | 4 |
| *Pinus halepensis* | *PPT_sp* | 5 |
| *Pinus nigra* | *DDbelow0* | 1 |
| *Pinus nigra* | *SHM* | 2 |
| *Pinus nigra* | *TD* | 3 |
| *Pinus nigra* | *Tmax_sm* | 4 |
| *Pinus pinea* | *AHM* | 1 |
| *Pinus pinea* | *PPT_sm* | 2 |
| *Pinus pinea* | *PPT_sp* | 3 |
| *Quercus ilex* | *DDabove18* | 1 |
| *Quercus ilex* | *PPT_sm* | 2 |
| *Quercus ilex* | *TD* | 3 |
| *Quercus ilex* | *AHM* | 4 |
| *Quercus pubescens* | *TD* | 1 |
| *Quercus pubescens* | *Tmax_sm* | 2 |
| *Quercus pubescens* | *AHM* | 3 |
| *Quercus suber* | *Tmax_sm* | 1 |
| *Quercus suber* | *PPT_sm* | 2 |
| *Quercus suber* | *DDabove18* | 3 |

#### **Model calibration and ensembling with biomod2.0**

The ten modeling algorithms available in the biomod2 platform were used to calibrate SDMs for each target native and NNT species. These algorithms include GLM (Generalized Linear Models), GAM (Generalized Additive Models), GBM (Generalized Boosted regression Models), CTA (Classification Tree Analysis), ANN (Artificial Neural Networks), SRE (Surface Range Envelop or BIOCLIM), FDA (Flexible Discriminant Analysis), MARS (Multivariate Adaptive Regression Spline), RF (Random Forest for classification and regression), and MAXENT.

Apart from the ten modelling algorithms, the biomod2 also allows for ensemble SDM for each species. Those models with True Skill Statistics threshold (TSS > 0.7) were selected for the ensemble model. Thereafter, an ensemble model was developed for each Native and NNT species by calculating the median probability over the selected modelling algorithms in biomod2 with True Skill Statistics threshold (TSS > 0.7). TSS was preferred as an index for ensembling the models because it accounts for both omission and commission errors and is less sensitive to prevalence than earlier measures, such as Cohen’s Kappa, making it widely recommended for SDM evaluation (Allouche et al., 2006). The value TSS > 0.7 is commonly used in the SDM studies as a cut-off separating “reasonable or good” from “poor” model discrimination, favouring model algorithms with both relatively high sensitivity and specificity (Wang et al., 2024; Zhang et al., 2015) while avoiding excessively strict filtering that would discard most modelling algorithms.

Three sets of SDMs were calibrated for each NNT species, each with the observed occurrence and bioclimatic variables of the NNTs in their 1) native or natural range, 2) non-native or introduced range in Europe, and 3) combination of both native and non-native distribution range. Alien species often undergo ecological niche shifts when introduced to new environments. Relying solely on native range data may fail to capture these adaptations, leading to underestimations of potential distributions(Ørsted & Ørsted, 2019). Liu et al. (2022) found that models developed with combined native and introduced occurrences exhibited higher predictive performance, especially when accounting for niche changes and improving the transferability of SDMs across different geographic areas. (Davis et al., 2024) found that SDMs that utilize both native and non-native data can better inform risk assessments and management plans for alien species. Moreover, models that integrate data from both native and introduced ranges are better equipped to account for this environmental heterogeneity, leading to more accurate predictions of potential distributions and mitigating problems associated with sampling bias and data limitations. (Canelles et al., 2021; Steen et al., 2024). We also found that SDMs for NNTs in Europe, calculated with occurrence data from both native and introduced range in Europe, had significantly higher TSS compared to those calibrated with either native or introduced range occurrence data (Fig. S2 in Supplementary Information). Therefore, in this study, we used the potential distribution maps of NNTs from Chakraborty et al (2024b), calibrated with occurrence data from both native and non-native ranges.

#### **Projection of the SDMs under historic and future climates**

The 10 SDMs algorithms and one ensemble model from the biomod2.0 were projected to develop maps of potential distribution of each NNT and native species for the historical climate (1961-1990) and two Shared Socioeconomic Pathways scenarios used in IPCC’s Sixth Assessment Report, SSP2-4.5 and SSP5-8.5, for the period 2041-2060, 2061-2080, and 2081-2100 (IPCC 2021; Riahi et al., 2017). However, for comprehensive reporting, we restricted our analysis to historic (1961-90) and one future time frame, 2061-80 for SSP2-4.5 and SSP5-8.5 scenarios.

To account for uncertainty due to climate change scenarios, the ensemble models of the respective NNTs were projected for each of the 13 GCMs listed in the Worldclim2.0 dataset. The ensemble models for Native species were projected for 5 GCMs listed in the ECLIPS.20 dataset. In this analysis, we used the average predicted probability from the 13 ensemble projections for each NNT and from 5 ensemble projections for each native species for SSP2-4.5 and SSP5-8.5 scenarios for the period 2061-80.

Detailed information on the SDMs according to the ODMAP framework (Overview, Data, Model, Assessment, and Prediction)(Zurell et al., 2020) are available Chakraborty etal (2024b) and Chakraborty et al. (2024a) for the NNT and native species, respectively.

#### **Model evaluation and Uncertainty analysis**

Model evaluation was done by randomly splitting the occurrence dataset of each native species and NNTs into 75% for model calibration and 25% for model evaluation. Each such set is run 10 times to account for uncertainty with equal weight for presence and absence. For each such model run, as well as the final ensemble models for each target native and NNTs, the model evaluation statistics ROC & TSS, True Skill Statistics (Sensitivity+specificity-1) were recorded along with model sensitivity (the ability of the model to predict true presences), and model specificity (the ability of the model to predict the true absences) (Table S6, S7 S8, S9). The model ensemble was, however, based on TSS. TSS takes into account both omission and commission errors and ranges also from −1 to +1, not being affected by prevalence as KAPPA (Allouche et al. 2006). TSS values ranging from 0.2 to 0.5 were considered poor, from 0.6 to 0.8 useful, and values larger than 0.8 were good to excellent (e.g., Coetzee et al. 2009; Pontius et al. 2014).

Model uncertainty was calculated and mapped as standard deviation and standard error of predicted probability of occurrence from the resulting variation due to 10 model algorithms in the case of historical climate, 5 GCMs for native species, and 13 GCMs for NNTs in future climate.

### **Table S7:** Statistics for evaluation of the modelling algorithms of biomd2 used to develop the ensemble SDM for the NNTs. In the analysis models, calibrated with all (i.e., occurrence data from native & introduced range) of the respective NNTs were used

| *Species* | Sensitivity | Specificity | TSS | Model | Calibration Range |
| --- | --- | --- | --- | --- | --- |
| *Acacia dealbata* | 0.937 | 0.916 | 0.853 | GLM | Non-Native |
| *Acacia dealbata* | 0.931 | 0.916 | 0.847 | GAM | Non-Native |
| *Acacia dealbata* | 0.933 | 0.926 | 0.858 | GBM | Non-Native |
| *Acacia dealbata* | 0.928 | 0.921 | 0.850 | CTA | Non-Native |
| *Acacia dealbata* | 0.931 | 0.904 | 0.835 | ANN | Non-Native |
| *Acacia dealbata* | 0.939 | 0.929 | 0.868 | RF | Non-Native |
| *Acacia dealbata* | 0.939 | 0.923 | 0.862 | FDA | Non-Native |
| *Acacia dealbata* | 0.930 | 0.920 | 0.850 | MARS | Non-Native |
| *Acacia dealbata* | 0.933 | 0.913 | 0.846 | MAXENT | Non-Native |
| *Acacia dealbata* | 0.939 | 0.929 | 0.868 | GLM | Native |
| *Acacia dealbata* | 0.939 | 0.929 | 0.868 | GAM | Native |
| *Acacia dealbata* | 0.939 | 0.929 | 0.868 | GBM | Native |
| *Acacia dealbata* | 0.934 | 0.929 | 0.863 | CTA | Native |
| *Acacia dealbata* | 0.939 | 0.929 | 0.868 | ANN | Native |
| *Acacia dealbata* | 0.939 | 0.929 | 0.868 | RF | Native |
| *Acacia dealbata* | 0.939 | 0.929 | 0.868 | FDA | Native |
| *Acacia dealbata* | 0.939 | 0.929 | 0.868 | MARS | Native |
| *Acacia dealbata* | 0.939 | 0.929 | 0.868 | MAXENT | Native |
| *Acacia dealbata* | 0.979 | 0.969 | 0.948 | GLM | All |
| *Acacia dealbata* | 0.974 | 0.960 | 0.934 | GAM | All |
| *Acacia dealbata* | 0.967 | 0.965 | 0.932 | GBM | All |
| *Acacia dealbata* | 0.967 | 0.958 | 0.925 | CTA | All |
| *Acacia dealbata* | 0.960 | 0.938 | 0.899 | ANN | All |
| *Acacia dealbata* | 0.979 | 0.969 | 0.948 | RF | All |
| *Acacia dealbata* | 0.975 | 0.964 | 0.939 | FDA | All |
| *Acacia dealbata* | 0.974 | 0.951 | 0.925 | MARS | All |
| *Acacia dealbata* | 0.968 | 0.949 | 0.917 | MAXENT | All |
| *Abies grandis* | 0.927 | 0.916 | 0.843 | GLM | Non-Native |
| *Abies grandis* | 0.939 | 0.916 | 0.855 | GAM | Non-Native |
| *Abies grandis* | 0.929 | 0.927 | 0.856 | GBM | Non-Native |
| *Abies grandis* | 0.925 | 0.917 | 0.842 | CTA | Non-Native |
| *Abies grandis* | 0.922 | 0.909 | 0.831 | ANN | Non-Native |
| *Abies grandis* | 0.939 | 0.929 | 0.868 | RF | Non-Native |
| *Abies grandis* | 0.933 | 0.921 | 0.854 | FDA | Non-Native |
| *Abies grandis* | 0.923 | 0.920 | 0.843 | MARS | Non-Native |
| *Abies grandis* | 0.916 | 0.915 | 0.831 | Maxent | Non-Native |
| *Abies grandis* | 0.939 | 0.929 | 0.868 | GLM | Native |
| *Abies grandis* | 0.939 | 0.929 | 0.868 | GAM | Native |
| *Abies grandis* | 0.938 | 0.928 | 0.866 | GBM | Native |
| *Abies grandis* | 0.933 | 0.927 | 0.860 | CTA | Native |
| *Abies grandis* | 0.936 | 0.907 | 0.844 | ANN | Native |
| *Abies grandis* | 0.940 | 0.929 | 0.869 | RF | Native |
| *Abies grandis* | 0.939 | 0.924 | 0.863 | FDA | Native |
| *Abies grandis* | 0.934 | 0.926 | 0.860 | MARS | Native |
| *Abies grandis* | 0.935 | 0.924 | 0.859 | Maxent | Native |
| *Abies grandis* | 0.973 | 0.967 | 0.940 | GLM | All |
| *Abies grandis* | 0.971 | 0.956 | 0.927 | GAM | All |
| *Abies grandis* | 0.969 | 0.955 | 0.925 | GBM | All |
| *Abies grandis* | 0.967 | 0.956 | 0.923 | CTA | All |
| *Abies grandis* | 0.965 | 0.923 | 0.887 | ANN | All |
| *Abies grandis* | 0.979 | 0.969 | 0.948 | RF | All |
| *Abies grandis* | 0.971 | 0.959 | 0.930 | FDA | All |
| *Abies grandis* | 0.963 | 0.956 | 0.919 | MARS | All |
| *Abies grandis* | 0.960 | 0.941 | 0.901 | Maxent | All |
| *Acer negundo* | 0.925 | 0.913 | 0.838 | GLM | Non-Native |
| *Acer negundo* | 0.912 | 0.907 | 0.819 | GAM | Non-Native |
| *Acer negundo* | 0.916 | 0.917 | 0.834 | GBM | Non-Native |
| *Acer negundo* | 0.927 | 0.913 | 0.840 | CTA | Non-Native |
| *Acer negundo* | 0.913 | 0.843 | 0.756 | ANN | Non-Native |
| *Acer negundo* | 0.939 | 0.929 | 0.868 | RF | Non-Native |
| *Acer negundo* | 0.920 | 0.915 | 0.835 | FDA | Non-Native |
| *Acer negundo* | 0.924 | 0.911 | 0.835 | MARS | Non-Native |
| *Acer negundo* | 0.897 | 0.873 | 0.770 | MAXENT | Non-Native |
| *Acer negundo* | 0.937 | 0.928 | 0.866 | GLM | Native |
| *Acer negundo* | 0.933 | 0.926 | 0.858 | GAM | Native |
| *Acer negundo* | 0.935 | 0.928 | 0.863 | GBM | Native |
| *Acer negundo* | 0.933 | 0.924 | 0.857 | CTA | Native |
| *Acer negundo* | 0.907 | 0.905 | 0.812 | ANN | Native |
| *Acer negundo* | 0.939 | 0.929 | 0.868 | RF | Native |
| *Acer negundo* | 0.934 | 0.926 | 0.860 | FDA | Native |
| *Acer negundo* | 0.932 | 0.921 | 0.853 | MARS | Native |
| *Acer negundo* | 0.908 | 0.910 | 0.818 | MAXENT | Native |
| *Acer negundo* | 0.964 | 0.963 | 0.927 | GLM | All |
| *Acer negundo* | 0.952 | 0.937 | 0.889 | GAM | All |
| *Acer negundo* | 0.957 | 0.963 | 0.920 | GBM | All |
| *Acer negundo* | 0.965 | 0.959 | 0.924 | CTA | All |
| *Acer negundo* | 0.916 | 0.909 | 0.824 | ANN | All |
| *Acer negundo* | 0.970 | 0.969 | 0.939 | RF | All |
| *Acer negundo* | 0.971 | 0.937 | 0.909 | FDA | All |
| *Acer negundo* | 0.953 | 0.949 | 0.903 | MARS | All |
| *Acer negundo* | 0.930 | 0.905 | 0.835 | MAXENT | All |
| *Fraxinus pennsylvanica* | 0.911 | 0.878 | 0.790 | GLM | Non-Native |
| *Fraxinus pennsylvanica* | 0.901 | 0.894 | 0.795 | GAM | Non-Native |
| *Fraxinus pennsylvanica* | 0.935 | 0.915 | 0.849 | GBM | Non-Native |
| *Fraxinus pennsylvanica* | 0.914 | 0.889 | 0.803 | CTA | Non-Native |
| *Fraxinus pennsylvanica* | 0.857 | 0.894 | 0.751 | ANN | Non-Native |
| *Fraxinus pennsylvanica* | 0.939 | 0.927 | 0.866 | RF | Non-Native |
| *Fraxinus pennsylvanica* | 0.930 | 0.922 | 0.852 | FDA | Non-Native |
| *Fraxinus pennsylvanica* | 0.904 | 0.889 | 0.792 | MARS | Non-Native |
| *Fraxinus pennsylvanica* | 0.851 | 0.860 | 0.712 | MAXENT | Non-Native |
| *Fraxinus pennsylvanica* | 0.939 | 0.929 | 0.868 | GLM | Native |
| *Fraxinus pennsylvanica* | 0.939 | 0.929 | 0.868 | GAM | Native |
| *Fraxinus pennsylvanica* | 0.938 | 0.929 | 0.867 | GBM | Native |
| *Fraxinus pennsylvanica* | 0.930 | 0.928 | 0.858 | CTA | Native |
| *Fraxinus pennsylvanica* | 0.929 | 0.905 | 0.834 | ANN | Native |
| *Fraxinus pennsylvanica* | 0.939 | 0.929 | 0.868 | RF | Native |
| *Fraxinus pennsylvanica* | 0.939 | 0.929 | 0.868 | FDA | Native |
| *Fraxinus pennsylvanica* | 0.936 | 0.928 | 0.864 | MARS | Native |
| *Fraxinus pennsylvanica* | 0.931 | 0.919 | 0.849 | MAXENT | Native |
| *Fraxinus pennsylvanica* | 0.970 | 0.965 | 0.936 | GLM | All |
| *Fraxinus pennsylvanica* | 0.962 | 0.956 | 0.918 | GAM | All |
| *Fraxinus pennsylvanica* | 0.968 | 0.961 | 0.929 | GBM | All |
| *Fraxinus pennsylvanica* | 0.966 | 0.960 | 0.927 | CTA | All |
| *Fraxinus pennsylvanica* | 0.916 | 0.953 | 0.869 | ANN | All |
| *Fraxinus pennsylvanica* | 0.979 | 0.969 | 0.948 | RF | All |
| *Fraxinus pennsylvanica* | 0.968 | 0.963 | 0.931 | FDA | All |
| *Fraxinus pennsylvanica* | 0.972 | 0.961 | 0.933 | MARS | All |
| *Fraxinus pennsylvanica* | 0.934 | 0.950 | 0.884 | MAXENT | All |
| *Juglans nigra* | 0.922 | 0.914 | 0.836 | GLM | Non-Native |
| *Juglans nigra* | 0.920 | 0.889 | 0.809 | GAM | Non-Native |
| *Juglans nigra* | 0.932 | 0.914 | 0.846 | GBM | Non-Native |
| *Juglans nigra* | 0.921 | 0.914 | 0.835 | CTA | Non-Native |
| *Juglans nigra* | 0.939 | 0.846 | 0.785 | ANN | Non-Native |
| *Juglans nigra* | 0.939 | 0.929 | 0.868 | RF | Non-Native |
| *Juglans nigra* | 0.934 | 0.911 | 0.846 | FDA | Non-Native |
| *Juglans nigra* | 0.911 | 0.893 | 0.803 | MARS | Non-Native |
| *Juglans nigra* | 0.914 | 0.817 | 0.731 | MAXENT | Non-Native |
| *Juglans nigra* | 0.939 | 0.929 | 0.868 | GLM | Native |
| *Juglans nigra* | 0.938 | 0.928 | 0.867 | GAM | Native |
| *Juglans nigra* | 0.931 | 0.911 | 0.842 | GBM | Native |
| *Juglans nigra* | 0.938 | 0.929 | 0.867 | CTA | Native |
| *Juglans nigra* | 0.936 | 0.927 | 0.863 | ANN | Native |
| *Juglans nigra* | 0.939 | 0.911 | 0.850 | RF | Native |
| *Juglans nigra* | 0.939 | 0.928 | 0.867 | FDA | Native |
| *Juglans nigra* | 0.938 | 0.928 | 0.866 | MARS | Native |
| *Juglans nigra* | 0.933 | 0.923 | 0.856 | MAXENT | Native |
| *Juglans nigra* | 0.973 | 0.957 | 0.930 | GLM | All |
| *Juglans nigra* | 0.965 | 0.963 | 0.928 | GAM | All |
| *Juglans nigra* | 0.974 | 0.969 | 0.942 | GBM | All |
| *Juglans nigra* | 0.973 | 0.966 | 0.938 | CTA | All |
| *Juglans nigra* | 0.903 | 0.909 | 0.812 | ANN | All |
| *Juglans nigra* | 0.980 | 0.969 | 0.948 | RF | All |
| *Juglans nigra* | 0.980 | 0.927 | 0.907 | FDA | All |
| *Juglans nigra* | 0.968 | 0.956 | 0.924 | MARS | All |
| *Juglans nigra* | 0.967 | 0.938 | 0.905 | MAXENT | All |
| *Pinus contorta* | 0.939 | 0.923 | 0.862 | GLM | Non-Native |
| *Pinus contorta* | 0.926 | 0.914 | 0.840 | GAM | Non-Native |
| *Pinus contorta* | 0.928 | 0.917 | 0.845 | GBM | Non-Native |
| *Pinus contorta* | 0.923 | 0.917 | 0.839 | CTA | Non-Native |
| *Pinus contorta* | 0.903 | 0.871 | 0.774 | ANN | Non-Native |
| *Pinus contorta* | 0.939 | 0.929 | 0.868 | RF | Non-Native |
| *Pinus contorta* | 0.939 | 0.725 | 0.664 | FDA | Non-Native |
| *Pinus contorta* | 0.929 | 0.918 | 0.848 | MARS | Non-Native |
| *Pinus contorta* | 0.896 | 0.880 | 0.776 | MAXENT | Non-Native |
| *Pinus contorta* | 0.938 | 0.927 | 0.865 | GLM | Native |
| *Pinus contorta* | 0.935 | 0.926 | 0.861 | GAM | Native |
| *Pinus contorta* | 0.935 | 0.917 | 0.852 | GBM | Native |
| *Pinus contorta* | 0.931 | 0.918 | 0.849 | CTA | Native |
| *Pinus contorta* | 0.924 | 0.912 | 0.836 | ANN | Native |
| *Pinus contorta* | 0.931 | 0.921 | 0.851 | RF | Native |
| *Pinus contorta* | 0.936 | 0.923 | 0.860 | FDA | Native |
| *Pinus contorta* | 0.932 | 0.920 | 0.852 | MARS | Native |
| *Pinus contorta* | 0.932 | 0.920 | 0.852 | MAXENT | Native |
| *Pinus contorta* | 0.966 | 0.960 | 0.926 | GLM | All |
| *Pinus contorta* | 0.962 | 0.949 | 0.912 | GAM | All |
| *Pinus contorta* | 0.959 | 0.955 | 0.915 | GBM | All |
| *Pinus contorta* | 0.965 | 0.951 | 0.917 | CTA | All |
| *Pinus contorta* | 0.959 | 0.956 | 0.915 | ANN | All |
| *Pinus contorta* | 0.960 | 0.969 | 0.929 | RF | All |
| *Pinus contorta* | 0.965 | 0.960 | 0.925 | FDA | All |
| *Pinus contorta* | 0.970 | 0.960 | 0.930 | MARS | All |
| *Pinus contorta* | 0.866 | 0.904 | 0.770 | MAXENT | All |
| *Pseudotsuga menziesii* | 0.933 | 0.923 | 0.856 | GLM | Non-Native |
| *Pseudotsuga menziesii* | 0.927 | 0.920 | 0.846 | GAM | Non-Native |
| *Pseudotsuga menziesii* | 0.932 | 0.916 | 0.848 | GBM | Non-Native |
| *Pseudotsuga menziesii* | 0.929 | 0.918 | 0.848 | CTA | Non-Native |
| *Pseudotsuga menziesii* | 0.920 | 0.912 | 0.832 | ANN | Non-Native |
| *Pseudotsuga menziesii* | 0.940 | 0.929 | 0.869 | RF | Non-Native |
| *Pseudotsuga menziesii* | 0.931 | 0.915 | 0.846 | FDA | Non-Native |
| *Pseudotsuga menziesii* | 0.930 | 0.919 | 0.849 | MARS | Non-Native |
| *Pseudotsuga menziesii* | 0.915 | 0.914 | 0.829 | MAXENT | Non-Native |
| *Pseudotsuga menziesii* | 0.939 | 0.929 | 0.868 | GLM | Native |
| *Pseudotsuga menziesii* | 0.910 | 0.902 | 0.812 | GAM | Native |
| *Pseudotsuga menziesii* | 0.934 | 0.925 | 0.859 | GBM | Native |
| *Pseudotsuga menziesii* | 0.912 | 0.914 | 0.826 | CTA | Native |
| *Pseudotsuga menziesii* | 0.884 | 0.893 | 0.777 | ANN | Native |
| *Pseudotsuga menziesii* | 0.938 | 0.929 | 0.867 | RF | Native |
| *Pseudotsuga menziesii* | 0.936 | 0.918 | 0.854 | FDA | Native |
| *Pseudotsuga menziesii* | 0.925 | 0.909 | 0.834 | MARS | Native |
| *Pseudotsuga menziesii* | 0.906 | 0.877 | 0.782 | MAXENT | Native |
| *Pseudotsuga menziesii* | 0.971 | 0.958 | 0.929 | GLM | All |
| *Pseudotsuga menziesii* | 0.962 | 0.955 | 0.918 | GAM | All |
| *Pseudotsuga menziesii* | 0.969 | 0.959 | 0.928 | GBM | All |
| *Pseudotsuga menziesii* | 0.962 | 0.964 | 0.925 | CTA | All |
| *Pseudotsuga menziesii* | 0.957 | 0.940 | 0.897 | ANN | All |
| *Pseudotsuga menziesii* | 0.980 | 0.951 | 0.930 | RF | All |
| *Pseudotsuga menziesii* | 0.980 | 0.951 | 0.930 | FDA | All |
| *Pseudotsuga menziesii* | 0.964 | 0.958 | 0.922 | MARS | All |
| *Pseudotsuga menziesii* | 0.951 | 0.940 | 0.891 | MAXENT | All |
| *Picea pungens* | 0.939 | 0.929 | 0.868 | GLM | Non-Native |
| *Picea pungens* | 0.907 | 0.884 | 0.791 | GAM | Non-Native |
| *Picea pungens* | 0.935 | 0.917 | 0.852 | GBM | Non-Native |
| *Picea pungens* | 0.892 | 0.895 | 0.787 | CTA | Non-Native |
| *Picea pungens* | 0.916 | 0.844 | 0.761 | ANN | Non-Native |
| *Picea pungens* | 0.938 | 0.929 | 0.867 | RF | Non-Native |
| *Picea pungens* | 0.929 | 0.911 | 0.840 | FDA | Non-Native |
| *Picea pungens* | 0.918 | 0.906 | 0.824 | MARS | Non-Native |
| *Picea pungens* | 0.898 | 0.846 | 0.744 | MAXENT | Non-Native |
| *Picea pungens* | 0.939 | 0.929 | 0.868 | GLM | Native |
| *Picea pungens* | 0.939 | 0.929 | 0.868 | GAM | Native |
| *Picea pungens* | 0.926 | 0.929 | 0.855 | GBM | Native |
| *Picea pungens* | 0.899 | 0.921 | 0.819 | CTA | Native |
| *Picea pungens* | 0.896 | 0.911 | 0.807 | ANN | Native |
| *Picea pungens* | 0.939 | 0.929 | 0.868 | RF | Native |
| *Picea pungens* | 0.937 | 0.924 | 0.861 | FDA | Native |
| *Picea pungens* | 0.929 | 0.902 | 0.831 | MARS | Native |
| *Picea pungens* | 0.896 | 0.831 | 0.727 | MAXENT | Native |
| *Picea pungens* | 0.948 | 0.923 | 0.871 | GLM | All |
| *Picea pungens* | 0.909 | 0.914 | 0.823 | GAM | All |
| *Picea pungens* | 0.957 | 0.924 | 0.881 | GBM | All |
| *Picea pungens* | 0.955 | 0.928 | 0.882 | CTA | All |
| *Picea pungens* | 0.925 | 0.557 | 0.482 | ANN | All |
| *Picea pungens* | 0.979 | 0.969 | 0.948 | RF | All |
| *Picea pungens* | 0.931 | 0.943 | 0.874 | FDA | All |
| *Picea pungens* | 0.931 | 0.913 | 0.844 | MARS | All |
| *Picea pungens* | 0.865 | 0.772 | 0.638 | MAXENT | All |
| *Pinus radiata* | 0.939 | 0.929 | 0.868 | GLM | Non-Native |
| *Pinus radiata* | 0.939 | 0.929 | 0.868 | GAM | Non-Native |
| *Pinus radiata* | 0.939 | 0.927 | 0.866 | GBM | Non-Native |
| *Pinus radiata* | 0.933 | 0.911 | 0.844 | CTA | Non-Native |
| *Pinus radiata* | 0.925 | 0.914 | 0.839 | ANN | Non-Native |
| *Pinus radiata* | 0.939 | 0.929 | 0.868 | RF | Non-Native |
| *Pinus radiata* | 0.939 | 0.922 | 0.861 | FDA | Non-Native |
| *Pinus radiata* | 0.939 | 0.929 | 0.868 | MARS | Non-Native |
| *Pinus radiata* | 0.936 | 0.907 | 0.843 | MAXENT | Non-Native |
| *Pinus radiata* | 0.939 | 0.929 | 0.868 | GLM | Native |
| *Pinus radiata* | 0.939 | 0.929 | 0.868 | GAM | Native |
| *Pinus radiata* | 0.939 | 0.929 | 0.868 | GBM | Native |
| *Pinus radiata* | 0.939 | 0.929 | 0.868 | CTA | Native |
| *Pinus radiata* | 0.939 | 0.929 | 0.868 | ANN | Native |
| *Pinus radiata* | 0.939 | 0.929 | 0.868 | RF | Native |
| *Pinus radiata* | 0.939 | 0.929 | 0.868 | FDA | Native |
| *Pinus radiata* | 0.939 | 0.929 | 0.868 | MARS | Native |
| *Pinus radiata* | 0.939 | 0.929 | 0.868 | MAXENT | Native |
| *Pinus radiata* | 0.972 | 0.938 | 0.910 | GLM | All |
| *Pinus radiata* | 0.973 | 0.967 | 0.940 | GAM | All |
| *Pinus radiata* | 0.979 | 0.966 | 0.944 | GBM | All |
| *Pinus radiata* | 0.977 | 0.951 | 0.928 | CTA | All |
| *Pinus radiata* | 0.977 | 0.962 | 0.939 | ANN | All |
| *Pinus radiata* | 0.979 | 0.969 | 0.948 | RF | All |
| *Pinus radiata* | 0.979 | 0.957 | 0.936 | FDA | All |
| *Pinus radiata* | 0.979 | 0.969 | 0.948 | MARS | All |
| *Pinus radiata* | 0.970 | 0.956 | 0.927 | MAXENT | All |
| *Prunus serotina* | 0.936 | 0.929 | 0.865 | GLM | Non-Native |
| *Prunus serotina* | 0.926 | 0.923 | 0.849 | GAM | Non-Native |
| *Prunus serotina* | 0.929 | 0.926 | 0.856 | GBM | Non-Native |
| *Prunus serotina* | 0.928 | 0.925 | 0.853 | CTA | Non-Native |
| *Prunus serotina* | 0.913 | 0.885 | 0.797 | ANN | Non-Native |
| *Prunus serotina* | 0.940 | 0.929 | 0.869 | RF | Non-Native |
| *Prunus serotina* | 0.936 | 0.917 | 0.853 | FDA | Non-Native |
| *Prunus serotina* | 0.934 | 0.912 | 0.846 | MARS | Non-Native |
| *Prunus serotina* | 0.910 | 0.903 | 0.814 | MAXENT | Non-Native |
| *Prunus serotina* | 0.939 | 0.929 | 0.868 | GLM | Native |
| *Prunus serotina* | 0.939 | 0.929 | 0.868 | GAM | Native |
| *Prunus serotina* | 0.934 | 0.929 | 0.863 | GBM | Native |
| *Prunus serotina* | 0.927 | 0.925 | 0.853 | CTA | Native |
| *Prunus serotina* | 0.927 | 0.911 | 0.839 | ANN | Native |
| *Prunus serotina* | 0.939 | 0.929 | 0.868 | RF | Native |
| *Prunus serotina* | 0.939 | 0.929 | 0.868 | FDA | Native |
| *Prunus serotina* | 0.905 | 0.837 | 0.742 | MARS | Native |
| *Prunus serotina* | 0.930 | 0.893 | 0.823 | MAXENT | Native |
| *Prunus serotina* | 0.970 | 0.965 | 0.935 | GLM | All |
| *Prunus serotina* | 0.967 | 0.958 | 0.925 | GAM | All |
| *Prunus serotina* | 0.966 | 0.962 | 0.929 | GBM | All |
| *Prunus serotina* | 0.967 | 0.959 | 0.926 | CTA | All |
| *Prunus serotina* | 0.967 | 0.937 | 0.903 | ANN | All |
| *Prunus serotina* | 0.960 | 0.969 | 0.929 | RF | All |
| *Prunus serotina* | 0.960 | 0.969 | 0.929 | FDA | All |
| *Prunus serotina* | 0.970 | 0.950 | 0.920 | MARS | All |
| *Prunus serotina* | 0.962 | 0.939 | 0.902 | MAXENT | All |
| *Picea sitchensis* | 0.934 | 0.925 | 0.859 | GLM | Non-Native |
| *Picea sitchensis* | 0.935 | 0.925 | 0.860 | GAM | Non-Native |
| *Picea sitchensis* | 0.935 | 0.925 | 0.861 | GBM | Non-Native |
| *Picea sitchensis* | 0.930 | 0.921 | 0.850 | CTA | Non-Native |
| *Picea sitchensis* | 0.932 | 0.917 | 0.849 | ANN | Non-Native |
| *Picea sitchensis* | 0.940 | 0.929 | 0.869 | RF | Non-Native |
| *Picea sitchensis* | 0.936 | 0.924 | 0.860 | FDA | Non-Native |
| *Picea sitchensis* | 0.932 | 0.926 | 0.859 | MARS | Non-Native |
| *Picea sitchensis* | 0.928 | 0.921 | 0.849 | MAXENT | Non-Native |
| *Picea sitchensis* | 0.931 | 0.902 | 0.833 | GLM | Native |
| *Picea sitchensis* | 0.931 | 0.884 | 0.815 | GAM | Native |
| *Picea sitchensis* | 0.936 | 0.929 | 0.865 | GBM | Native |
| *Picea sitchensis* | 0.934 | 0.927 | 0.861 | CTA | Native |
| *Picea sitchensis* | 0.930 | 0.909 | 0.839 | ANN | Native |
| *Picea sitchensis* | 0.939 | 0.929 | 0.868 | RF | Native |
| *Picea sitchensis* | 0.939 | 0.763 | 0.702 | FDA | Native |
| *Picea sitchensis* | 0.937 | 0.925 | 0.862 | MARS | Native |
| *Picea sitchensis* | 0.934 | 0.920 | 0.854 | MAXENT | Native |
| *Picea sitchensis* | 0.978 | 0.968 | 0.946 | GLM | All |
| *Picea sitchensis* | 0.976 | 0.964 | 0.940 | GAM | All |
| *Picea sitchensis* | 0.976 | 0.964 | 0.941 | GBM | All |
| *Picea sitchensis* | 0.974 | 0.965 | 0.938 | CTA | All |
| *Picea sitchensis* | 0.961 | 0.953 | 0.913 | ANN | All |
| *Picea sitchensis* | 0.980 | 0.969 | 0.948 | RF | All |
| *Picea sitchensis* | 0.975 | 0.963 | 0.938 | FDA | All |
| *Picea sitchensis* | 0.975 | 0.964 | 0.939 | MARS | All |
| *Picea sitchensis* | 0.968 | 0.959 | 0.927 | MAXENT | All |
| *Pinus strobus* | 0.931 | 0.924 | 0.855 | GLM | Non-Native |
| *Pinus strobus* | 0.917 | 0.918 | 0.835 | GAM | Non-Native |
| *Pinus strobus* | 0.934 | 0.918 | 0.852 | GBM | Non-Native |
| *Pinus strobus* | 0.925 | 0.916 | 0.842 | CTA | Non-Native |
| *Pinus strobus* | 0.912 | 0.887 | 0.799 | ANN | Non-Native |
| *Pinus strobus* | 0.940 | 0.929 | 0.869 | RF | Non-Native |
| *Pinus strobus* | 0.928 | 0.916 | 0.844 | FDA | Non-Native |
| *Pinus strobus* | 0.925 | 0.921 | 0.846 | MARS | Non-Native |
| *Pinus strobus* | 0.902 | 0.899 | 0.801 | MAXENT | Non-Native |
| *Pinus strobus* | 0.939 | 0.929 | 0.868 | GLM | Native |
| *Pinus strobus* | 0.939 | 0.929 | 0.868 | GAM | Native |
| *Pinus strobus* | 0.938 | 0.929 | 0.867 | GBM | Native |
| *Pinus strobus* | 0.922 | 0.921 | 0.843 | CTA | Native |
| *Pinus strobus* | 0.933 | 0.902 | 0.835 | ANN | Native |
| *Pinus strobus* | 0.939 | 0.929 | 0.868 | RF | Native |
| *Pinus strobus* | 0.939 | 0.929 | 0.868 | FDA | Native |
| *Pinus strobus* | 0.939 | 0.929 | 0.868 | MARS | Native |
| *Pinus strobus* | 0.906 | 0.911 | 0.817 | MAXENT | Native |
| *Pinus strobus* | 0.973 | 0.962 | 0.935 | GLM | All |
| *Pinus strobus* | 0.957 | 0.940 | 0.897 | GAM | All |
| *Pinus strobus* | 0.972 | 0.953 | 0.924 | GBM | All |
| *Pinus strobus* | 0.963 | 0.958 | 0.920 | CTA | All |
| *Pinus strobus* | 0.928 | 0.927 | 0.855 | ANN | All |
| *Pinus strobus* | 0.980 | 0.969 | 0.949 | RF | All |
| *Pinus strobus* | 0.980 | 0.969 | 0.949 | FDA | All |
| *Pinus strobus* | 0.962 | 0.952 | 0.914 | MARS | All |
| *Pinus strobus* | 0.944 | 0.924 | 0.868 | MAXENT | All |
| *Quercus rubra* | 0.932 | 0.917 | 0.849 | GLM | Non-Native |
| *Quercus rubra* | 0.927 | 0.922 | 0.849 | GAM | Non-Native |
| *Quercus rubra* | 0.929 | 0.922 | 0.851 | GBM | Non-Native |
| *Quercus rubra* | 0.926 | 0.920 | 0.846 | CTA | Non-Native |
| *Quercus rubra* | 0.912 | 0.917 | 0.829 | ANN | Non-Native |
| *Quercus rubra* | 0.921 | 0.929 | 0.850 | RF | Non-Native |
| *Quercus rubra* | 0.931 | 0.922 | 0.853 | FDA | Non-Native |
| *Quercus rubra* | 0.929 | 0.921 | 0.850 | MARS | Non-Native |
| *Quercus rubra* | 0.911 | 0.916 | 0.827 | MAXENT | Non-Native |
| *Quercus rubra* | 0.930 | 0.909 | 0.839 | GLM | Native |
| *Quercus rubra* | 0.939 | 0.929 | 0.868 | GAM | Native |
| *Quercus rubra* | 0.939 | 0.925 | 0.864 | GBM | Native |
| *Quercus rubra* | 0.925 | 0.918 | 0.843 | CTA | Native |
| *Quercus rubra* | 0.922 | 0.888 | 0.810 | ANN | Native |
| *Quercus rubra* | 0.939 | 0.929 | 0.868 | RF | Native |
| *Quercus rubra* | 0.937 | 0.928 | 0.865 | FDA | Native |
| *Quercus rubra* | 0.935 | 0.916 | 0.851 | MARS | Native |
| *Quercus rubra* | 0.927 | 0.902 | 0.829 | MAXENT | Native |
| *Quercus rubra* | 0.973 | 0.965 | 0.938 | GLM | All |
| *Quercus rubra* | 0.962 | 0.964 | 0.926 | GAM | All |
| *Quercus rubra* | 0.967 | 0.960 | 0.927 | GBM | All |
| *Quercus rubra* | 0.964 | 0.964 | 0.928 | CTA | All |
| *Quercus rubra* | 0.957 | 0.947 | 0.904 | ANN | All |
| *Quercus rubra* | 0.979 | 0.969 | 0.948 | RF | All |
| *Quercus rubra* | 0.972 | 0.953 | 0.925 | FDA | All |
| *Quercus rubra* | 0.968 | 0.957 | 0.924 | MARS | All |
| *Quercus rubra* | 0.952 | 0.944 | 0.896 | MAXENT | All |
| *Robinia pseudoacacia* | 0.933 | 0.921 | 0.854 | GLM | Non-Native |
| *Robinia pseudoacacia* | 0.926 | 0.911 | 0.837 | GAM | Non-Native |
| *Robinia pseudoacacia* | 0.929 | 0.915 | 0.844 | GBM | Non-Native |
| *Robinia pseudoacacia* | 0.930 | 0.911 | 0.841 | CTA | Non-Native |
| *Robinia pseudoacacia* | 0.919 | 0.877 | 0.796 | ANN | Non-Native |
| *Robinia pseudoacacia* | 0.939 | 0.921 | 0.860 | RF | Non-Native |
| *Robinia pseudoacacia* | 0.933 | 0.918 | 0.851 | FDA | Non-Native |
| *Robinia pseudoacacia* | 0.930 | 0.920 | 0.850 | MARS | Non-Native |
| *Robinia pseudoacacia* | 0.906 | 0.890 | 0.795 | MAXENT | Non-Native |
| *Robinia pseudoacacia* | 0.918 | 0.911 | 0.830 | GLM | Native |
| *Robinia pseudoacacia* | 0.939 | 0.929 | 0.868 | GAM | Native |
| *Robinia pseudoacacia* | 0.934 | 0.924 | 0.858 | GBM | Native |
| *Robinia pseudoacacia* | 0.915 | 0.918 | 0.833 | CTA | Native |
| *Robinia pseudoacacia* | 0.922 | 0.877 | 0.799 | ANN | Native |
| *Robinia pseudoacacia* | 0.939 | 0.929 | 0.868 | RF | Native |
| *Robinia pseudoacacia* | 0.934 | 0.927 | 0.861 | FDA | Native |
| *Robinia pseudoacacia* | 0.922 | 0.921 | 0.842 | MARS | Native |
| *Robinia pseudoacacia* | 0.912 | 0.877 | 0.790 | MAXENT | Native |
| *Robinia pseudoacacia* | 0.963 | 0.959 | 0.922 | GLM | All |
| *Robinia pseudoacacia* | 0.953 | 0.944 | 0.897 | GAM | All |
| *Robinia pseudoacacia* | 0.964 | 0.949 | 0.913 | GBM | All |
| *Robinia pseudoacacia* | 0.958 | 0.947 | 0.906 | CTA | All |
| *Robinia pseudoacacia* | 0.959 | 0.878 | 0.836 | ANN | All |
| *Robinia pseudoacacia* | 0.980 | 0.969 | 0.949 | RF | All |
| *Robinia pseudoacacia* | 0.980 | 0.969 | 0.949 | FDA | All |
| *Robinia pseudoacacia* | 0.954 | 0.944 | 0.898 | MARS | All |
| *Robinia pseudoacacia* | 0.937 | 0.916 | 0.853 | MAXENT | All |
| *Thuja plicata* | 0.922 | 0.921 | 0.842 | GLM | Non-Native |
| *Thuja plicata* | 0.920 | 0.925 | 0.845 | GAM | Non-Native |
| *Thuja plicata* | 0.933 | 0.928 | 0.861 | GBM | Non-Native |
| *Thuja plicata* | 0.922 | 0.928 | 0.849 | CTA | Non-Native |
| *Thuja plicata* | 0.922 | 0.928 | 0.849 | ANN | Non-Native |
| *Thuja plicata* | 0.938 | 0.929 | 0.867 | RF | Non-Native |
| *Thuja plicata* | 0.933 | 0.929 | 0.862 | FDA | Non-Native |
| *Thuja plicata* | 0.926 | 0.925 | 0.852 | MARS | Non-Native |
| *Thuja plicata* | 0.904 | 0.929 | 0.833 | MAXENT | Non-Native |
| *Thuja plicata* | 0.939 | 0.929 | 0.868 | GLM | Native |
| *Thuja plicata* | 0.939 | 0.929 | 0.868 | GAM | Native |
| *Thuja plicata* | 0.939 | 0.929 | 0.868 | GBM | Native |
| *Thuja plicata* | 0.860 | 0.921 | 0.781 | CTA | Native |
| *Thuja plicata* | 0.939 | 0.911 | 0.850 | ANN | Native |
| *Thuja plicata* | 0.939 | 0.929 | 0.868 | RF | Native |
| *Thuja plicata* | 0.939 | 0.929 | 0.868 | FDA | Native |
| *Thuja plicata* | 0.939 | 0.929 | 0.868 | MARS | Native |
| *Thuja plicata* | 0.939 | 0.911 | 0.850 | MAXENT | Native |
| *Thuja plicata* | 0.973 | 0.956 | 0.929 | GLM | All |
| *Thuja plicata* | 0.968 | 0.954 | 0.922 | GAM | All |
| *Thuja plicata* | 0.968 | 0.968 | 0.936 | GBM | All |
| *Thuja plicata* | 0.971 | 0.951 | 0.922 | CTA | All |
| *Thuja plicata* | 0.951 | 0.939 | 0.889 | ANN | All |
| *Thuja plicata* | 0.979 | 0.969 | 0.948 | RF | All |
| *Thuja plicata* | 0.973 | 0.960 | 0.933 | FDA | All |
| *Thuja plicata* | 0.975 | 0.968 | 0.943 | MARS | All |
| *Thuja plicata* | 0.952 | 0.939 | 0.891 | MAXENT | All |

### **Table S8:** Statistics for evaluation of the ensemble SDM for the target NNTs calibrated with native, non-native, and all (native + introduced range of the restive NNTs) occurrence data.

| **Species** | **Calibration range** | **Sensitivity** | **Specificity** | **TSS** |
| --- | --- | --- | --- | --- |
| *A dealbata* | All | 0.974 | 0.96 | 0.932 |
| *A dealbata* | Native | 0.939 | 0.929 | 0.868 |
| *A dealbata* | Non-Native | 0.933 | 0.92 | 0.85 |
| *A grandis* | All | 0.969 | 0.956 | 0.925 |
| *A grandis* | Native | 0.938 | 0.927 | 0.863 |
| *A grandis* | Non-Native | 0.927 | 0.917 | 0.843 |
| *A negundo* | All | 0.957 | 0.949 | 0.909 |
| *A negundo* | Native | 0.933 | 0.926 | 0.858 |
| *A negundo* | Non-Native | 0.92 | 0.913 | 0.835 |
| *F pennsylvanica* | All | 0.968 | 0.961 | 0.929 |
| *F pennsylvanica* | Native | 0.938 | 0.929 | 0.867 |
| *F pennsylvanica* | Non-Native | 0.911 | 0.894 | 0.795 |
| *J nigra* | All | 0.973 | 0.957 | 0.928 |
| *J nigra* | Native | 0.938 | 0.928 | 0.866 |
| *J nigra* | Non-Native | 0.922 | 0.911 | 0.835 |
| *P contorta* | All | 0.962 | 0.956 | 0.917 |
| *P contorta* | Native | 0.932 | 0.92 | 0.852 |
| *P contorta* | Non-Native | 0.928 | 0.917 | 0.84 |
| *P menziesii* | All | 0.964 | 0.955 | 0.925 |
| *P menziesii* | Native | 0.925 | 0.914 | 0.834 |
| *P menziesii* | Non-Native | 0.93 | 0.918 | 0.848 |
| *P pungens* | All | 0.931 | 0.923 | 0.871 |
| *P pungens* | Native | 0.929 | 0.924 | 0.855 |
| *P pungens* | Non-Native | 0.918 | 0.906 | 0.824 |
| *P radiata* | All | 0.977 | 0.962 | 0.939 |
| *P radiata* | Native | 0.939 | 0.929 | 0.868 |
| *P radiata* | Non-Native | 0.939 | 0.927 | 0.866 |
| *P serotina* | All | 0.967 | 0.959 | 0.926 |
| *P serotina* | Native | 0.934 | 0.929 | 0.863 |
| *P serotina* | Non-Native | 0.929 | 0.923 | 0.853 |
| *P sitchensis* | All | 0.975 | 0.964 | 0.939 |
| *P sitchensis* | Native | 0.934 | 0.92 | 0.854 |
| *P sitchensis* | Non-Native | 0.934 | 0.925 | 0.859 |
| *P strobus* | All | 0.963 | 0.953 | 0.92 |
| *P strobus* | Native | 0.939 | 0.929 | 0.868 |
| *P strobus* | Non-Native | 0.925 | 0.918 | 0.844 |
| *Q rubra* | All | 0.967 | 0.96 | 0.926 |
| *Q rubra* | Native | 0.935 | 0.918 | 0.851 |
| *Q rubra* | Non-Native | 0.927 | 0.921 | 0.849 |
| *R pseudoacacia* | All | 0.959 | 0.947 | 0.906 |
| *R pseudoacacia* | Native | 0.922 | 0.921 | 0.842 |
| *R pseudoacacia* | Non-Native | 0.93 | 0.915 | 0.844 |
| *T plicata* | All | 0.971 | 0.956 | 0.929 |
| *T plicata* | Native | 0.939 | 0.929 | 0.868 |
| *T plicata* | Non-Native | 0.922 | 0.928 | 0.849 |
|  |  |  |  |  |

### **Table S9:** Statistics for evaluation for each of the models used to develop the ensemble SDM for the native species

| ***Species*** | **TSS** | **Sensitivity** | **Specificity** | **Model** |
| --- | --- | --- | --- | --- |
| *Abies alba* | 0.92 | 0.97 | 0.95 | GLM |
| *Abies alba* | 0.95 | 0.97 | 0.98 | GBM |
| *Abies alba* | 0.96 | 0.98 | 0.98 | GAM |
| *Abies alba* | 0.95 | 0.96 | 0.99 | CTA |
| *Abies alba* | 0.97 | 0.98 | 0.99 | ANN |
| *Abies alba* | 0.76 | 0.8 | 0.96 | SRE |
| *Abies alba* | 0.94 | 0.98 | 0.96 | FDA |
| *Abies alba* | 0.94 | 0.97 | 0.97 | MARS |
| *Abies alba* | 0.99 | 0.99 | 1 | RF |
| *Abies alba* | 0.9 | 0.95 | 0.95 | MAXENT |
| *Fagus sylvatica* | 0.82 | 0.94 | 0.88 | GLM |
| *Fagus sylvatica* | 0.84 | 0.94 | 0.9 | GBM |
| *Fagus sylvatica* | 0.84 | 0.95 | 0.89 | GAM |
| *Fagus sylvatica* | 0.86 | 0.94 | 0.92 | CTA |
| *Fagus sylvatica* | 0.86 | 0.95 | 0.91 | ANN |
| *Fagus sylvatica* | 0.6 | 0.76 | 0.84 | SRE |
| *Fagus sylvatica* | 0.81 | 0.92 | 0.89 | FDA |
| *Fagus sylvatica* | 0.83 | 0.94 | 0.89 | MARS |
| *Fagus sylvatica* | 0.95 | 0.98 | 0.97 | RF |
| *Fagus sylvatica* | 0.77 | 0.95 | 0.82 | MAXENT |
| *Larix decidua* | 0.88 | 0.96 | 0.92 | GLM |
| *Larix decidua* | 0.91 | 0.96 | 0.95 | GBM |
| *Larix decidua* | 0.93 | 0.97 | 0.96 | GAM |
| *Larix decidua* | 0.92 | 0.97 | 0.95 | CTA |
| *Larix decidua* | 0.95 | 0.98 | 0.97 | ANN |
| *Larix decidua* | 0.67 | 0.78 | 0.89 | SRE |
| *Larix decidua* | 0.89 | 0.97 | 0.92 | FDA |
| *Larix decidua* | 0.9 | 0.97 | 0.93 | MARS |
| *Larix decidua* | 0.98 | 0.99 | 0.99 | RF |
| *Larix decidua* | 0.77 | 0.96 | 0.81 | MAXENT |
| *Picea abies* | 0.86 | 0.93 | 0.93 | GLM |
| *Picea abies* | 0.92 | 0.98 | 0.94 | GBM |
| *Picea abies* | 0.92 | 0.96 | 0.96 | GAM |
| *Picea abies* | 0.94 | 0.99 | 0.95 | CTA |
| *Picea abies* | 0.89 | 0.97 | 0.92 | ANN |
| *Picea abies* | 0.66 | 0.76 | 0.9 | SRE |
| *Picea abies* | 0.85 | 0.92 | 0.93 | FDA |
| *Picea abies* | 0.88 | 0.94 | 0.94 | MARS |
| *Picea abies* | 1 | 1 | 1 | RF |
| *Picea abies* | 0.62 | 0.95 | 0.67 | MAXENT |
| *Pinus sylvestris* | 0.81 | 0.92 | 0.89 | GLM |
| *Pinus sylvestris* | 0.88 | 0.98 | 0.9 | GBM |
| *Pinus sylvestris* | 0.87 | 0.95 | 0.92 | GAM |
| *Pinus sylvestris* | 0.92 | 0.99 | 0.93 | CTA |
| *Pinus sylvestris* | 0.9 | 0.97 | 0.93 | ANN |
| *Pinus sylvestris* | 0.6 | 0.77 | 0.83 | SRE |
| *Pinus sylvestris* | 0.82 | 0.93 | 0.89 | FDA |
| *Pinus sylvestris* | 0.85 | 0.96 | 0.89 | MARS |
| *Pinus sylvestris* | 0.99 | 1 | 0.99 | RF |
| *Pinus sylvestris* | 0.52 | 0.94 | 0.58 | MAXENT |
| *Quercus robur* | 0.87 | 0.93 | 0.94 | GLM |
| *Quercus robur* | 0.91 | 0.97 | 0.94 | GBM |
| *Quercus robur* | 0.9 | 0.96 | 0.94 | GAM |
| *Quercus robur* | 0.93 | 0.97 | 0.96 | CTA |
| *Quercus robur* | 0.94 | 0.98 | 0.96 | ANN |
| *Quercus robur* | 0.74 | 0.78 | 0.96 | SRE |
| *Quercus robur* | 0.86 | 0.93 | 0.93 | FDA |
| *Quercus robur* | 0.88 | 0.94 | 0.94 | MARS |
| *Quercus robur* | 0.99 | 1 | 0.99 | RF |
| *Quercus robur* | 0.81 | 0.97 | 0.84 | MAXENT |
| *Quercus petraea* | 0.77 | 0.96 | 0.81 | GLM |
| *Quercus petraea* | 0.81 | 0.91 | 0.9 | GBM |
| *Quercus petraea* | 0.82 | 0.93 | 0.89 | GAM |
| *Quercus petraea* | 0.85 | 0.95 | 0.9 | CTA |
| *Quercus petraea* | 0.85 | 0.94 | 0.91 | ANN |
| *Quercus petraea* | 0.67 | 0.8 | 0.87 | SRE |
| *Quercus petraea* | 0.78 | 0.93 | 0.85 | FDA |
| *Quercus petraea* | 0.8 | 0.96 | 0.84 | MARS |
| *Quercus petraea* | 0.92 | 0.96 | 0.96 | RF |
| *Quercus petraea* | 0.7 | 0.93 | 0.77 | MAXENT |
| *Pinus halepensis* | 0.78 | 0.91 | 0.87 | GLM |
| *Pinus halepensis* | 0.85 | 0.97 | 0.88 | GBM |
| *Pinus halepensis* | 0.84 | 0.94 | 0.9 | GAM |
| *Pinus halepensis* | 0.89 | 0.98 | 0.91 | CTA |
| *Pinus halepensis* | 0.88 | 0.96 | 0.92 | ANN |
| *Pinus halepensis* | 0.57 | 0.76 | 0.81 | SRE |
| *Pinus halepensis* | 0.79 | 0.91 | 0.88 | FDA |
| *Pinus halepensis* | 0.82 | 0.95 | 0.87 | MARS |
| *Pinus halepensis* | 0.97 | 0.99 | 0.98 | RF |
| *Pinus halepensis* | 0.49 | 0.93 | 0.56 | MAXENT |
| *Pinus nigra* | 0.73 | 0.94 | 0.79 | GLM |
| *Pinus nigra* | 0.78 | 0.9 | 0.88 | GBM |
| *Pinus nigra* | 0.8 | 0.92 | 0.88 | GAM |
| *Pinus nigra* | 0.82 | 0.94 | 0.88 | CTA |
| *Pinus nigra* | 0.83 | 0.93 | 0.9 | ANN |
| *Pinus nigra* | 0.64 | 0.79 | 0.85 | SRE |
| *Pinus nigra* | 0.75 | 0.92 | 0.83 | FDA |
| *Pinus nigra* | 0.77 | 0.95 | 0.82 | MARS |
| *Pinus nigra* | 0.89 | 0.95 | 0.94 | RF |
| *Pinus nigra* | 0.67 | 0.92 | 0.75 | MAXENT |
| *Pinus pinea* | 0.84 | 0.92 | 0.92 | GLM |
| *Pinus pinea* | 0.88 | 0.96 | 0.92 | GBM |
| *Pinus pinea* | 0.87 | 0.95 | 0.92 | GAM |
| *Pinus pinea* | 0.9 | 0.96 | 0.94 | CTA |
| *Pinus pinea* | 0.91 | 0.97 | 0.94 | ANN |
| *Pinus pinea* | 0.71 | 0.77 | 0.94 | SRE |
| *Pinus pinea* | 0.83 | 0.92 | 0.91 | FDA |
| *Pinus pinea* | 0.84 | 0.92 | 0.92 | MARS |
| *Pinus pinea* | 0.95 | 0.98 | 0.97 | RF |
| *Pinus pinea* | 0.78 | 0.96 | 0.82 | MAXENT |
| *Quercus ilex* | 0.82 | 0.91 | 0.91 | GLM |
| *Quercus ilex* | 0.88 | 0.96 | 0.92 | GBM |
| *Quercus ilex* | 0.89 | 0.95 | 0.94 | GAM |
| *Quercus ilex* | 0.9 | 0.97 | 0.93 | CTA |
| *Quercus ilex* | 0.86 | 0.96 | 0.9 | ANN |
| *Quercus ilex* | 0.63 | 0.75 | 0.88 | SRE |
| *Quercus ilex* | 0.82 | 0.91 | 0.91 | FDA |
| *Quercus ilex* | 0.85 | 0.93 | 0.92 | MARS |
| *Quercus ilex* | 0.97 | 0.99 | 0.98 | RF |
| *Quercus ilex* | 0.59 | 0.94 | 0.65 | MAXENT |
| *Quercus pubescens* | 0.85 | 0.95 | 0.9 | GLM |
| *Quercus pubescens* | 0.88 | 0.95 | 0.93 | GBM |
| *Quercus pubescens* | 0.9 | 0.96 | 0.94 | GAM |
| *Quercus pubescens* | 0.89 | 0.96 | 0.93 | CTA |
| *Quercus pubescens* | 0.91 | 0.96 | 0.95 | ANN |
| *Quercus pubescens* | 0.64 | 0.77 | 0.87 | SRE |
| *Quercus pubescens* | 0.86 | 0.95 | 0.91 | FDA |
| *Quercus pubescens* | 0.87 | 0.96 | 0.91 | MARS |
| *Quercus pubescens* | 0.95 | 0.98 | 0.97 | RF |
| *Quercus pubescens* | 0.74 | 0.95 | 0.79 | MAXENT |
| *Quercus suber* | 0.82 | 0.91 | 0.91 | GLM |
| *Quercus suber* | 0.88 | 0.96 | 0.92 | GBM |
| *Quercus suber* | 0.89 | 0.95 | 0.94 | GAM |
| *Quercus suber* | 0.9 | 0.97 | 0.93 | CTA |
| *Quercus suber* | 0.86 | 0.96 | 0.9 | ANN |
| *Quercus suber* | 0.63 | 0.75 | 0.88 | SRE |
| *Quercus suber* | 0.82 | 0.91 | 0.91 | FDA |
| *Quercus suber* | 0.85 | 0.93 | 0.92 | MARS |
| *Quercus suber* | 0.97 | 0.99 | 0.98 | RF |
| *Quercus suber* | 0.59 | 0.94 | 0.65 | MAXENT |

### **Table S10**: Statistics for evaluation of the ensemble SDM for the 13 native tree species.

| **Species** | **Sensitivity** | **Specificity** | **TSS** |
| --- | --- | --- | --- |
| *Abies alba* | 0.96 | 0.97 | 0.93 |
| *Fagus sylvatica* | 0.93 | 0.89 | 0.82 |
| *Larix decidua* | 0.95 | 0.93 | 0.88 |
| *Picea abies* | 0.94 | 0.91 | 0.85 |
| *Pinus halepensis* | 0.93 | 0.86 | 0.79 |
| *Pinus nigra* | 0.92 | 0.85 | 0.77 |
| *Pinus pinea* | 0.93 | 0.92 | 0.85 |
| *Pinus sylvestris* | 0.94 | 0.88 | 0.82 |
| *Quercus ilex* | 0.93 | 0.89 | 0.82 |
| *Quercus petraea* | 0.93 | 0.87 | 0.80 |
| *Quercus pubescens* | 0.94 | 0.91 | 0.85 |
| *Quercus robur* | 0.94 | 0.94 | 0.88 |
| *Quercus suber* | 0.93 | 0.89 | 0.82 |

### **Table S11.** Cut-off values used by biomod2 to convert probabilities (0-1) to binary values of presence= 1 and absence= 0 by maximizing TSS. These cut-off values of the ensemble models were only used to convert the predicted probability of occurrence of NNTs to estimate their range shifts in each 1x1 km forested grid cell of Europe.

| **Species type** | **Species** | **Cutoff** |
| --- | --- | --- |
| NNT | *Abies Grandis* | 0.62 |
| NNT | *Acacia dealbata* | 0.45 |
| NNT | *Acer negundo* | 0.73 |
| NNT | *Fraxinus pennsylvanica* | 0.52 |
| NNT | *Juglans nigra* | 0.60 |
| NNT | *Picea pungens* | 0.52 |
| NNT | *Picea sitchensis* | 0.48 |
| NNT | *Pinus contorta* | 0.69 |
| NNT | *Pinus radiata* | 0.49 |
| NNT | *Pinus strobus* | 0.51 |
| NNT | *Prunus serotina* | 0.70 |
| NNT | *Pseudotsuga menziesii* | 0.56 |
| NNT | *Quercus rubra* | 0.54 |
| NNT | *Robinia pseudoacacia* | 0.66 |
| NNT | *Thuja plicata* | 0.50 |

### **Table S12**: Summary of the climatic suitability of native and NNT species within each bioclimatic region of Europe. (Given as a separate csv file).


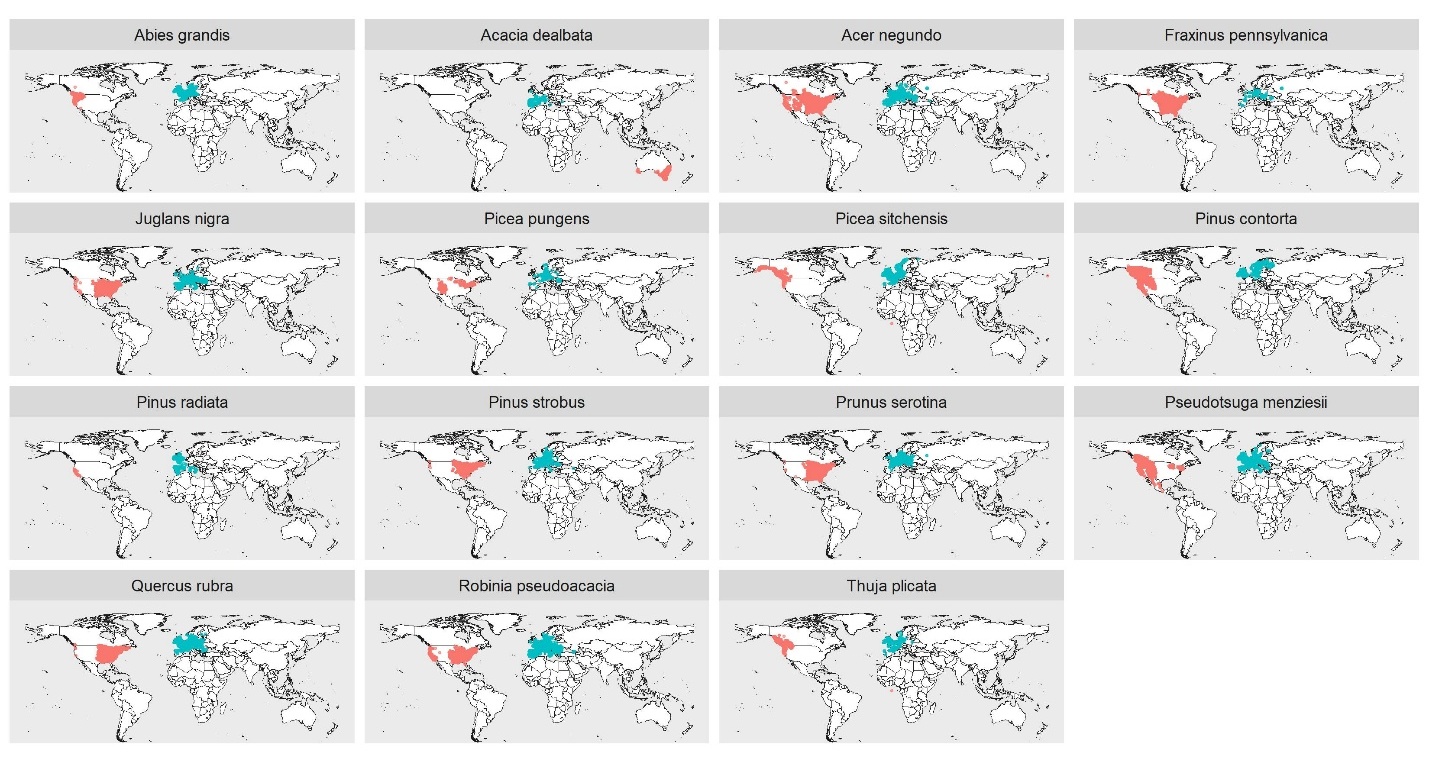


### **Fig. S1** Distribution of the presence locations of the 15 NNT species. Red points indicate the observed native distribution of the respective species, while blue points indicate the observed presence locations in their introduced range in Europe.

***
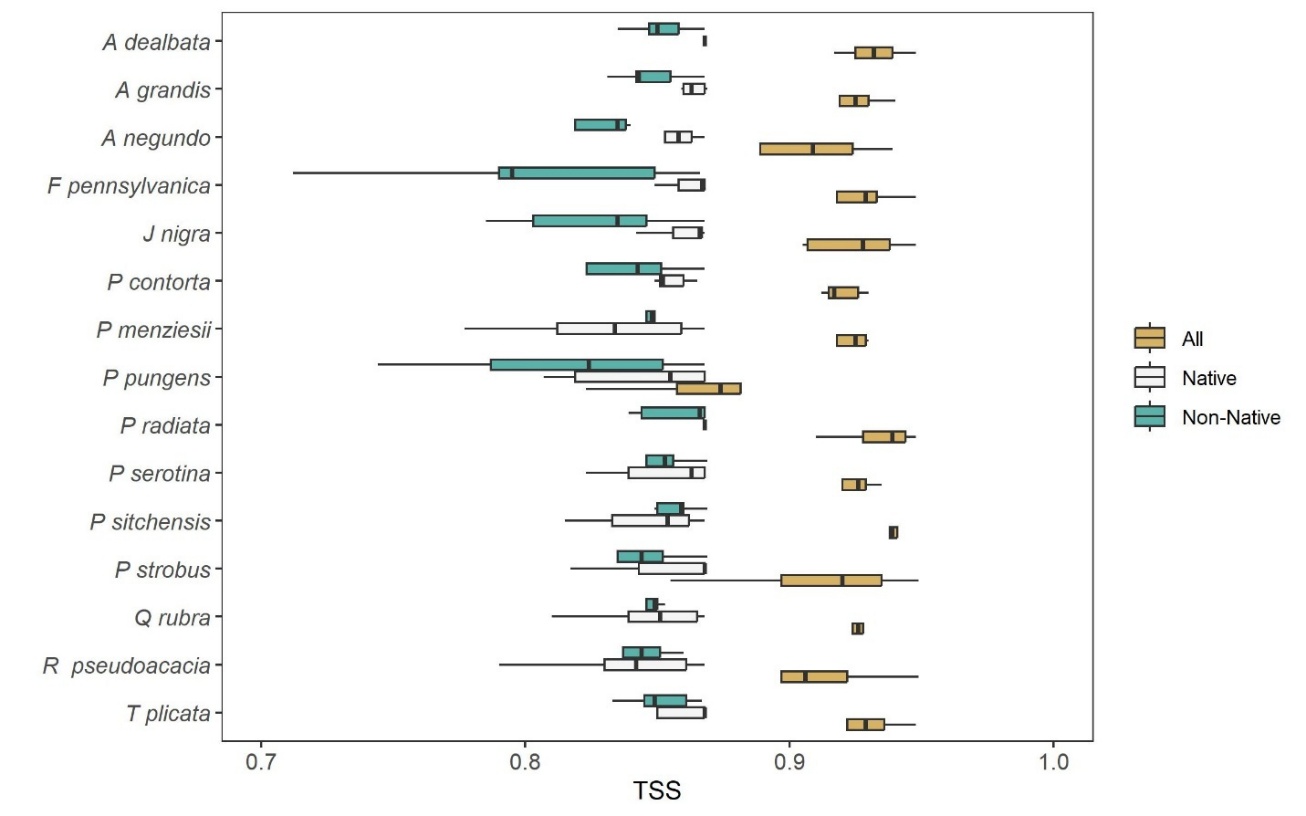
***

### **Fig. S2** True skill statistics (TSS) values of the three sets of SDMs calibrated with occurrence data from the native range, introduced or non-native range in Europe, and the combination of native and non-native range (All). Boxplot center, hinges, and whiskers denote median, upper/lower quartiles, and upper/lower deciles, respectively.


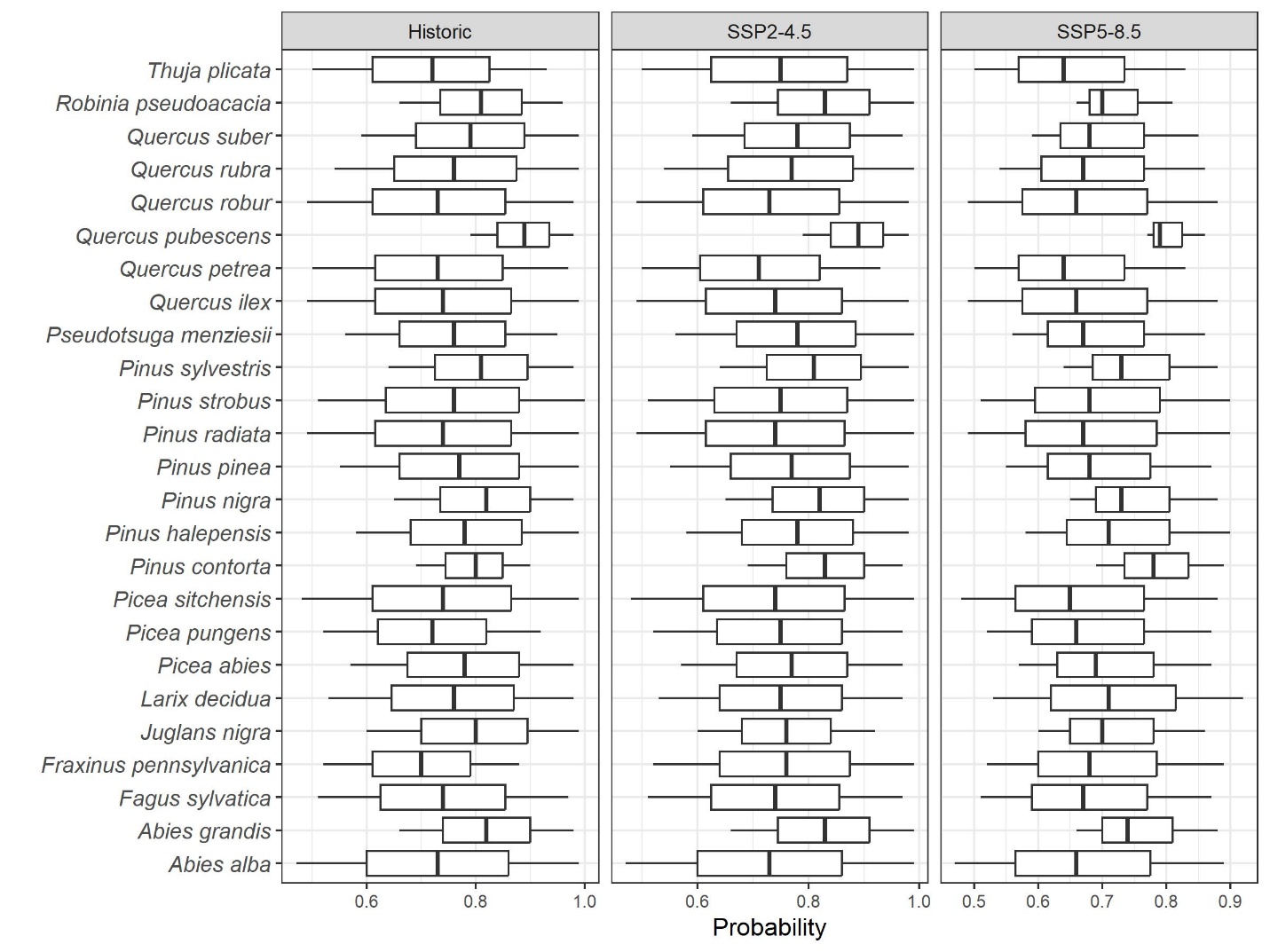


**Fig. S3** Range of probability values for the best-suited native and NNTs under historic climate and two climate change scenarios, SSP2-4.5 and SSP5-8.5, for the time frame 2061-80. Boxplot centre, hinges and whiskers denote median, upper/lower quartiles and upper/lower deciles, respectively.
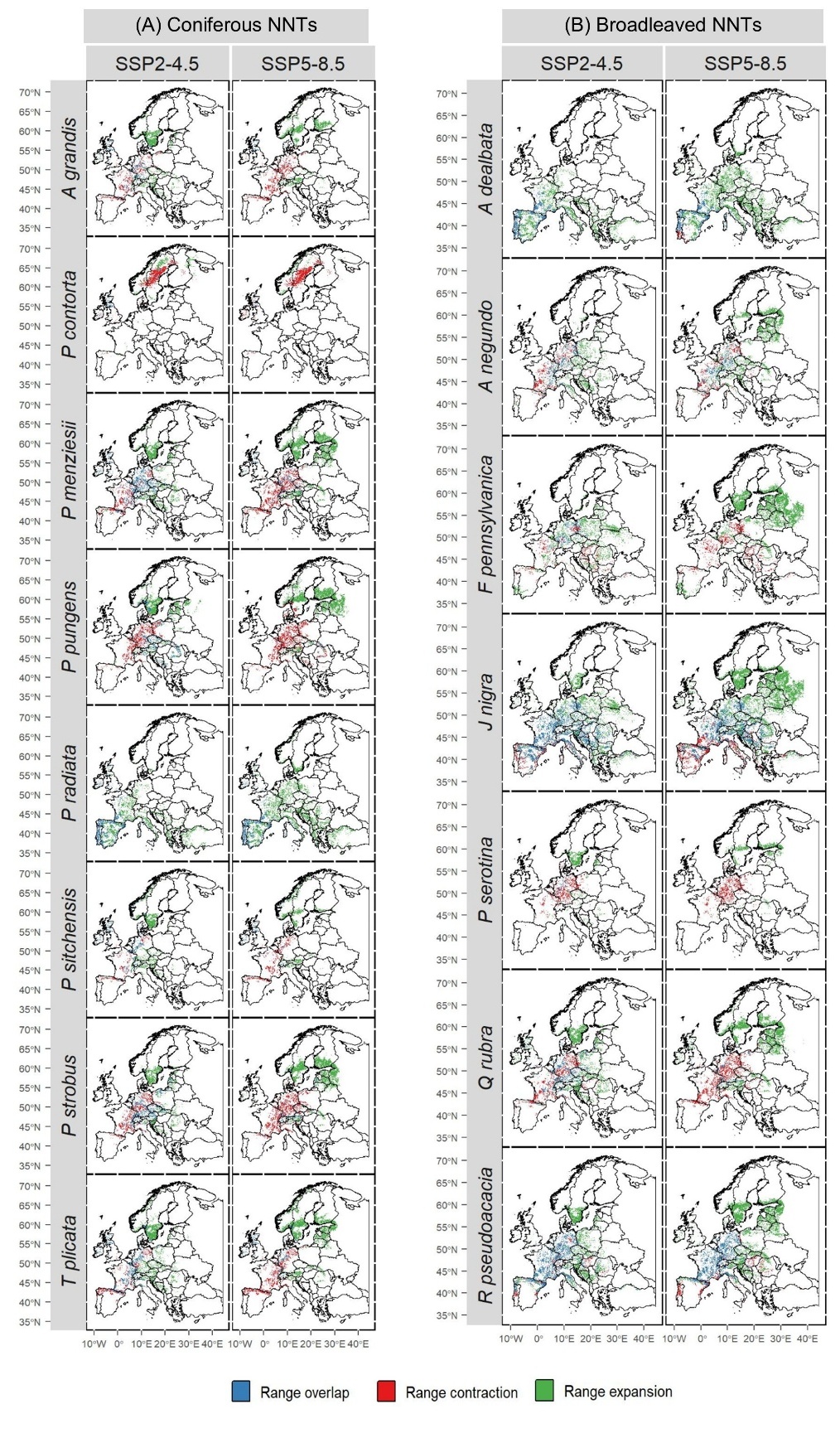


### **Fig. S4** Range shift maps of (A) coniferous NNTs and (B) Broadleaved NNTs. Range shift (overlap, contraction, and expansion) refers to changes in potential species range between the historic period (1961-90) and the future time frame 2061-80 under two climate change scenarios, SSP2-4.5 and SSP5-8.5.


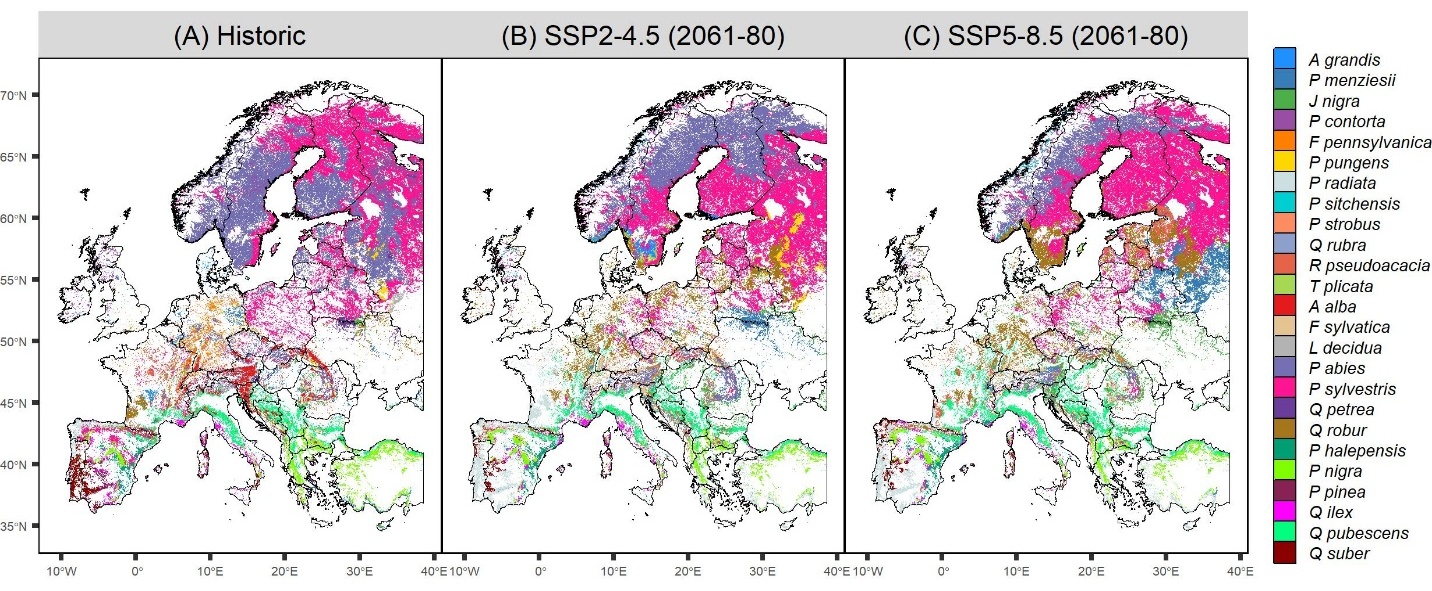


### **Fig. S5** Potential climate suitability depicting forested grid cells with native and NNT species predicted to have the highest climatic suitability in the historic climate and two future time frames under SSP2-4.5 and SSP5-8.5 scenarios.

**
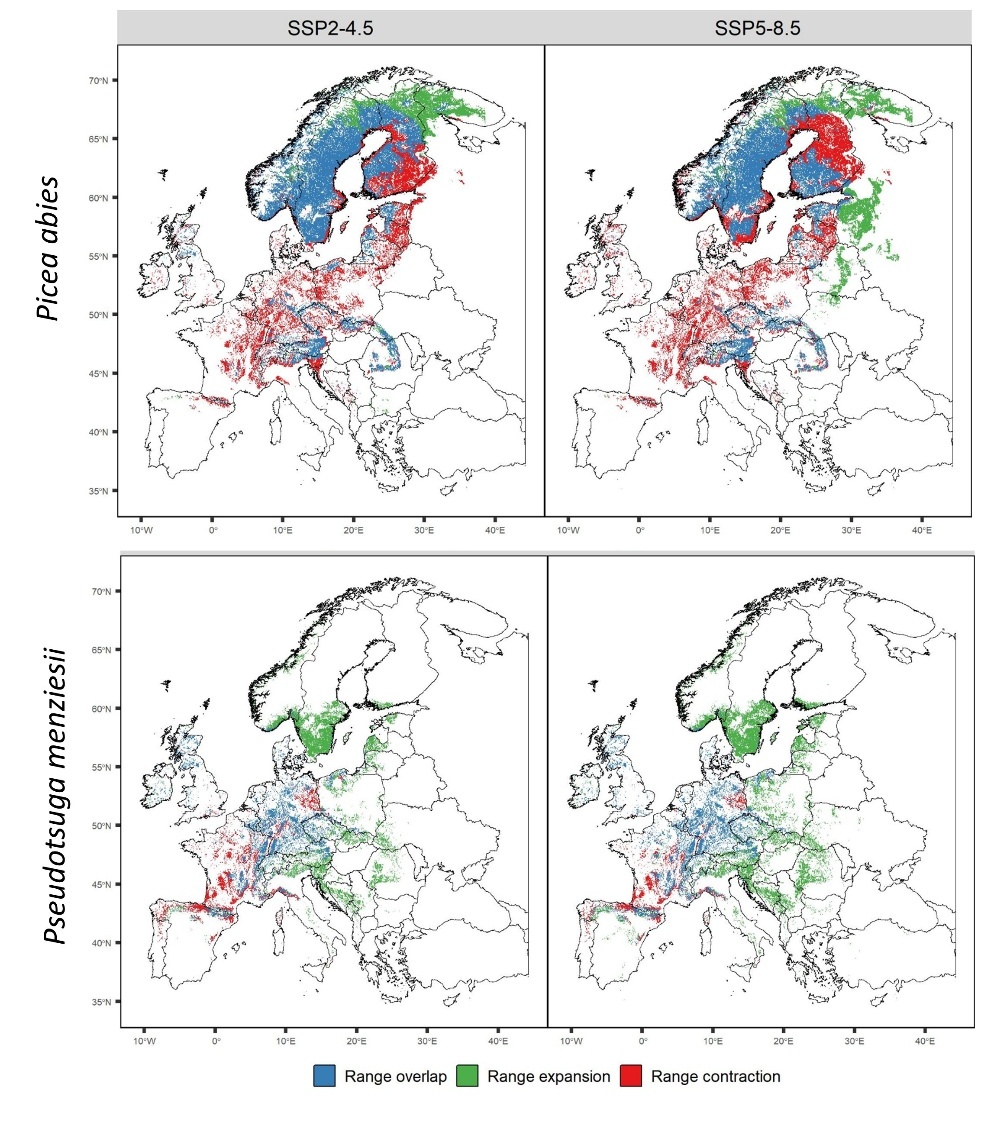
**

### **Fig. S6.** Comparison of range shift between widely occurring native conifer *Picea abies* and a potential alternative NNT, *Pseudotsuga menziesii*

*
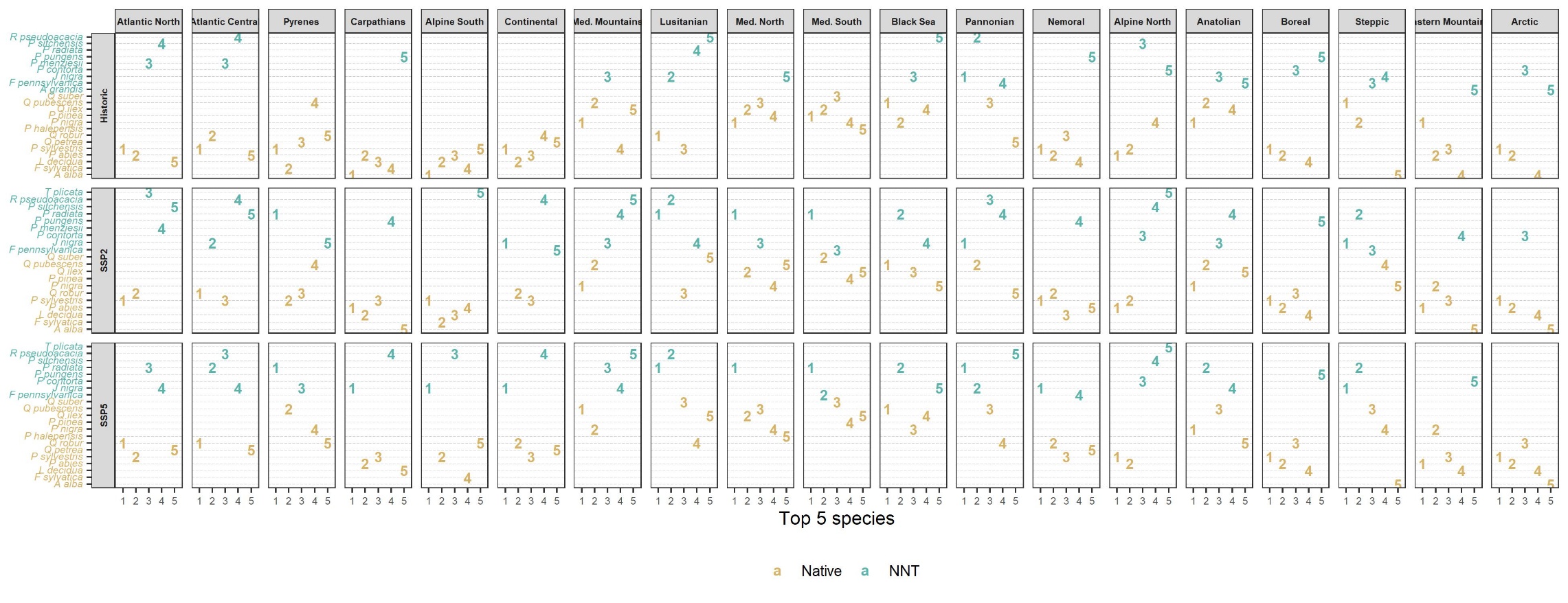
*

### **Fig. S7** Top 5 native and NNT species within each bioclimatic region of Europe under historic (1961-90) and SSP2 and SSP5 scenarios in 2061-80.

*
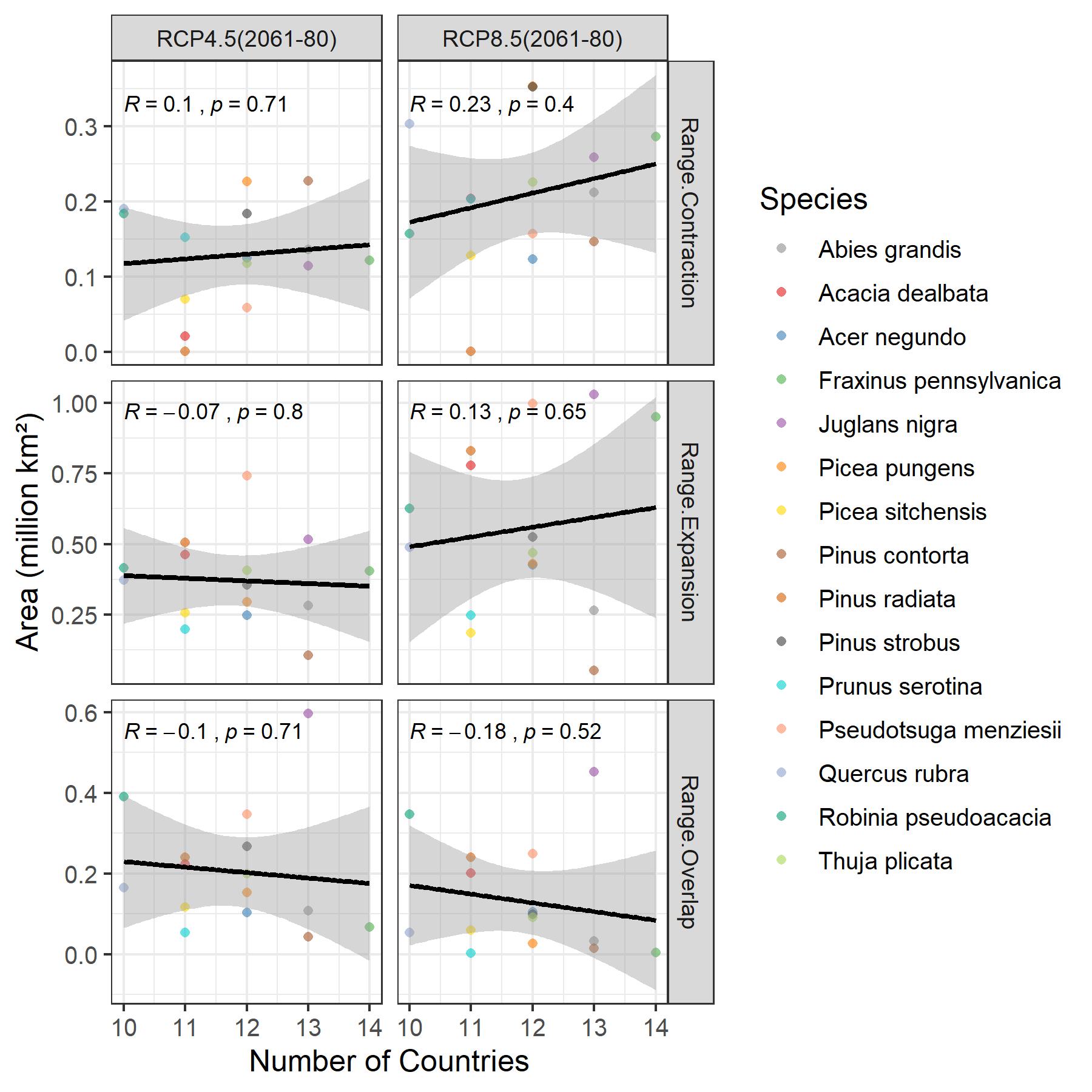
*

### **Fig. S8** Relation between potential range shifts (contraction, overlap, and expansion) and geographic spread of the occurrence data represented by the number of countries covered by the occurrence data.

**
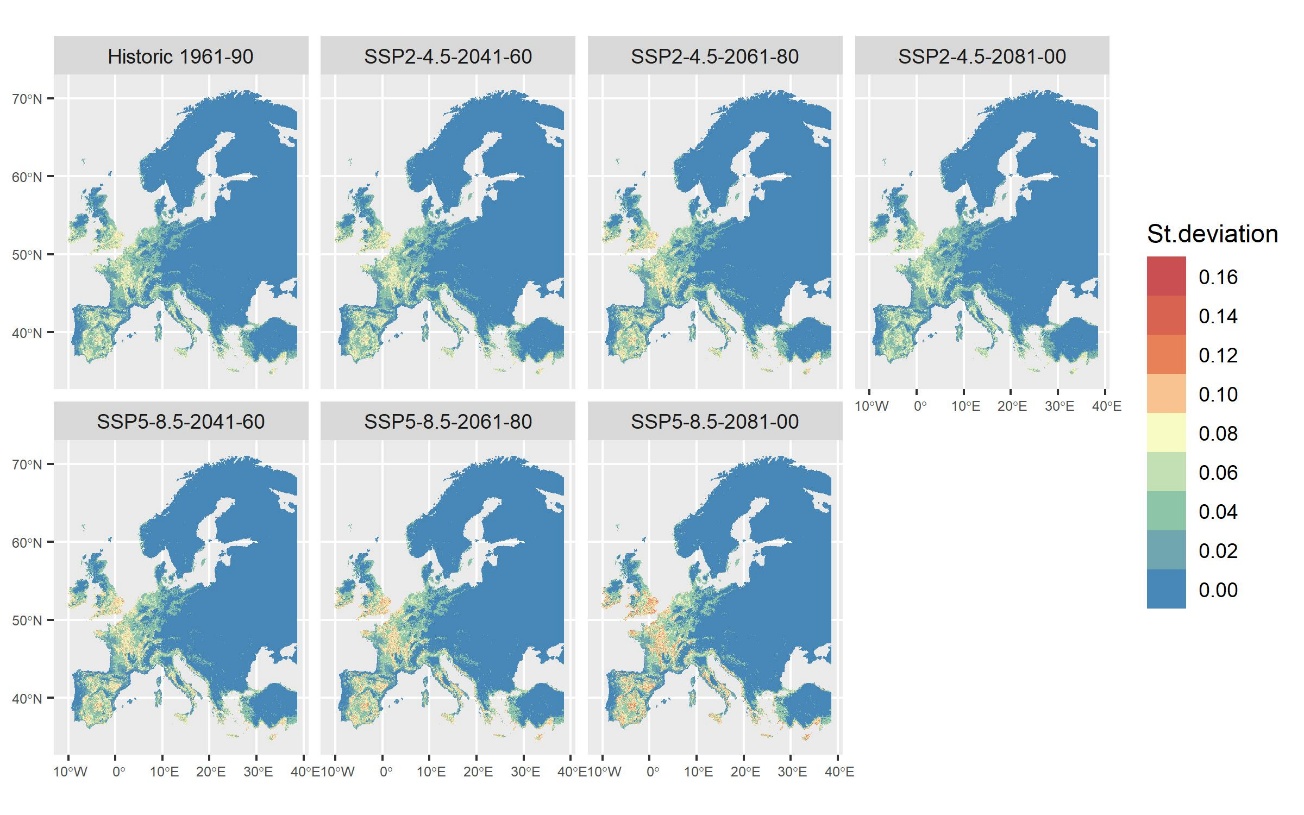
**

### **Fig. S9** Uncertainty expressed in standard deviation of probability ranging from 0 to1 in the predicted potential distribution of *Acacea delabata* under historic and future scenarios. The analysis presented in this paper is based only on Historic and SSP2-4.5 2061-80, and SSP5-8.5 2061-80.

**
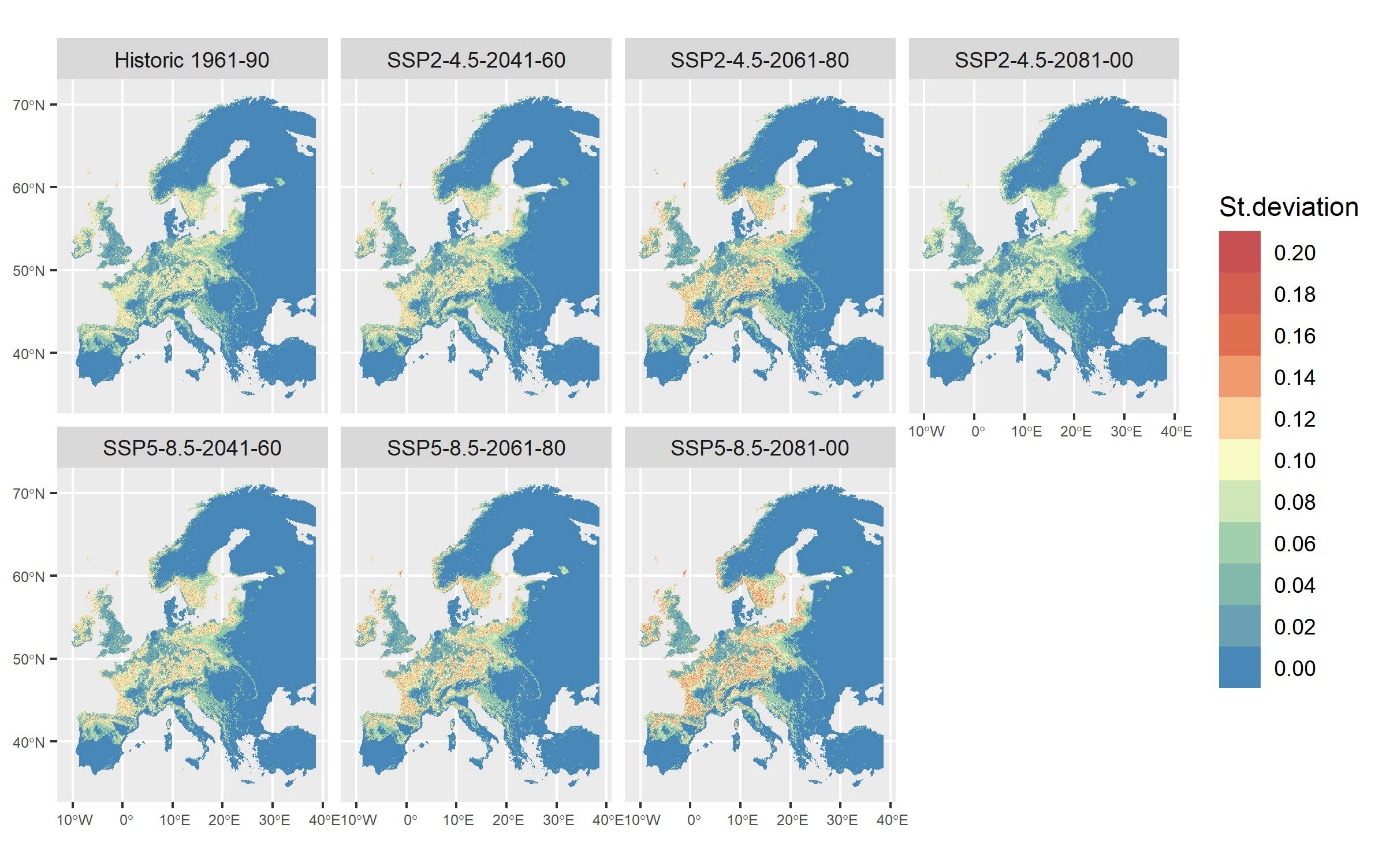
**

### **Fig. S10** Uncertainty expressed in standard deviation of probability ranging from 0 to1 in the predicted potential distribution of Abies grandis under historic and future scenarios. The analysis presented in this paper is based only on Historic and SSP2-4.5 2061-80, and SSP5-8.5 2061-80.

**
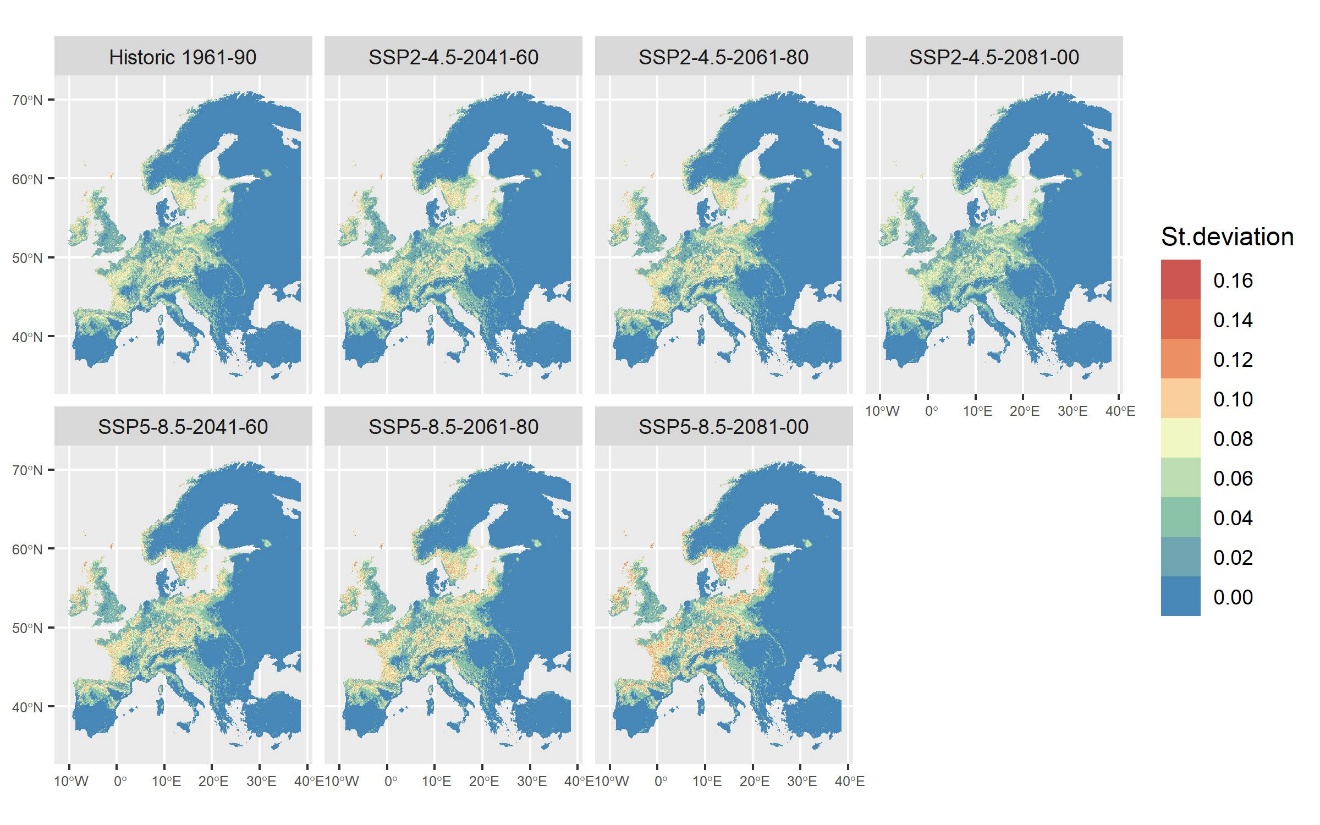
**

### **Fig. S11** Uncertainty expressed in standard deviation of probability ranging from 0 to1 in the predicted potential distribution of *Acer negundo* under historic and future scenarios. The analysis presented in this paper is based only on Historic and SSP2-4.5 2061-80, and SSP5-8.5 2061-80.


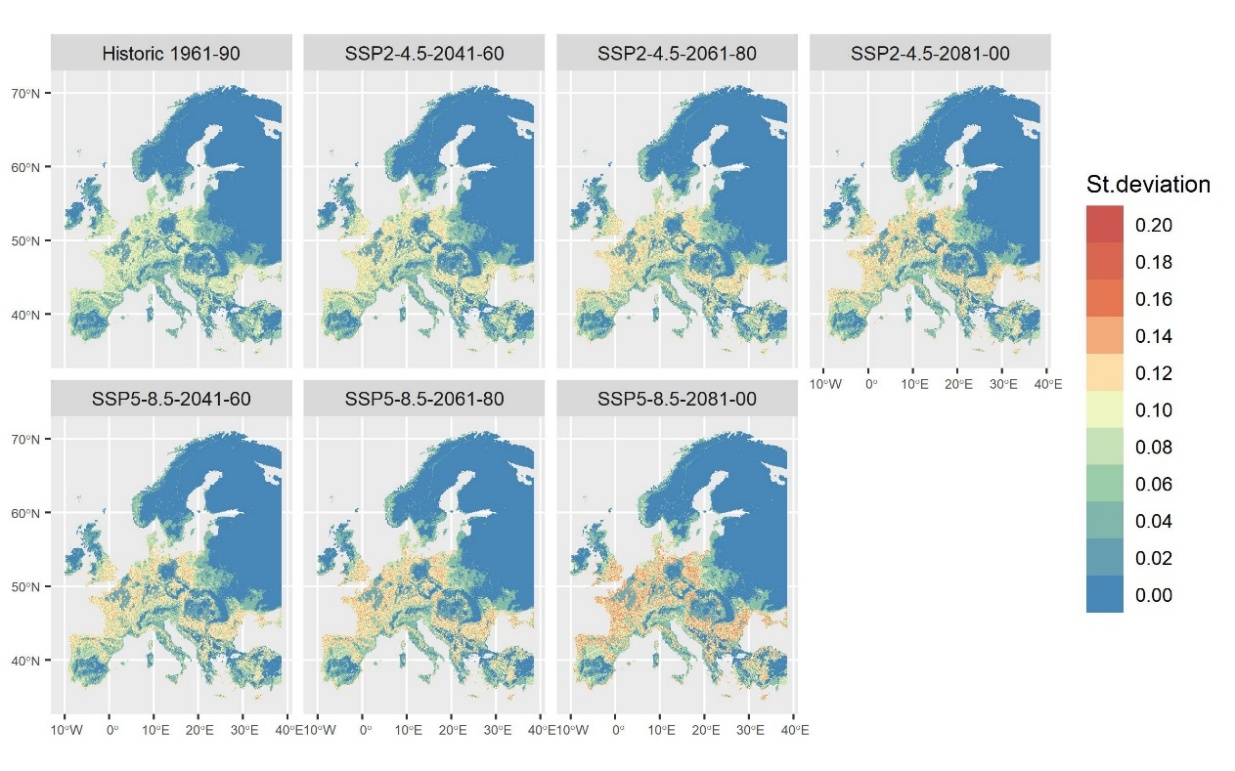


### **Fig. S12** Uncertainty expressed in standard deviation of probability ranging from 0 to1 in the predicted potential distribution of Fraxinus pennsylvanica under historic and future scenarios. The analysis presented in this paper is based only on Historic and SSP2-4.5 2061-80, and SSP5-8.5 2061-80.


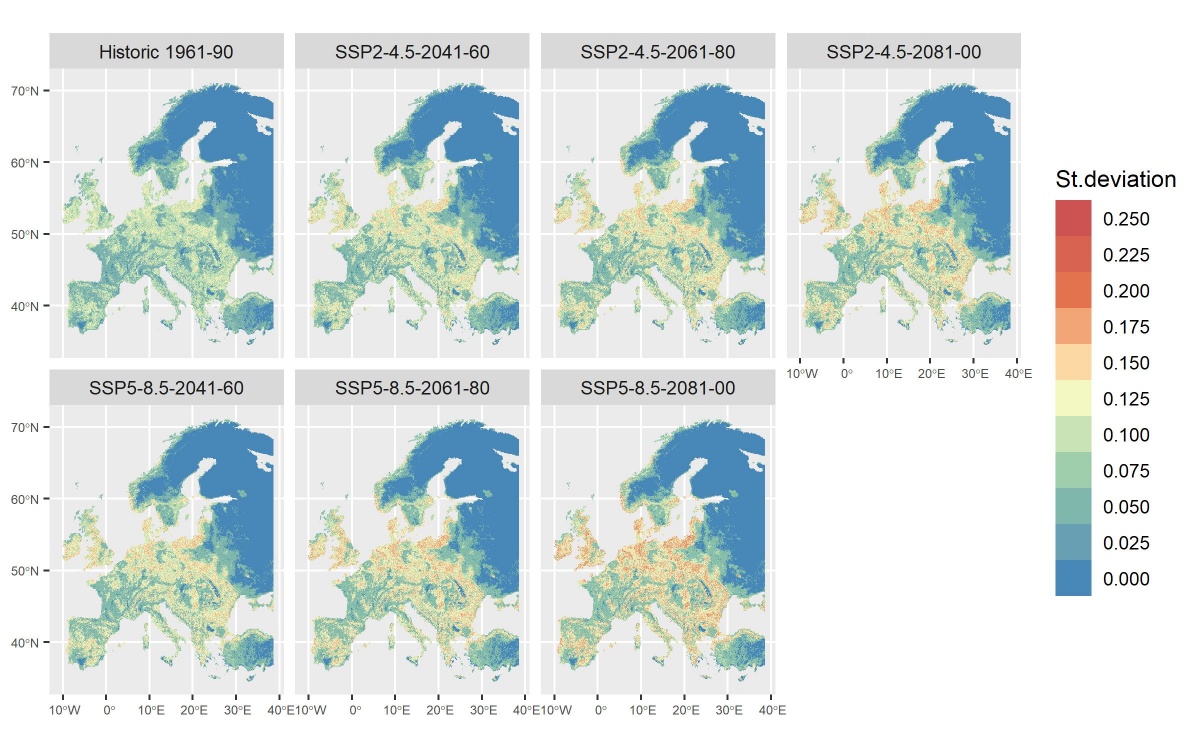


### **Fig. S13** Uncertainty expressed in standard deviation of probability ranging from 0 to1 in the predicted potential distribution of *Juglans nigra* under historic and future scenarios. The analysis presented in this paper is based only on Historic and SSP2-4.5 2061-80, and SSP5-8.5 2061-80.


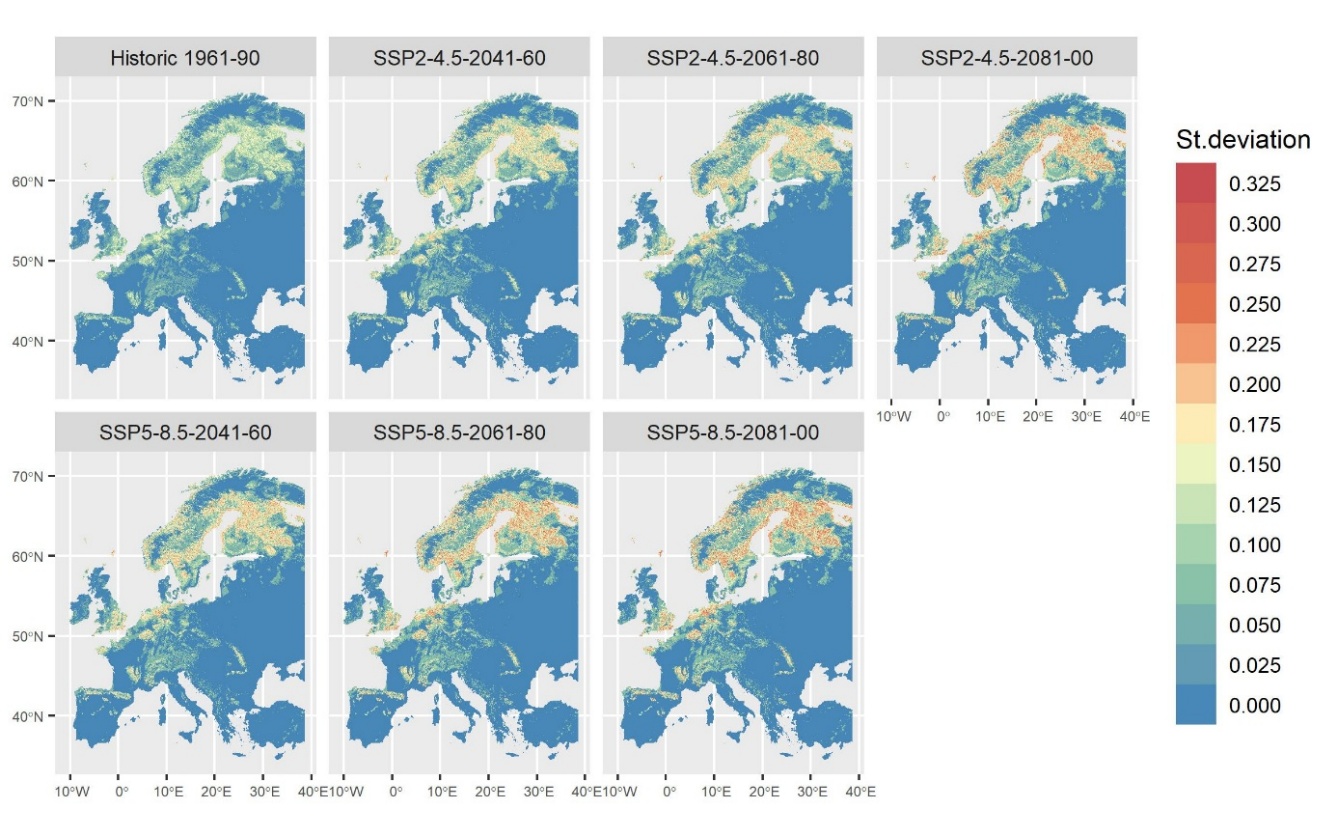


### **Fig. S14** Uncertainty expressed in standard deviation of probability ranging from 0 to1 in the predicted potential distribution of *Pinus contorta* under historic and future scenarios. The analysis presented in this paper is based only on Historic and SSP2-4.5 2061-80, and SSP5-8.5 2061-80.


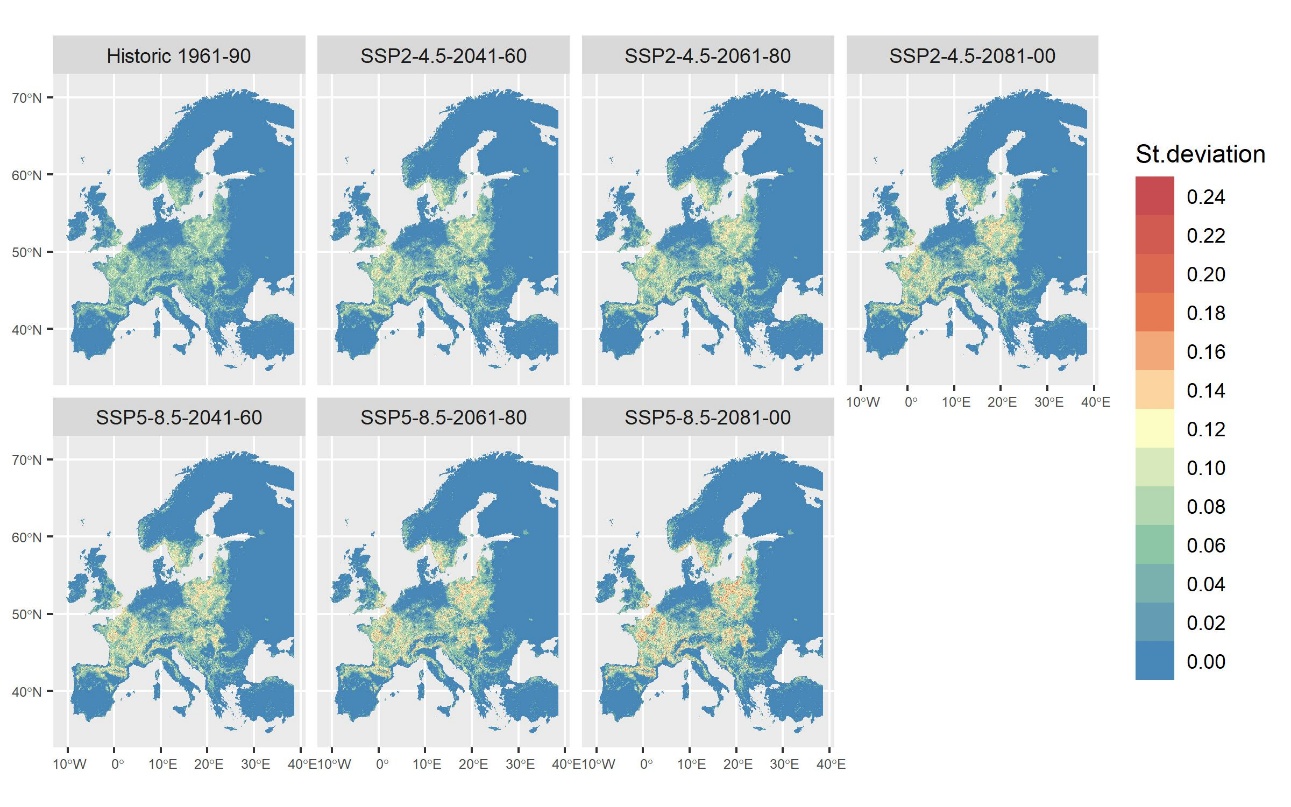


### **Fig. S15** Uncertainty expressed in standard deviation of probability ranging from 0 to1 in the predicted potential distribution of *Prunus serotina* under historic and future scenarios. The analysis presented in this paper is based only on Historic and SSP2-4.5 2061-80, and SSP5-8.5 2061-80.


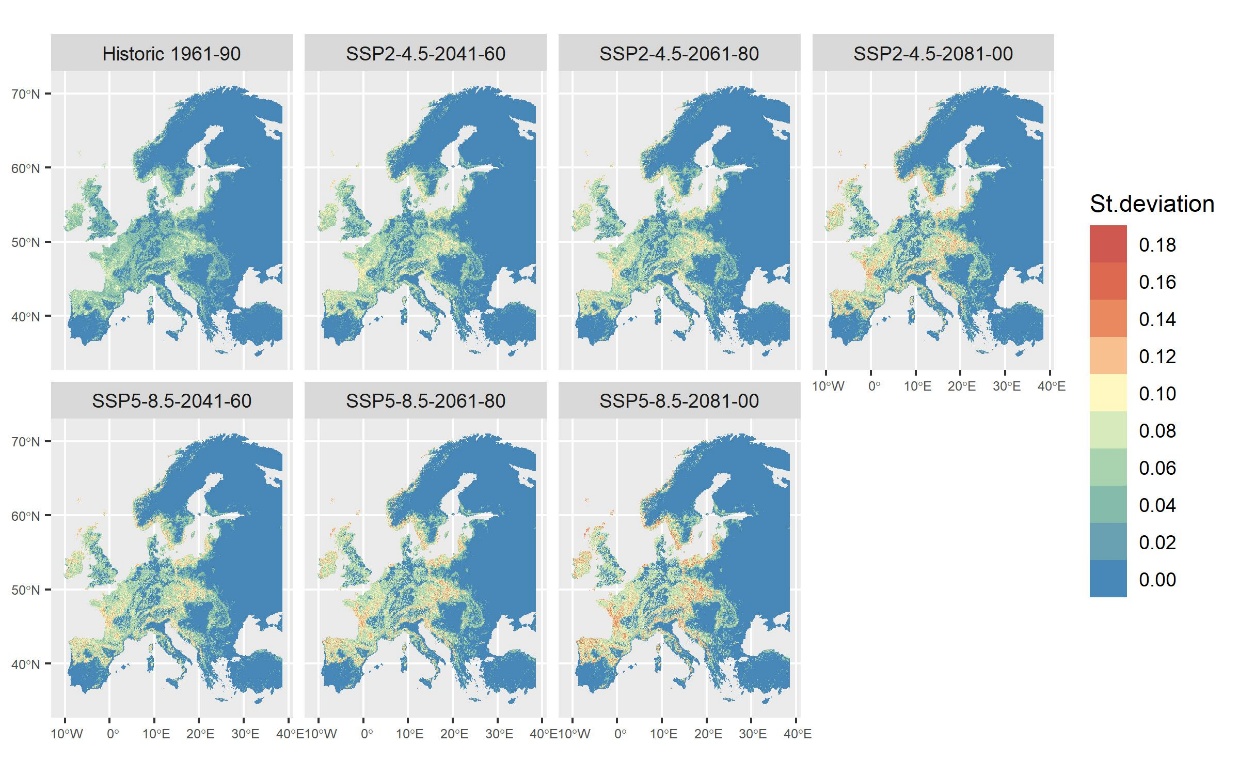


### **Fig. S16** Uncertainty expressed in standard deviation of probability ranging from 0 to1 in the predicted potential distribution of *Pseudotsuga menziesii* under historic and future scenarios. The analysis presented in this paper is based only on Historic and SSP2-4.5 2061-80, and SSP5-8.5 2061-80.


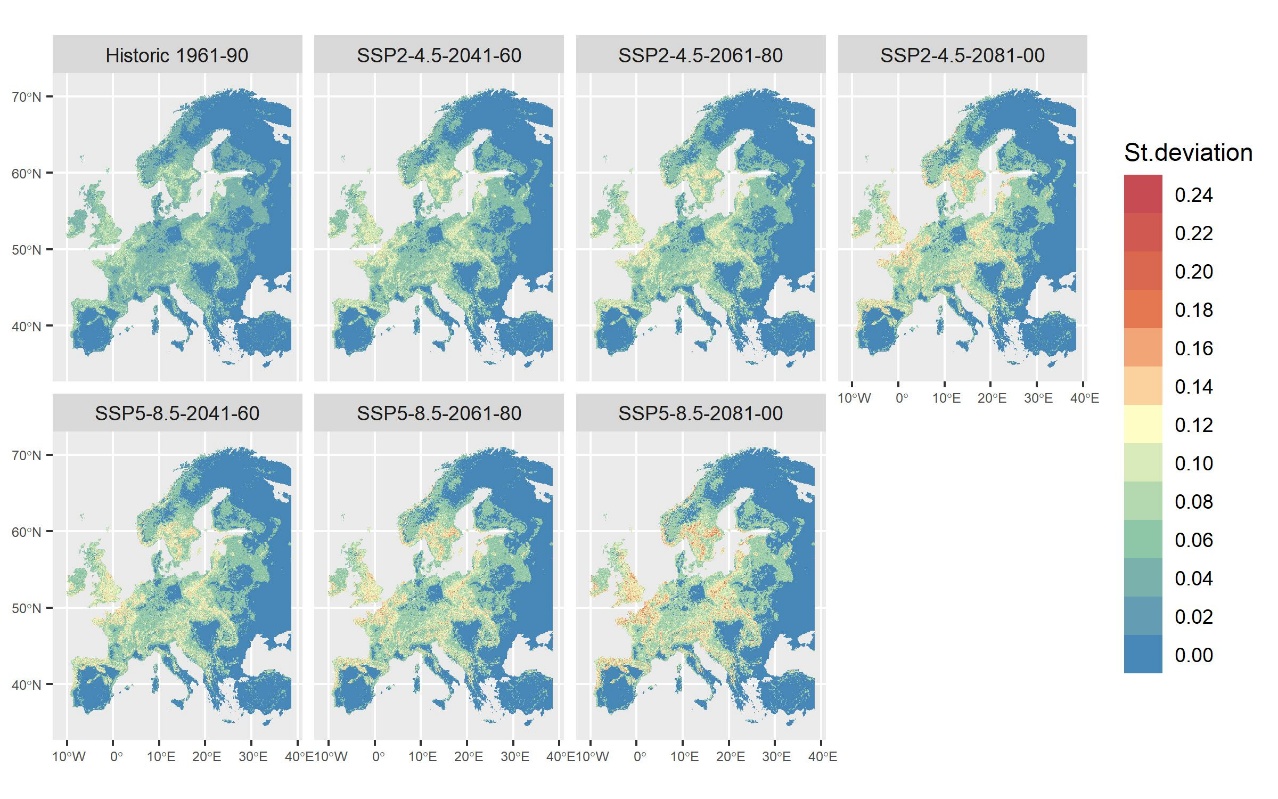


### **Fig. S17** Uncertainty expressed in standard deviation of probability ranging from 0 to1 in the predicted potential distribution of *Picea pungens* under historic and future scenarios. The analysis presented in this paper is based only on Historic and SSP2-4.5 2061-80, and SSP5-8.5 2061-80.


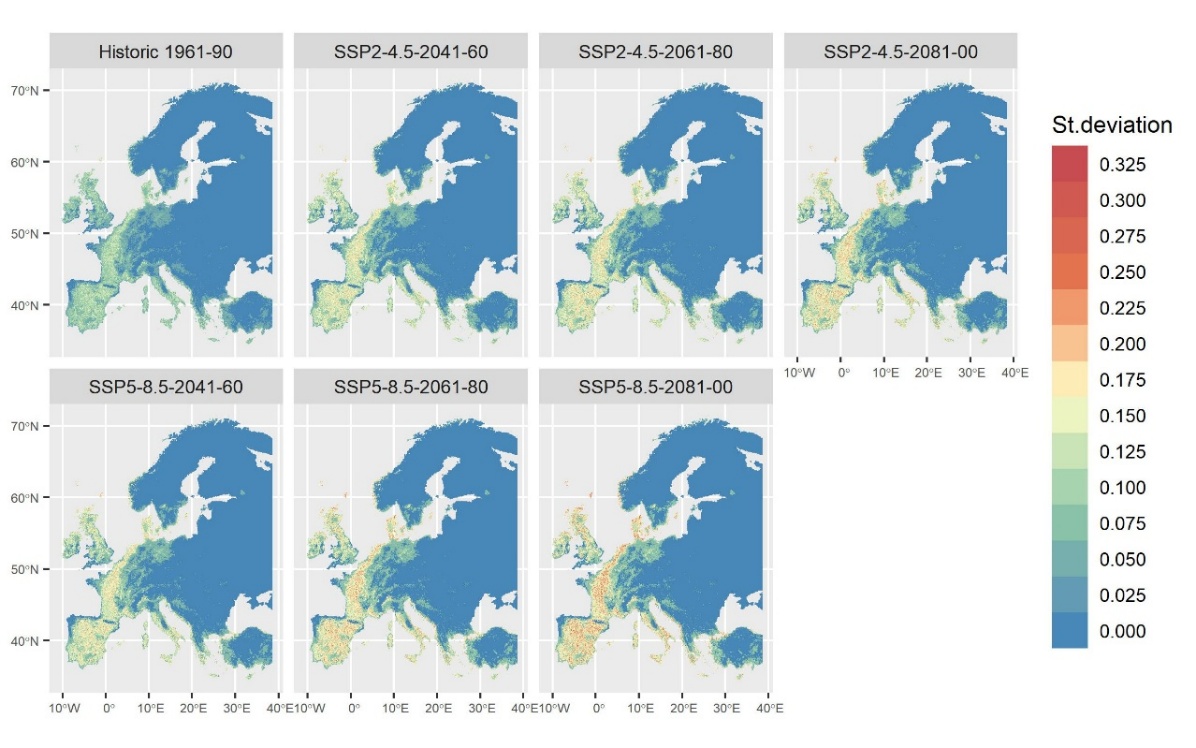


### **Fig. S18** Uncertainty expressed in standard deviation of probability ranging from 0 to1 in the predicted potential distribution of *Pinus radiata* under historic and future scenarios. The analysis presented in this paper is based only on Historic and SSP2-4.5 2061-80, and SSP5-8.5 2061-80.


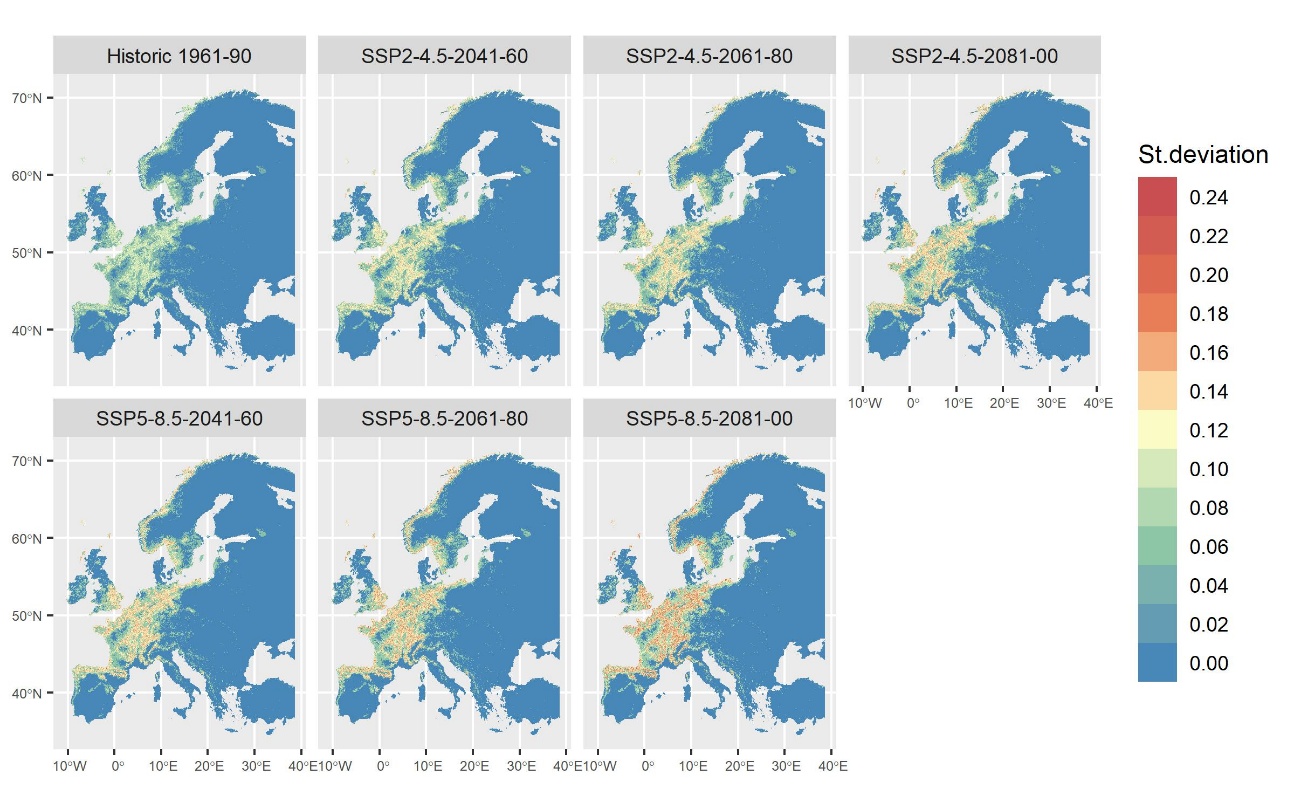


### **Fig. S19** Uncertainty expressed in standard deviation of probability ranging from 0 to1 in the predicted potential distribution of *Picea sitchensis* under historic and future scenarios. The analysis presented in this paper is based only on Historic and SSP2-4.5 2061-80, and SSP5-8.5 2061-80.


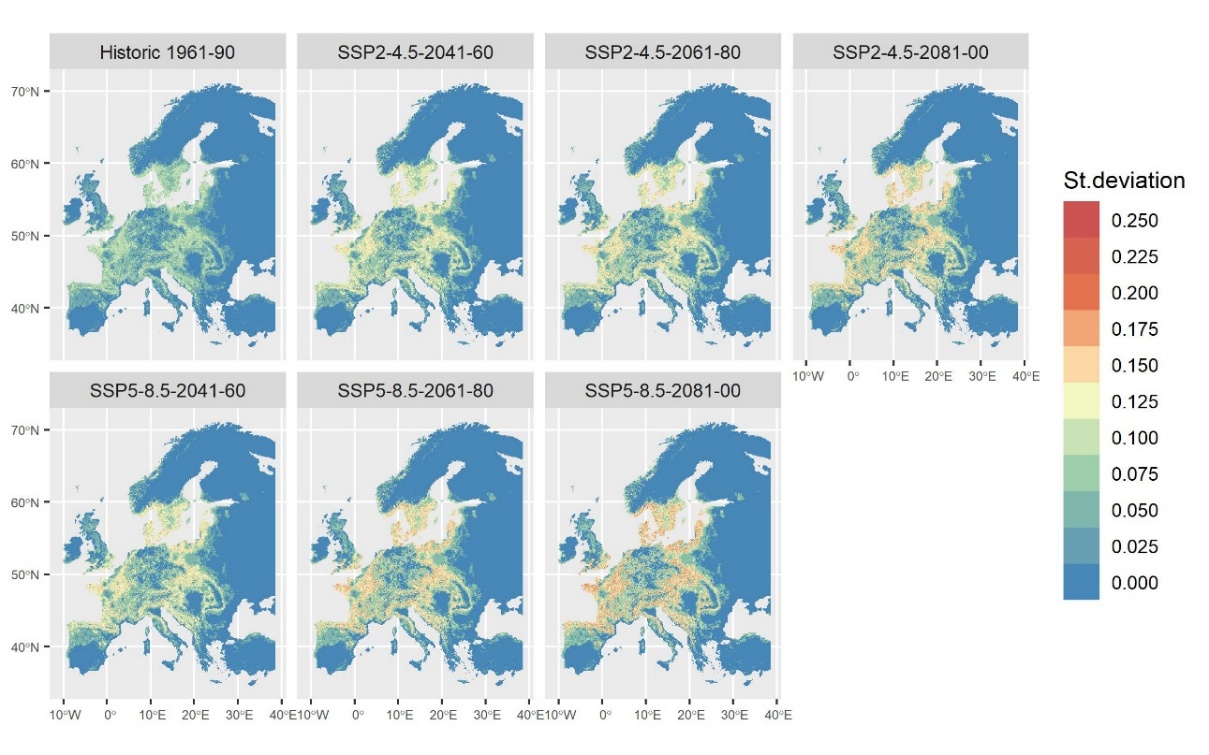


### **Fig. S20** Uncertainty expressed in standard deviation of probability ranging from 0 to1 in the predicted potential distribution of *Pinus strobus* under historic and future scenarios. The analysis presented in this paper is based only on Historic and SSP2-4.5 2061-80, and SSP5-8.5 2061-80.


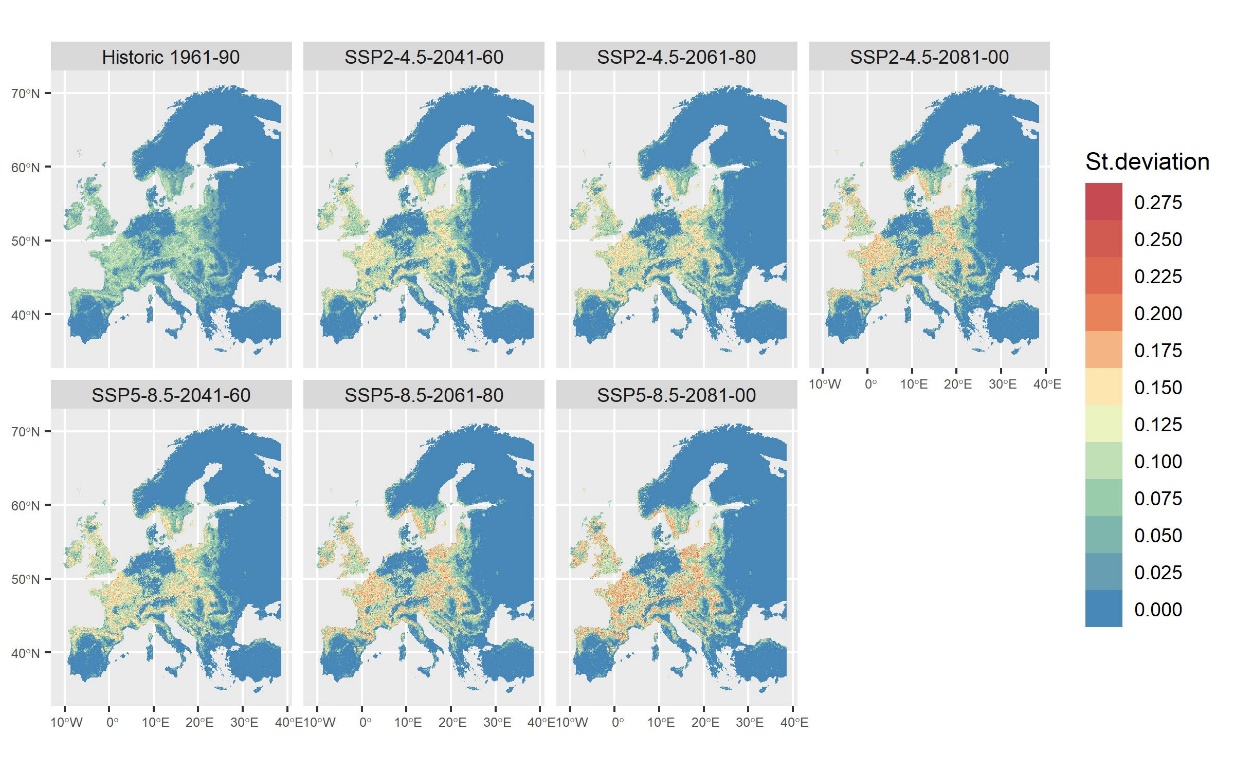


### **Fig. S21** Uncertainty expressed in standard deviation of probability ranging from 0 to1 in the predicted potential distribution of *Quercus rubra* under historic and future scenarios. The analysis presented in this paper is based only on Historic and SSP2-4.5 2061-80, and SSP5-8.5 2061-80.


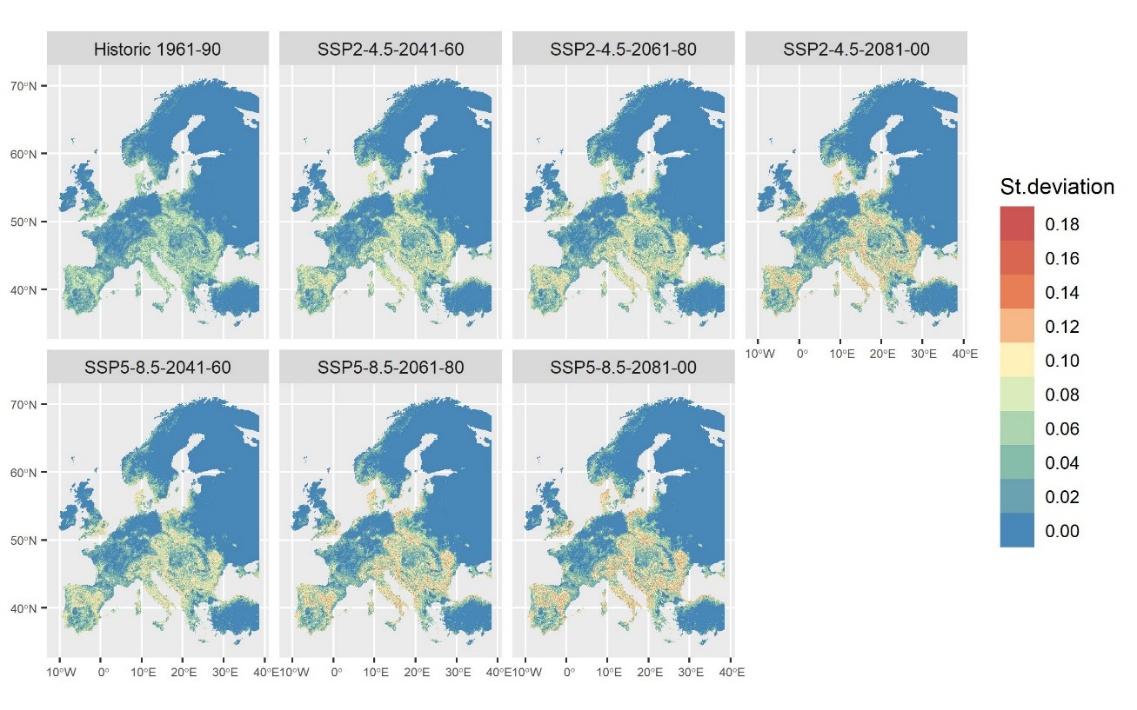


### **Fig. S22** Uncertainty expressed in standard deviation of probability ranging from 0 to1 in the predicted potential distribution of Robinia pseudoacacia under historic and future scenarios. The analysis presented in this paper is based only on Historic and SSP2-4.5 2061-80, and SSP5-8.5 2061-80.


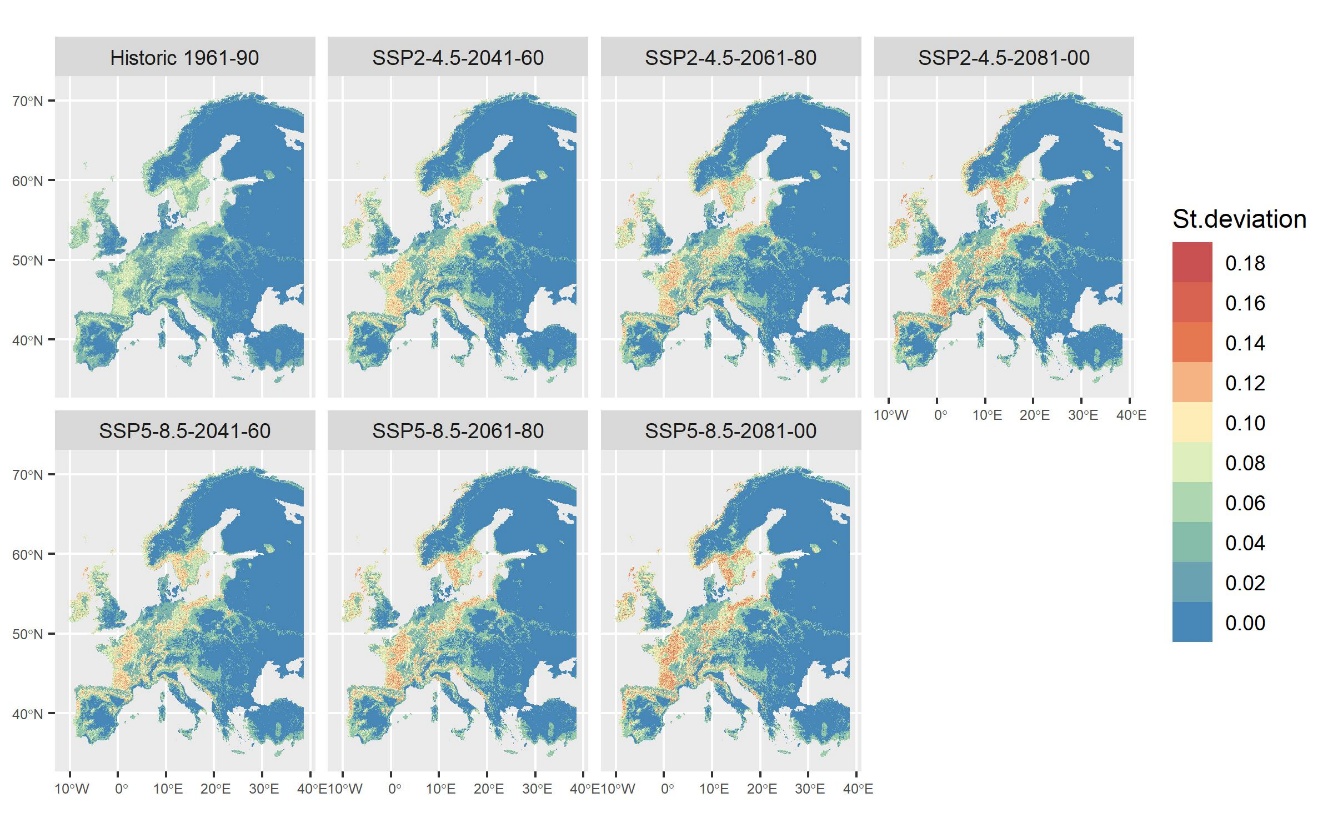


### **Fig. S23** Uncertainty expressed in standard deviation of probability ranging from 0 to1 in the predicted potential distribution of *Thuja plicata* under historic and future scenarios. The analysis presented in this paper is based only on Historic and SSP2-4.5 2061-80, and SSP5-8.5 2061-80.

### **References**

Akaike H (1974) A new look at the statistical model identification. *IEEE Transactions on Automatic Control* **19**(6): 716–723. <https://doi.org/10.1109/TAC.1974.1100705>

Allouche O, Tsoar A, Kadmon R (2006) Assessing the accuracy of species distribution models: prevalence, kappa and the True Skill Statistic (TSS). *Journal of Applied Ecology* **43**(6): 1223–1232. <https://doi.org/10.1111/j.1365-2664.2006.01214.x>

Barbet‐Massin M, Jiguet F, Albert CH, Thuiller W (2012) Selecting pseudo‐absences for species distribution models: how, where and how many? *Methods in Ecology and Evolution* **3**(2): 327–338. <https://doi.org/10.1111/j.2041-210X.2011.00172.x>

Booth GD, Niccolucci MJ, Schuster EG (1994) Identifying proxy sets in multiple linear regression: an aid to better coefficient interpretation. *USDA Forest Service Intermountain Research Station Research Paper*.

Breiman L (2001) Random forests. *Machine Learning* **45**(1): 5–32. <https://doi.org/10.1023/A:1010933404324>

Chakraborty, D., Ciceu, A., Ballian, D. et al (2024a). Assisted tree migration can preserve the European forest carbon sink under climate change. Nat. Clim. Chang. 14, 845–852 (2024). <https://doi.org/10.1038/s41558-024-02080-5>

Chakraborty, D etal. (2024b). Dataset on Potential Distribution of Non-native Tree species in Europe (Version Version1) [Data set]. Zenodo. <https://doi.org/10.5281/zenodo.10782504>

Chakraborty D, Dobor L, Hlásny T, Schueler S (2021) High‐resolution gridded climate data for Europe based on bias‐corrected EURO‐CORDEX: the ECLIPS‐2.0 dataset. *Zenodo*. <https://doi.org/10.5281/zenodo.3952159>

Chakraborty D, Móricz N, Rasztovits E, et al (2021b). Provisioning forest and conservation science with high-resolution maps of potential distribution of major European tree species under climate change. Ann For Sci 78:. <https://doi.org/10.1007/s13595-021-01029-4>

Coetzee BWT, Robertson MP, Erasmus BFN, van Rensburg BJ, Thuiller W (2009) Ensemble models predict Important Bird Areas in southern Africa will become less effective for conserving endemic birds under climate change. *Global Ecology and Biogeography* **18**(6): 701–710. <https://doi.org/10.1111/j.1466-8238.2009.00485.x>

Fick SE, Hijmans RJ (2017) WorldClim 2: new 1‐km spatial resolution climate surfaces for global land areas. *International Journal of Climatology* **37**(12): 4302–4315. <https://doi.org/10.1002/joc.5086>

Häggström C, et al. (2015) covsel: A method for covariate selection in ecological models. *R package documentation*.

Heiderer R, et al. (2011) *Forest Focus and Biosoil datasets*. European Commission Joint Research Centre, Ispra.

Mauri A, Strona G, San‐Miguel‐Ayanz J (2017) EU‐Forest, a high‐resolution tree occurrence dataset for Europe. *Scientific Data* **4**: 160123. <https://doi.org/10.1038/sdata.2016.123>

Pontius RG Jr, Peethambaram S, Castella JC (2014) Comparison of methods to assess the accuracy of land change simulations. *Ecological Modelling* **293**: 81–90. https://doi.org/10.1016/j.ecolmodel.2014.10.007

R Core Team (2016) *R: A Language and Environment for Statistical Computing*. R Foundation for Statistical Computing, Vienna, Austria.

Riahi K, van Vuuren DP, Kriegler E, Edmonds J, O’Neill BC, Fujimori S, Bauer N, et al. (2017) The Shared Socioeconomic Pathways and their energy, land use, and greenhouse gas emissions implications: an overview. *Global Environmental Change* **42**: 153–168. <https://doi.org/10.1016/j.gloenvcha.2016.05.009>

Thuiller W, Georges D, Gueguen M, Engler R, Breiner F, Lafourcade B, Patin R, Blancheteau H (2016) *biomod2: Ensemble platform for species distribution modeling*. R package version 3.3-2. <https://biomodhub.github.io/biomod2>

Zurell D, Franklin J, König C, Bouchet PJ, Dormann CF, Elith J, Fandos G, Feng X, Guillera‐Arroita G, Guisan A, Lahoz‐Monfort JJ, Leitão PJ, Park DS, Peterson AT, Rapacciuolo G, Schmatz DR, Schröder B, Serra‐Diaz JM, Thuiller W, Yates KL, Zimmermann NE, Merow C (2020) A standard protocol for reporting species distribution models. *Ecography* **43**(9): 1261–1277. https://doi.org/10.1111/ecog.04960
